# Heterogeneity in adverse events related to atezolizumab-bevacizumab for hepatocellular carcinoma reported in real-world studies

**DOI:** 10.1016/j.jhepr.2024.101190

**Published:** 2024-08-22

**Authors:** Claudia Campani, Dimitrios Pallas, Sabrina Sidali, Olga Giouleme, Lorraine Blaise, Véronique Grando, Gisele Nkontchou, Alix Demory, Pierre Nahon, Nathalie Ganne-Carrié, Jean-Charles Nault

**Affiliations:** 1 Cordeliers Research Center, Sorbonne University, Inserm, Paris Cité University, “Functional Genomics of Solid Tumors” Team, Ligue Nationale Contre le Cancer Accredited Team, Labex OncoImmunology, F-75006 Paris, France; 2 Internal Medicine and Hepatology Unit, Department of Experimental and Clinical Medicine, University of Firenze, Florence, Italy; 3 Liver Unit, Avicenne Hospital, APHP, Bobigny, France, University Sorbonne Paris Nord, Bobigny, France; 4 Department of Gastroenterology and Hepatology, 2nd Propedeutic Department of Internal Medicine, Medical School, Aristotle University, 54124 Thessaloniki, Greece

**Keywords:** liver cancer, immunotherapy, adverse effects

## Abstract

**Background & Aims:**

Safety data for patients with hepatocellular carcinoma (HCC) treated with atezolizumab-bevacizumab in the real-world setting remain uncertain. Thus, the aim of this study was to evaluate the incidence of adverse events (AEs) in patients with HCC treated with atezolizumab-bevacizumab in the literature.

**Methods:**

In this systematic review and meta-analysis, we searched PubMed for original studies reporting percentages of AEs in patients with HCC receiving atezolizumab-bevacizumab between 2020 to 2023, using the search terms “Atezolizumab/Bevacizumab”, “HCC” and “Adverse events”. We summarized the incidence of AEs and performed a meta-analysis in order to evaluate the incidence of AEs reported in the literature.

**Results:**

A total of 30 studies (3,867 patients) were included. The analysis revealed heterogeneity in AE reporting, with arterial hypertension, proteinuria, and fatigue being the most frequently reported AEs whereas incidence of bleeding was reported in 66.7% of the studies and rare immune-related AEs were reported in 26.7% of the studies. The meta-analysis revealed pooled incidence rates of 79% for any grade AEs: 56% for grade 1/2 and 30% for grade ≥3. While the pooled rates of hypertension, anorexia, bleeding, pruritus, rash, and thyroid dysfunction were similar to those reported in the IMbrave150 trial, higher rates were observed in the literature for proteinuria, fatigue, ALT and AST elevations and gastrointestinal perforation. For grade ≥3 AEs, the percentages were consistent with the IMbrave150 trial, except for lower incidences of arterial hypertension and thrombosis in the literature. The exposure-adjusted incidence rates for proteinuria (55.7%), hypertension (45.3%) and fatigue (33.6%) were high. Heterogeneity was observed in the analysis of AEs across articles within the same cohorts of patients.

**Conclusion:**

We observed a significant variability in AE reporting for atezolizumab-bevacizumab treatment in HCC in the literature, underscoring the need for standardized reporting practices.

**Impact and implications:**

Considering the demonstrated safety of atezolizumab-bevacizumab in randomized-controlled trials, this meta-analysis offers valuable insights into reported occurrences of adverse events. Our study highlights significant heterogeneity among studies, underscoring the need to improve adverse event recording. Understanding the incidence and severity of treatment-related adverse events beyond clinical trials is essential for prompt intervention and may help in preventing treatment discontinuation and complications, potentially leading to better outcomes without significantly compromising quality of life due to adverse events.

## Highlights

• Systematic review analyzed 30 studies on 3,867 patients with HCC treated with atezolizumab-bevacizumab.
• High variability in adverse event reporting across studies available in the literature.
• Severe adverse events like bleeding, thrombosis, and perforation are often underreported.
• Need for standardized and thorough adverse event reporting in clinical practice.

## Graphical abstract

**Figure:**
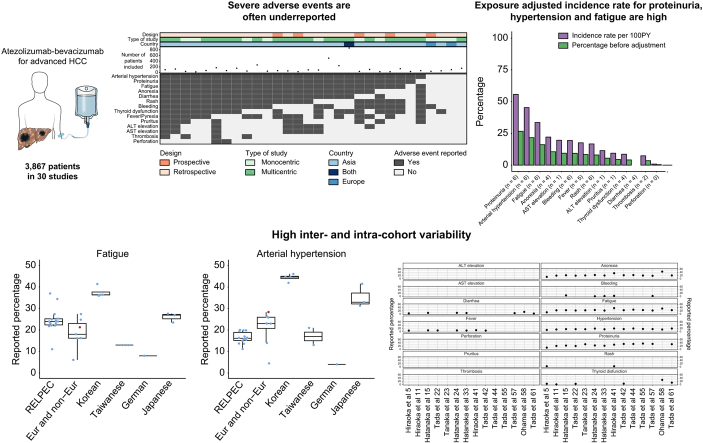

## Introduction

The combination of monoclonal antibodies targeting PD-L1 (programmed death-ligand 1) (atezolizumab) and VEGF (vascular endothelial growth factor) (bevacizumab) represents the new first-line standard of care for patients with unresectable hepatocellular carcinoma (HCC).^1^^,^^2^ In the IMbrave150 trial, the atezolizumab-bevacizumab combination demonstrated improvements in overall survival (OS), progression-free survival (PFS) and objective response rate, as well as patient-reported outcomes compared to sorafenib.^3^^,^^4^ Adverse events (AEs) of any grade occurred in 98% of patients treated with atezolizumab-bevacizumab *vs.* 99% in patients treated with sorafenib with 49% experiencing severe AEs *vs.* 33% in the sorafenib arm. The most common treatment-related AEs were proteinuria, arterial hypertension, increased aspartate aminotransferase (AST), and fatigue with a percentage of AEs leading to treatment withdrawal of 22%.^3^^,^^4^ The most common immune-mediated AEs were rash (22%) and thyroid dysfunction (19%).^3^^,^^4^ While a high rate of AST increase was also observed (53%), not all cases could be adjudicated to atezolizumab and immune-related hepatitis. Moreover, the percentage of corticosteroid use for immune-related AEs was not reported. In the atezolizumab-bevacizumab arm, there were six cases of bleeding, five gastrointestinal and one non-gastrointestinal hemorrhage, with a single case of intraperitoneal hemorrhage among patients treated with sorafenib. However, eligibility for IMbrave 150 trial was restricted to patients without prior exposure to systemic therapy, with preserved liver function (Child-Pugh A), and optimal control of portal hypertension.^3^^,^^4^ For this reason, the safety data remain uncertain for patients treated with atezolizumab-bevacizumab in the real-world setting, including rare AEs such as gastrointestinal perforation, bleeding, thrombosis and immune-related AEs requiring corticosteroids.[5], [6], [7], [8] Accurate and consistent reporting of AEs is crucial for evaluating the safety profile of any therapeutic regimen, guiding clinical decision-making, and informing patient management. The analysis of incidence of AEs in studies from patients treated in the real-world setting is also useful to identify potential new toxicity signals not reported in the phase III clinical trials. The incidence of the different AEs associated with systemic treatments in other solid cancers across studies published in the literature is highly heterogeneous, both for lower grade and more severe AEs.[9], [10], [11] Inconsistencies in the reporting of AEs can stem from differences in study design (retrospective *vs.* prospective, monocentric, multicentric), sample size, patient demographics, and the way AEs are collected. These discrepancies complicate the interpretation of safety data and can obscure the true incidence and severity of AEs. However, this issue was not studied in patients with advanced HCC treated with systemic treatment.

We aimed to perform a systematic review and meta-analysis of the literature to assess the frequencies of AEs reported in studies evaluating atezolizumab-bevacizumab in patients with advanced HCC in order to evaluate the need for a more precise standardization of AE reports in the literature.

## Material and methods

### Search strategy

A systematic search for records from 14 May 2020 to 25 October 2023 in PubMed Central was performed using different combinations of the following keywords: “Atezolizumab plus Bevacizumab” AND “Hepatocellular Carcinoma”, OR “Atezolizumab plus Bevacizumab” AND “HCC”, OR “Atezolizumab and Bevacizumab” AND Hepatocellular Carcinoma”, OR “Atezolizumab and Bevacizumab” AND “HCC”, OR “Atezolizumab-Bevacizumab” AND “Hepatocellular Carcinoma” AND “Adverse events”, OR “Atezolizumab-Bevacizumab” AND “HCC” AND “Adverse events”, OR “Atezolizumab-Bevacizumab” AND “Hepatocellular Carcinoma”, OR “Atezolizumab-Bevacizumab” AND “HCC”. This study was conducted in accordance with PRISMA (Preferred Reporting Items for Systematic Review and Meta-Analysis) guidelines.^12^

### Study selection and data extraction

The studies identified by the search were subsequently evaluated according to the following inclusion and exclusion criteria. Inclusion criteria: articles written in English, target population of the original paper was patients with HCC treated with atezolizumab-bevacizumab, general or specific percentages of AEs occurring during atezolizumab-bevacizumab treatment were reported. Exclusion criteria: phase Ib and phase III randomized clinical trial evaluating atezolizumab-bevacizumab, case-reports, systematic reviews and meta-analyses, sub-analyses of phase Ib and phase III trials, articles reporting AEs occurring when atezolizumab-bevacizumab was used in combination with other treatments (*e.g.* loco-regional treatments), articles reporting AEs occurring when atezolizumab-bevacizumab was used as adjuvant treatment, articles reporting AEs occurring during therapy with immune checkpoint inhibitors including atezolizumab-bevacizumab, but where the percentages of AEs for this specific combination, among all the systemic treatments, were not clearly reported.

Published systematic reviews and meta-analyses were screened to ensure that all studies reporting AEs during atezolizumab-bevacizumab were included.

Two reviewers (DP and CC) independently screened titles and abstracts of the articles extracted. The full texts of the eligible articles were then independently reviewed. Any discrepancies in article selection were resolved by discussion with a third expert reviewer (JCN). Data extraction from the included studies was then performed independently by two reviewers (DP and CC). The complete list of variables extracted from the included studies are reported in Table S1.

The outcomes of interest were the reporting of AEs and the incidences of these AEs during atezolizumab-bevacizumab treatment. In particular, we recorded general percentages of any AEs regardless of their severity, percentages of any AEs divided by severity grade according to the classification used in each study, and percentages of each specific AE regardless of grade and divided by severity grades. We recorded if frequent potential immune-related AEs, such as rash, hepatitis and thyroid disorder, were reported (and their incidence), as well as less common immune-related AEs (such as colitis, pneumonitis, nephritis, neuropathy, myositis, adrenal insufficiency, hypophysitis and/or rheumatological diseases) and the use of corticosteroids to treat immune-related AEs.

### Statistical analysis

First, we conducted a descriptive analysis to assess the number of studies reporting each AE, as well as evaluating the characteristics of the included population. For each AE reported in these studies, we evaluated the distribution of percentages by calculating the minimum, maximum, median, and interquartile range (raw data). Distinct analyses encompassing all grades of AEs and each severity grade (grade 1/2, grade 3/4, grade 5) were performed. Initially, we performed the analysis by excluding studies from the same research group to eliminate potential overlap. If multiple publications reported on the same study population, the one with the higher number of patients was included. We considered studies to be from the same research group when they were explicitly declared as such or when we identified recurring authors across different papers.

In a second step, we conducted a meta-analysis using the meta-packages and metaprop functions of R statistical software version 4.1.1. The proportion of patients experiencing each AE and the corresponding number of patients were extracted from each study along with the total number of patients included in each study. To account for between-study heterogeneity, an inverse variance random-effects model was employed, assuming a common between-study variance. The DerSimonian-Laird estimator was used to estimate the between-study variance (τˆ2), which quantifies the amount of heterogeneity among the included studies. Forest plots were generated to visualize the individual study estimates along with the overall pooled estimate, with confidence intervals representing the uncertainty around the pooled estimate.

Next, we studied the occurrence of AEs adjusted to the length of exposure to atezolizumab-bevacizumab. For studies where the median treatment duration with atezolizumab-bevacizumab was available, the outcome of interest was reported as exposure-adjusted incidence rate. Incidence rates per 100 patient-years were calculated by dividing the total number of patients experiencing each AE by the sum of all patients’ time (in 100 years) of exposure during the treatment period.^13^ Finally, we analyzed differences in reported AEs across the same cohorts, including all identified reports for each cohort. For each study, we also recorded the median overall survival (OS) and progression-free survival (PFS) when available, along with the HR and its corresponding 95% CIs, as well as the number of patients included in the survival analysis. Values of *p* <0.05 were considered significant. All the analyses were conducted in R statistical software version 4.1.1 (R: A Language and Environment for Statistical Computing, Vienna, Austria).

## Results

### Study selection and characteristics

**Fig. 1:**
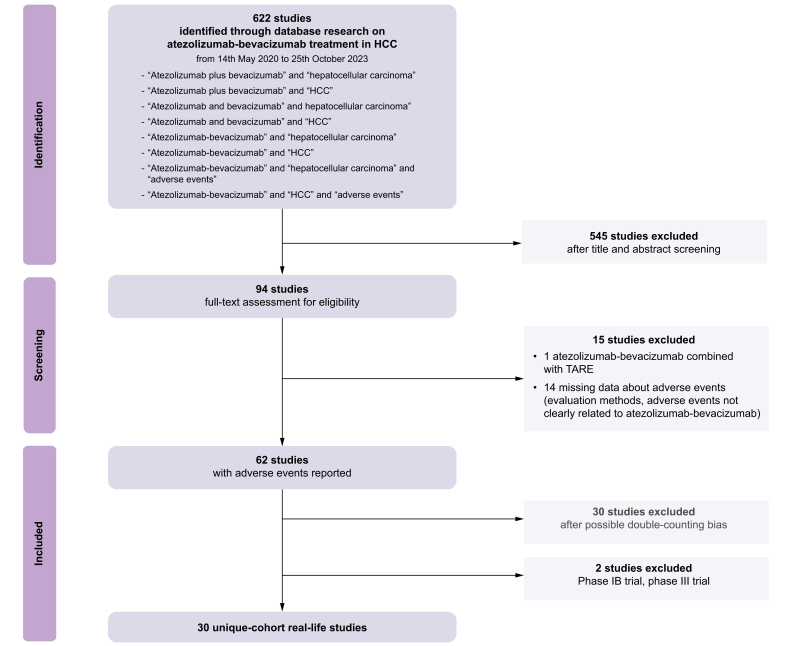
Flow chart of the study (adapted from PRISMA 2020 flow diagram). HCC, hepatocellular carcinoma; TARE, trans-arterial radioembolization.

**Fig. 2:**
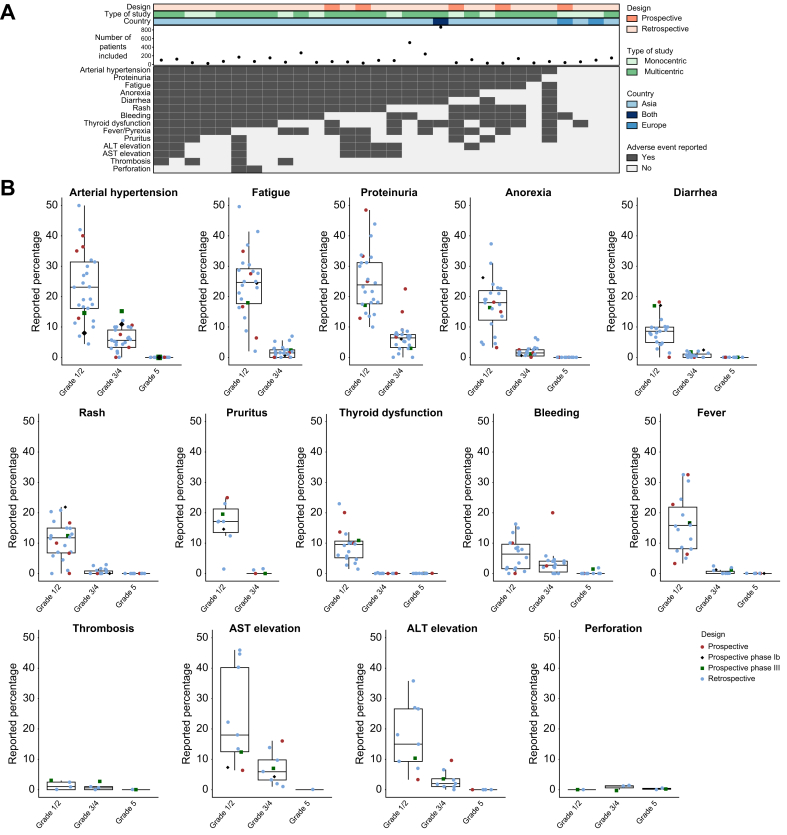
Raw data on adverse events reported across 30 studies. (A) Heatmap illustrating the presence or absence of descriptions of adverse events, regardless of their grade, across 30 included studies. Each column represents a study analyzed with its annotations. (B) Boxplots displaying the distribution of percentages of each adverse event (raw data) across studies, categorized according to their severity. Boxes represent the IQR of the data, the horizontal line the median value and whiskers the range of the data, extending to 1.5 times the IQR from the quartiles. Individual data points corresponding to each study are plotted and colored differently based on the type of study. On the graph of each adverse event, the percentages reported in the phase Ib and phase III studies have also been included for comparison; however, the data from phase Ib and phase III were not used in the calculation of the median and IQR. ALT, alanine aminotransferase; AST, aspartate aminotransferase.

Our systematic literature search initially identified 622 relevant publications. After the removal of case reports, systematic reviews and meta-analyses, 94 records remained. With the evaluation of the full-text articles, we further excluded 32 irrelevant publications including articles without clear data about AEs (n = 14) and combination therapies (n = 1). Consequently 62 eligible studies involving patients were included (Fig. 1; Table 1). Thirty studies were further excluded as they were from the same research group, suggesting potential overlap among patients, two studies were excluded as they were phase Ib and phase III randomized-controlled trials. Finally, 30 studies considering 3,867 patients were included in the qualitative analysis (Fig. 1). Most of the included studies (83%) were retrospective, whereas four studies (17%) collected data prospectively (Fig. 2A). Sixty-seven percent of the studies were multicentric, with the majority conducted in Asia (90%) (Table S2). The median age of patients was 72 years and ranged from 53.7 to 76 years. Most of the patients reflected the real-world setting of atezolizumab-bevacizumab prescription rather than the inclusion criteria of the IMbrave 150 trial, given that most studies (83%) included patients with Child-Pugh class ≥A and that 75% of the studies included patients who received atezolizumab-bevacizumab as first, second, or later lines of treatment. Temporary or definitive withdrawal of atezolizumab-bevacizumab was reported in 11 (36.6%) studies with percentages ranging from 0 to 70%, whereas permanent withdrawal was described in only nine studies (30.0%) with percentages ranging from 0 to 41%. The median OS of patients treated with atezolizumab-bevacizumab was reported in 16 (53.3%) of the 30 studies. Specifically, five of these studies indicated that the median OS had not yet been reached, while the median survival in the remaining 11 studies was 15.4 months (IQR 12-19 months). The hazard ratios (HRs) and 95% CIs were provided in only four studies. The median PFS was reported in 15 (50%) of the studies, with a median value of 7 months (IQR 5.3-8 months). HRs and their corresponding 95% CIs were reported in just two studies.

**Table 1:** Description of the studies included in the analysis (n = 30).

References	Retrospective/prospective	Monocentric/multicentric	Country	Number of patients	Primary endpoint	Median OS	Median PFS	Child-Pugh B/C (%)	HBV (%)	HCV (%)	MASLD (%)	Alcohol (%)	Male (%)	Median age (years)	Adverse events evaluation
Kuzuya *et al.*^24^	Retrospective	Monocentric	Asia	23	ORR, safety	NA	NA	0	17,4	21,7	NA	NA	78,3	74	CTCAE v5.0
Sho *et al.*^25^	Retrospective	Multicentric	Asia	58	ORR, DCR	NA	NA	7,8	25	23,4	NA	NA	85,9	72	CTCAE v4.0
Ando *et al.*^26^	Retrospective	Monocentric	Asia	40	ORR, safety	NA	NA	0	NA	NA	NA	NA	75	69	CTCAE v5.0
Hayakawa *et al.*^27^	Retrospective	Monocentric	Asia	52	ORR, safety, DCR	Not reached	4,7	7,7	19,2	38,5	NA	25	80,8	73	CTCAE v5.0
Eso *et al.*^28^	Prospective	Monocentric	Asia	40	Biomarker, ORR	NA	5,0	5	15	32,5	NA	NA	87,5	70,5	CTCAE v5.0
Chuma *et al.*^29^	Retrospective	Multicentric	Asia	94	ORR, safety	NA	NA	13,8	19,1	33	NA	NA	77,6	73	CTCAE v4.0
Yang-Cheng *et al.*^30^	Retrospective	Multicentric	Asia	35	OS, PFS, ORR, DCR	22,2	5,2	17	63	17	NA	NA	89	61	CTCAE v5.0
Wang *et al.*^31^	Retrospective	Monocentric	Asia	48	Biomarker, PFS	NA	NA	12,5	58,3	27,1	NA	NA	79,2	62	CTCAE v5.0
Maesaka *et al.*^32^	Prospective	Multicentric	Asia	66	ORR	Not reached	8,8	2,9	NA	NA	NA	NA	76,8	76	CTCAE v4.0
Teng *et al.*^33^	Retrospective	Monocentric	Asia	89	Biomarker OS	NA	NA	14,6	77,5	11,2	NA	NA	84,3	61,3	CTCAE v5.0
Tomonari *et al.*^34^	Retrospective	Multicentric	Asia	71	Biomarker, OS, PFS	NA	NA	8,4	11,3	42,2	NA	NA	81,7	71	CTCAE v5.0
Ochi *et al.*^35^	Retrospective	Multicentric	Asia	242	Biomarker, PFS, ORR, DCR	Not reached	NA	NA	NA	NA	NA	NA	79,3	NA	CTCAE v5.0
Sugimoto *et al.*^36^	Prospective	Multicentric	Asia	31	OS, PFS, ORR, safety	NA	NA	13	13	35	29	23	84	72	CTCAE v4.0
Niizeki *et al.*^37^	Retrospective	Multicentric	Asia	152	OS, PFS, ORR	Not reached	8,3	NA	13,6	39,1	NA	NA	76,4	73	CTCAE v5.0
Nakagawa *et al.*^38^	Retrospective	Multicentric	Asia	123	PFS, safety	NA	NA	7,3	19,5	30,1	25,2	NA	82,9	NA	CTCAE v5.0
Casadei-Gardini *et al.*^39^	Retrospective	Multicentric	Both	864	OS, TTP, safety	16,4	NA	7,2	23,5	31,3	6,8	NA	79,9	72	CTCAE v5.0
Charonpongsuntorn *et al.*^40^	Prospective	Multicentric	Asia	30	OS, PFS, safety, QOL	10,2	6,7	0	63,3	10	10	16,7	90	58	CTCAE v4.0
Unome *et al.*^41^	Retrospective	Multicentric	Asia	69	OS, PFS, ORR	12,5	5,4	11,6	17,4	31,9	23,2	17,4	79,7	74,4	CTCAE v5.0
Cheon *et al.*^42^	Retrospective	Multicentric	Asia	169	OS, PFS, ORR	NA	NA	17,7	66,8	6,5	NA	14,7	82,2	61	CTCAE v5.0
Zeng *et al.*^43^	Retrospective	Monocentric	Asia	30	OS, PFS, ORR, DCR	16,6	7,3	NA	93,3	3,3	NA	NA	86,7	53,7	CTCAE v4.0
Matoya *et al.*^44^	Retrospective	Multicentric	Asia	110	Biomarker OS, PFS	Not reached	NA	8,2	12,7	29,1	NA	8,2	80,9	74	NA
Kulkarni *et al.*^45^	Retrospective	Multicentric	Asia	67	OS	12,0	8,0	64,1	19,4	16,4	55,2	7,5	86,5	61	NA
Tokunaga *et al.*^46^	Retrospective	Multicentric	Asia	100	OS, TTP	21,9	NA	16	20	30	NA	35	84	NA	CTCAE v5.0
Jost-Brinkmann *et al.*^47^	Retrospective	Monocentric	Europe	100	OS, PFS, ORR, DCR, safety, TTP	NA	6,3	39	NA	NA	NA	NA	87	67	CTCAE v5.0
Takaki *et al.*^48^	Retrospective	Multicentric	Asia	268	OS, PFS, safety	15,4	8,0	25,4	10,4	31,3	NA	25,1	78,7	75	CTCAE v5.0
Fukushima *et al.*^49^	Retrospective	Multicentric	Asia	150	OS, PFS	NA	NA	12	18	32,6	NA	NA	80	72	CTCAE v5.0
Yano *et al.*^50^	Retrospective	Multicentric	Asia	136	OS, ORR, safety	10,2	4,0	4	NA	NA	NA	32	77	73,1	CTCAE v5.0
Tada *et al.*^51^	Retrospective	Multicentric	Asia	506	ORR	NA	NA	10,6	16,8	33	NA	21,9	77,8	74	CTCAE v5.0
Takada *et al.*^52^	Retrospective	Monocentric	Asia	61	Biomarker, safety	19,0	7,7	26	13	46	NA	NA	83,6	74,4	CTCAE v5.0
Larrey *et al.*^18^	Prospective	Monocentric	Europe	43	Safety	12,0	7,9	14	25.6	48.8	41.9	44.2	79.1	65	NA

CTCAE, Common Terminology Criteria for Adverse Events; DCR, disease control rate; ORR, objective response rate; OS, overall survival; PFS, progression-free survival; QOL, quality of life; TTP, time to progression. The number of patients reported were those included in the safety analysis.

### Availability of data on AEs

First, we assessed how, and which, AEs were reported across the 30 studies. Adverse events were reported using Common Terminology Criteria for Adverse Events (CTCAE) version 4 in 20.0% of studies and CTCAE version 5 in 70.0% of the studies (Table S2), while in three studies (10%) the system used to grade AEs was not reported. In less than half of the studies (40.0%), the percentage of patients developing AEs of any grade regardless of causality was reported. Moreover, only 33% of the studies reported the total percentage of grade 1/2 AEs, and 40% the total percentage of grade ≥3 AEs. The most frequently reported AE was arterial hypertension, which was reported in 86.7% of the 30 included studies (Fig. 2A). The other most frequently reported AEs were proteinuria (83.3%), fatigue (83.3%), anorexia (73.3%), diarrhea (70.0%), rash (70.0%), bleeding (66.7%), thyroid dysfunction (60.0%), and fever (53.3%), while other AEs were reported in less than 50% of the studies (Fig. 2A). The percentages of thromboembolic events were available in only four studies (13.3%), while perforation was evaluated in only two studies (6.7%).

### Incidence of frequent AEs: raw data and meta-analysis

First, we evaluated the raw data on the incidence of AEs based on the severity grade as well as their variations assessed by IQR (Fig. 2B describing the raw data). For all AEs, the dispersion of the percentages, as indicated by the higher IQR, was significantly higher for grade 1/2 compared to grade 3/4 AEs (Fig. 2B describing the raw data). Specifically, the IQR of grade 1/2 AEs was nine-fold greater for rash, six-fold greater for both diarrhea and ALT (alanine aminotransferase) elevation, and five-fold greater for fatigue, compared to grade 3/4 AEs. The availability of data on each AE, and median OS and PFS, is depicted in Fig. S1 and no significant correlation was found between the percentage of each AE and median OS or PFS (Table S3).

**Fig. 3:**
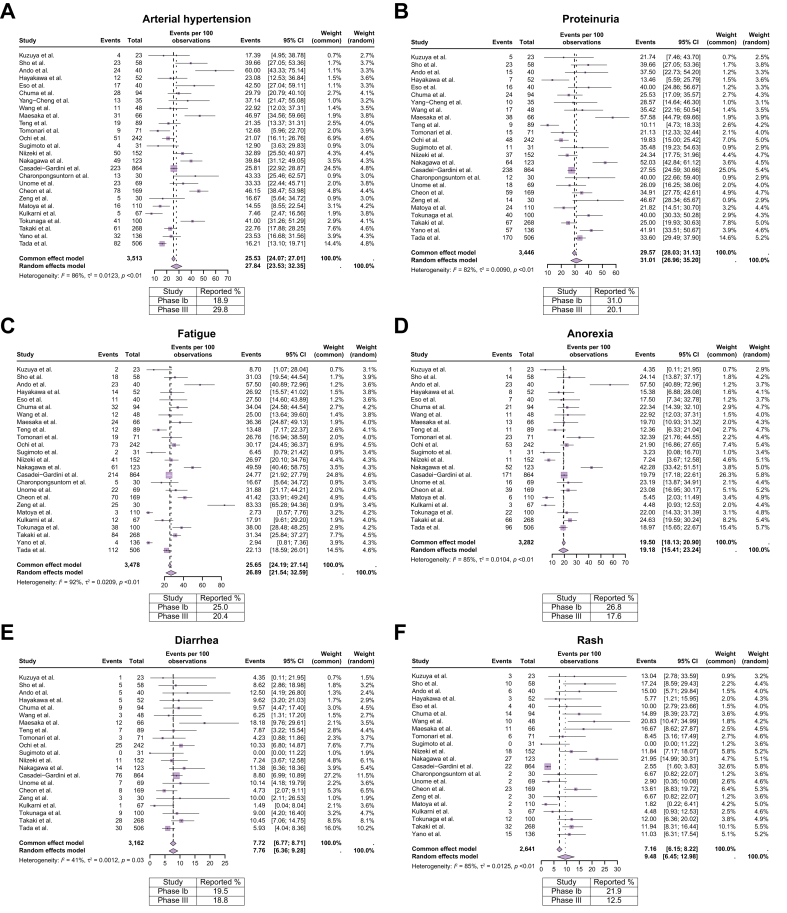
Meta-analysis for frequent adverse events occurring during atezolizumab-bevacizumab treatment. Each forest plot represents a specific adverse event category, regardless of the severity grade. (A) Arterial hypertension. (B) Proteinuria. (C) Fatigue. (D) Anorexia. (E) Diarrhea. (F) Rash. Squares indicate estimates; size of squares, study weights; whiskers, 95% CIs; diamonds, mean estimates.

**Fig. 4:**
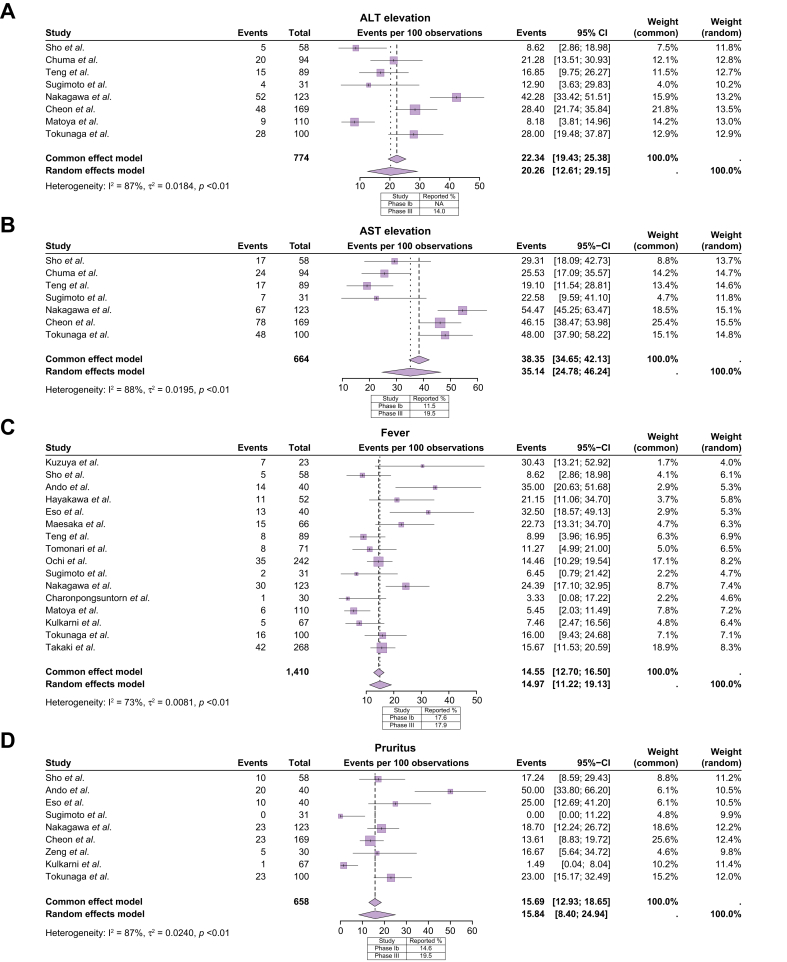
Meta-analysis for the less frequent adverse events occurring during atezolizumab-bevacizumab treatment. Each forest plot represents a specific adverse event category, regardless of the severity grade. (A) ALT elevation. (B) AST elevation. (C) Fever. (D) Pruritus. Squares indicate estimates; size of squares, study weights; whiskers, 95% CIs; diamonds, mean estimates. ALT, alanine aminotransferase; AST, aspartate aminotransferase.

Next, we performed a meta-analysis in order to accurately describe the incidence of AEs. The incidence rates of any grade, grade 1/2, and grade 3 or higher treatment-related AEs (trAEs) were 79% (95% CI 68%-89%), 56% (95% CI 38%-73%) and 30% (95% CI 20%-41%), respectively. In the IMbrave 150 trial, the incidence rates of any grade, grade 1/2, grade 3 or higher trAEs were 98.2%, 41.7% and 56.5%, respectively. Thus, we conducted a separate meta-analysis to evaluate the incidence of 14 different AEs linked to atezolizumab-bevacizumab, synthesizing data from the 30 independent studies and comparing these percentages with those described in the phase Ib and phase III studies (Fig. 3, Fig. 4). When considering all-grade trAEs, significant heterogeneity was observed among the included studies, requiring the use of random-effects models to obtain reliable pooled estimates. In contrast, for grade ≥3 AEs, the rates among studies were more consistent with heterogeneity not significant in most cases. This allows for the use of fixed-effects models to provide more precise estimates. While the pooled rates of arterial hypertension (28%), anorexia (19%), bleeding (8.9%), fever (15%), pruritus (16%), rash (9%) and thyroid dysfunction (7%) of any grade were similar to those reported in the IMbrave 150 trial; the rates of proteinuria (31%), fatigue (27%), ALT elevation (20%), AST elevation (35%) and perforation (2%) were higher, while the rates of diarrhea (8%) and thrombosis (9%) were lower (Fig. 3, Fig. 4). When focusing on grade ≥3 AEs, the percentages were consistent with those reported in the IMbrave 150 trial, except for arterial hypertension (5% in our analysis *vs*. 15.2% in IMbrave 150) and thrombosis (0% in our analysis *vs.* 2.7% in IMbrave150), which were more frequently observed in the IMbrave 150 trial.

**Fig. 5:**
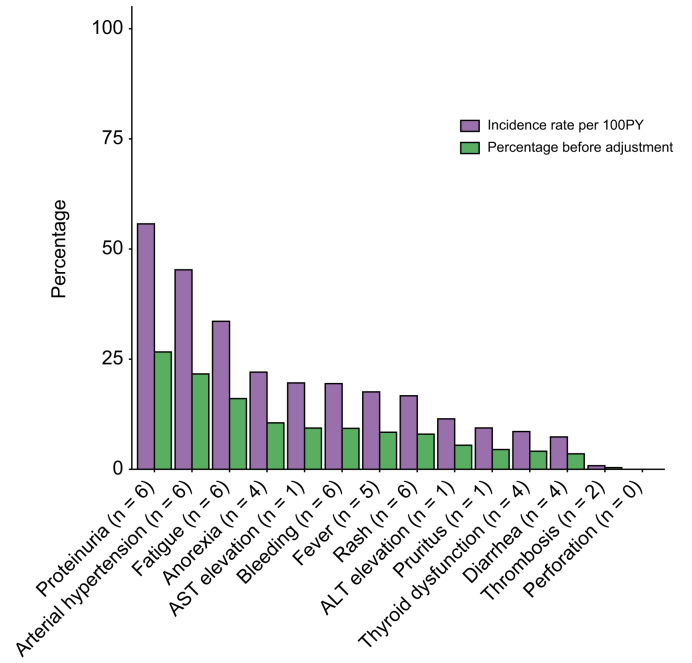
Exposure-adjusted incidence rate of adverse events in patients with HCC treated with atezolizumab-bevacizumab. The blue set of columns represents exposure-adjusted rates per 100 PY, while the yellow set represents percentages reported across the seven studies for which the median duration of treatment with atezolizumab-bevacizumab was known. ALT, alanine aminotransferase; AST, aspartate aminotransferase; PY, patient-years.

### Exposure-adjusted incidence rates of frequent AEs

Seven of the 30 studies including a total of 512 patients reported the median duration of treatment with atezolizumab-bevacizumab, which ranged from 2.8 to 9.4 months. For these studies, we calculated the exposure-adjusted incidence rate (EAIR) for each of the 14 AEs we considered (Figs 5 and S1). Our analysis revealed that the AEs of any grade with the highest EAIR were proteinuria (55.7%), arterial hypertension (45.3%), and fatigue (33.6%). Similarly, when considering grade ≥3 AEs, the highest EAIRs were observed for proteinuria (5.7%), arterial hypertension (4.5%) and bleeding (4.5%). The delta between the unadjusted incidence rate *vs.* the EAIR of AEs of any grade was 29.08% for proteinuria, 23.6% for arterial hypertension, 17.5% for fatigue, 11.5% for anorexia, 10.2% for AST elevation and 10.2% for bleeding.

### Incidence of bleeding, thrombosis, perforation and rare immune-related AEs

Furthermore, among the 21 studies that reported the occurrence of immune-related AEs (including mainly the more frequent immune-related AEs such as thyroid dysfunction, diarrhea, hepatitis and rash), no information was provided regarding the use of corticosteroids in 80.7% of the cases. Eight percent (IQR 5.6-9.0%) of patients treated with atezolizumab-bevacizumab received corticosteroids for immune-related AEs among the four studies reporting these data (Table S4). The main reason for corticosteroid therapy was predominantly related to immune-mediated hepatitis. However, the terminology and definition of this AE varied between the studies (liver dysfunction, hepatitis, liver injury, etc.) (Table S4). Only 8 (26.7%) of the studies reported the incidence of “rare” immune-mediated AEs such as colitis, pneumonitis, nephritis, neuropathy, myositis, adrenal insufficiency, hypophysitis and/or rheumatological diseases.

Regarding other severe AEs, 20 (66.7%) studies reported data about bleeding, 4 (13.8%) studies the occurrence of thromboembolic events, and only 2 (6.9%) studies the occurrence of gastrointestinal perforation (Fig. 2A).

Bleeding of any grade, regardless of type, occurred at a median frequency of 9.1% (IQR 6.0-15.5%) similar to that reported in the phase III trial (7.0%). However, bleeding was described in a highly heterogeneous manner across the studies (Table S5). Among the 20 studies reporting the percentages of bleeding, six did not report the cause of bleeding, three reported only bleeding such as epistaxis or hemoptysis (but not the incidence of gastrointestinal bleeding) and finally 11 reported the rate of gastrointestinal bleeding (including precise data on variceal bleeding in only four of the studies). Among the four studies reporting the rate of variceal bleeding, the incidence of variceal bleeding was 4.5% (IQR 1.7%-16.9%). Conversely, venous thrombosis was reported with a median frequency of 1.8% (IQR 1.1%-4.0%) which was lower compared to the incidence observed in the IMbrave trial (5.7%). In contrast, the incidence of gastrointestinal perforation was slightly higher in published studies (median 2.0%, IQR 1.8%-2.5%) compared to the incidence reported in the IMbrave trial (0.3%).

### Intra and inter-cohort variability in AE reporting

**Fig. 6:**
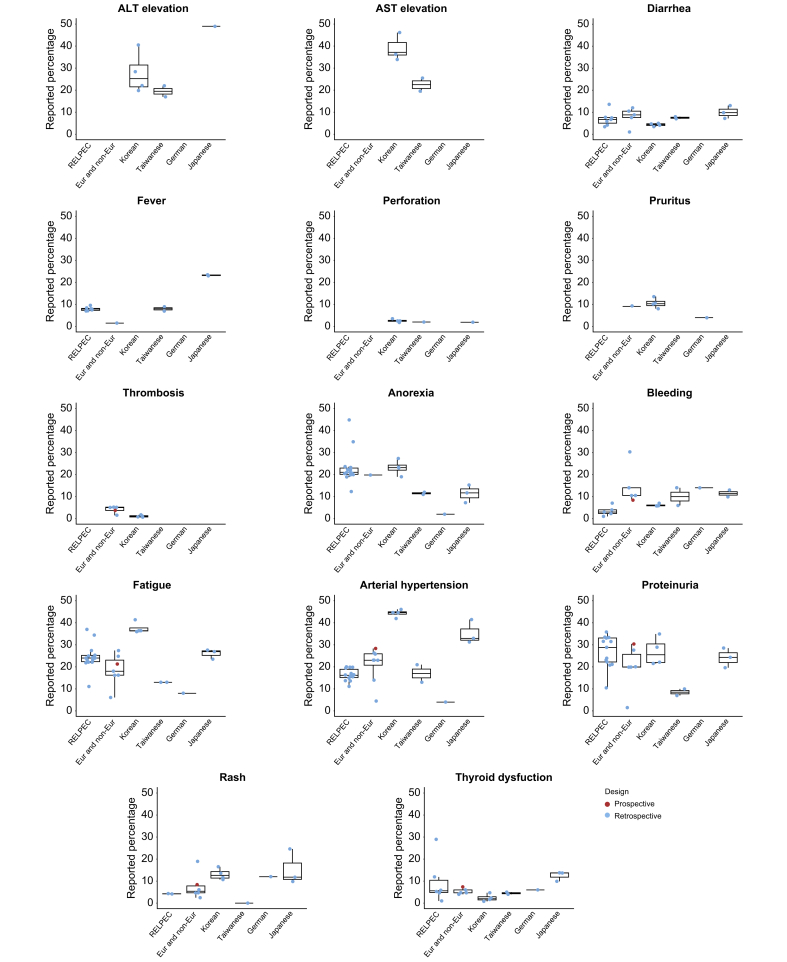
Percentage of adverse events reported in studies published by the same research group. Boxplots displayed the distribution of percentages of each any grade adverse event (raw data) reported in different studies published by the same group: RELPEC group (14 studies), European and non-European cohorts (10 studies), Korean cohorts (5 studies), Taiwanese cohorts (2 studies), German cohorts (2 studies) and Japanese cohorts not included in RELPEC group (3 studies). Boxes represent the IQR of the data, the horizontal line the median value and whiskers the range of the data, extending to 1.5 times the IQR from the quartiles. Individual data points corresponding to each study. ALT, alanine aminotransferase; AST, aspartate aminotransferase.

We evaluated the rate of 14 distinct AEs across multiple publications (n = 62) from six different cohorts, which included overlapping patient populations: the RELPEC group (14 publications, 29 to 506 patients according to the study), a collaborative group including European and non-European centers (10 publications, 65 to 864 patients according to the study), a Japanese collaborative group not included in RELPEC (3 publications, 51 to 152 patients according to the study), a German group not included in the previous European group (2 publications, 50 to 100 patients according to the study), a Taiwanese and a Korean collaborative group (2 and 5 publications respectively, 46 to 89 patients and 86 to 169 patients, respectively) (Figs 6, S2 and S3; Table S6). Among the 14 RELPEC publications, only arterial hypertension, anorexia and fatigue were reported in all the studies of the group and proteinuria in 13 out of the 14 studies (Fig. 6). While the percentages of all grades of arterial hypertension were quite similar among the same studies from the RELPEC group, ranging from 11.1% to 20%, the percentages of all-grade anorexia, fatigue and proteinuria were extremely different, ranging from 12.3% to 44.8%, 11.1% to 37.7%, and 10.5% to 35.8%, respectively (Figs 6 and S2). The second largest cohort was the one composed of European and non-European patients with 10 different articles (Figs 6 and S2). Reported AEs were heterogeneous, underlined by the fact that none of the AEs were reported across all 10 studies. The most frequently reported AEs in this group were arterial hypertension (8 out of 10 studies), fatigue (7 out of 10 studies), proteinuria (6 out of 10 studies), and rash (6 out of 10 studies). In this cohort, the percentage of arterial hypertension, fatigue, proteinuria, and rash, regardless of grade, ranged between 0-5% and 30% (Figs 6 and S2). Finally, The Korean cohort was composed of five studies and the percentage of AEs was very similar across all these studies (Figs 6 and S2).

## Discussion

AEs associated with systemic treatments are a critical concern in clinical trials and in clinical practice. To guide AE classification and grading in clinical trials, the US National Cancer Institute introduced the CTCAE and the CONSORT International Committee issued different guidelines to standardize AE data reporting.^14^^,^^15^ Despite these measures, evidence shows inadequate AE reporting in clinical trials and real-life studies, with poor adherence to these guidelines.^16^^,^^17^ Moreover, prospective *vs.* retrospective study, the use of patient-reported outcomes *vs.* clinician-reported outcomes, and the duration of follow-up influence the reported incidence and severity of AEs.

Our systematic review and meta-analysis of data from 30 studies including 3,867 patients with HCC treated with atezolizumab-bevacizumab sought to provide a comprehensive assessment of trAEs available in the literature. First, few studies have reported the percentage of temporary or permanent atezolizumab-bevacizumab discontinuation with available data in only 11 and 9 studies, respectively. Moreover, the percentage of temporary or permanent treatment suspensions ranged from 0% to 70% and permanent suspensions from 0% to 41%, suggesting a very heterogenous assessment. Our analysis reveals a significant heterogeneity across studies in terms of types and percentages of AEs reported, and criteria used for their classification. Most studies reported the frequent AEs occurring in the IMbrave 150 trial, such as arterial hypertension (89.7%), proteinuria (86.2%), and fatigue (86.2%). However, data on AST elevation, which was found in 16% of patients in IMbrave 150, was available in only 23.3% of the studies in the literature. When we calculated the pooled rates for the 14 different AEs regardless of the grade of severity based on a meta-analysis, similar results to IMbrave 150 were found for arterial hypertension, anorexia, bleeding, fever, pruritus, rash and thyroid dysfunction of any grade.^3^^,^^4^ The results of our meta-analysis indicate that, overall, no additional safety signals were identified with the use of atezolizumab-bevacizumab in clinical practice. Nevertheless, it was difficult to draw definitive conclusions for venous thrombosis, bleeding, perforation, and immune-related AEs because these complications were rarely reported in the literature. For all the 14 AEs analyzed in our systematic review, we observed that the incidence of grade 1/2 AEs was more variable across studies compared to more severe AEs. This data highlights the necessity for a more rigorous assessment of reported AEs, particularly because the grading of these AEs may be influenced by the need to initiate or modify baseline therapy, as seen in cases of thyroid dysfunction or arterial hypertension. These results underscore that physicians tend to pay greater attention and provide more accurate reporting in case of severe AEs.

The rare AEs, like bleeding, thromboembolic events and gastrointestinal perforation, were also poorly described.^3^^,^^4^ In the IMbrave150 trial, all patients were required to have an upper endoscopy screening for esophageal and gastric varices within the last 6 months and required adequate treatment of varices before inclusion.^3^^,^^4^ Despite the stringent inclusion criteria, the bleeding rate reported in the IMbrave 150 trial was 7%, warranting further investigation in real-world clinical practice. However, in most of the studies we reviewed, the occurrence of bleeding, particularly gastrointestinal bleeding, was not adequately reported. Indeed, 30% of studies which reported the percentage of bleeding did not specify the causes of bleeding, and the occurrence of acute variceal bleeding was reported in only four studies. More granular data is required to assess the risk of bleeding due to portal hypertension in these patients in clinical practice, as well as to identify specific risk factors for bleeding in this population.^18^

Moreover, immune checkpoint inhibitors can cause immune-related adverse events.^19^ Although toxicity can affect any organ, the most commonly reported irAEs involve the skin, the endocrine system, and the digestive tract including the liver.^13^ In the IMbrave 150 trial, the most frequent immune-related AE reported was immune hepatitis, defined as all incidents of diagnostic and laboratory abnormalities.^3^^,^^4^ The reporting of immune-related AEs was extremely heterogeneous across publications. Furthermore, the development of immune-related AEs is sometimes difficult to differentiate from complications of cirrhosis.^20^ Additionally, it is noteworthy that in these studies, the number of patients with cirrhosis is often not specified and the assessment of liver function not well-described, potentially resulting in interpretative bias, particularly concerning liver-related AEs and bleeding. Very few studies reported information about the necessity of corticosteroid therapy with only five studies providing details on corticosteroid use. Providing this information is essential, as these AEs require precise management, and detailed reports could enhance clinicians' knowledge and capabilities in effectively treating these AEs.

The EAIR is a crucial metric in oncology as it provides an accurate evaluation of the incidence of AEs by considering the lengths of exposure to treatment.^13^ However, among the 30 studies we evaluated, only seven reported data on median treatment duration. The EAIRs for proteinuria, arterial hypertension, and fatigue were notably high, suggesting that the onset of some AEs may manifest over time, depending on the period of exposure to the treatment, underlying the need for close monitoring and management of these AEs in clinical practice. Moreover, the correct evaluation of AE occurrence is crucial, as their presence may be variably associated with treatment response, as has already been demonstrated with sorafenib.^21^^,^^22^ Finally, we also observed variability in the reporting of 14 distinct AEs across publications from six different cohorts. The RELPEC group showed wide ranges for all-grade anorexia, fatigue, and proteinuria, while the European and non-European collaborative group also exhibited substantial variability in arterial hypertension, fatigue, proteinuria, and rash percentages.

Some limitations of this study need to be acknowledged. First, the different studies included patients with heterogeneous characteristics in terms of demographics, etiology, liver function, and treatments prior to atezolizumab-bevacizumab. Most of the endpoints of these studies were not focused on the description of AEs, probably explaining the variation in terms of reporting AEs. Furthermore, most of the studies were conducted in Japan, which may hinder the applicability of the data to Western countries.^5^^,^^23^ More studies from other regions are needed to accurately understand the real AE profile for atezolizumab-bevacizumab. In addition, we considered the AEs that emerged during the treatment with atezolizumab-bevacizumab and not throughout the entire follow-up period of the patient, which may impact the identification of late-onset AEs that appear after the treatment has been discontinued.

In conclusion, our systematic review and meta-analysis provides insights into the heterogeneity of safety profiles in patients with HCC treated with atezolizumab-bevacizumab in the real-world setting. The significant heterogeneity in AE reporting and the underreporting of severe AEs such as bleeding, thrombosis and perforation, as well as corticosteroid use for immune-related AEs, highlight areas for improvement in clinical research.

## Abbreviations

AE, adverse event; AST, aspartate aminotransferase; CTCAE, Common Terminology Criteria for Adverse Events; EAIR, exposure-adjusted incidence rate; HCC, hepatocellular carcinoma; OS, overall survival; PFS, progression-free survival; PRO, patient-reported outcomes; trAE, treatment-related adverse event.

## Financial support

The authors did not receive any financial support to produce this manuscript.

## Conflict of interest

Jean-Charles Nault received research funding from Bayer and Ipsen. Pierre Nahon has received honoraria from and/or consults for AstraZeneca, Bayer, Bristol-Myers Squibb, Eisai, Gilead, Guerbet, Ipsen, and Roche. He received research grants from AstraZeneca, AbbVie, Bristol-Myers Squibb and Eisai. Sabrina Sidali has received honoraria from AstraZeneca. NGC received travel and congress fees, Consulting fees or honoraria for lectures, presentations, speaker’s bureaus from Abbvie, Gilead, Intercept and Roche.

Please refer to the accompanying ICMJE disclosure forms for further details.

## Authors’ contributions

Contributions to conception and design: CC, DP, JCN, Acquisition of data: CC, DP, JCN, Analysis and interpretation of data: CC, DP, JCN, Drafting, revising, and the manuscript content: CC, DP, SS, OG, LB, VG, GN, AD, PN, NG, JCN, Final approval of the version to be published: CC, DP, SS, OG, LB, VG, GN, AD, PN, NG, JCN.

## Data availability statement

The datasets generated and analyzed during the current systematic review and meta-analysis are available from the corresponding author upon reasonable request.
