# Supplementary material for: Heterogeneity in adverse events related to atezolizumab-bevacizumab for hepatocellular carcinoma reported in real-world studies
Source: JHEP Rep. 2024 Aug 22;6(11):101190. doi: 10.1016/j.jhepr.2024.101190 (PMC11550199; doi:10.1016/j.jhepr.2024.101190)
Supplement: Multimedia component 1 [file mmc1.pdf]

Heterogeneity in adverse events related to atezolizumab-bevacizumab for hepatocellular carcinoma reported in real-world studies

Claudia Campani, Dimitrios Pallas, Sabrina Sidali, Olga Giouleme, Lorraine Blaise, Véronique Grando, Gisele Nkontchou, Alix Demory, Pierre Nahon, Nathalie Ganne-Carrié, Jean-Charles Nault

Table of contents

Supplementary figures 2

Supplementary tables 5

Supplementary figure legends 14

Supplementary references 15

Supplementary figures

Fig. S1

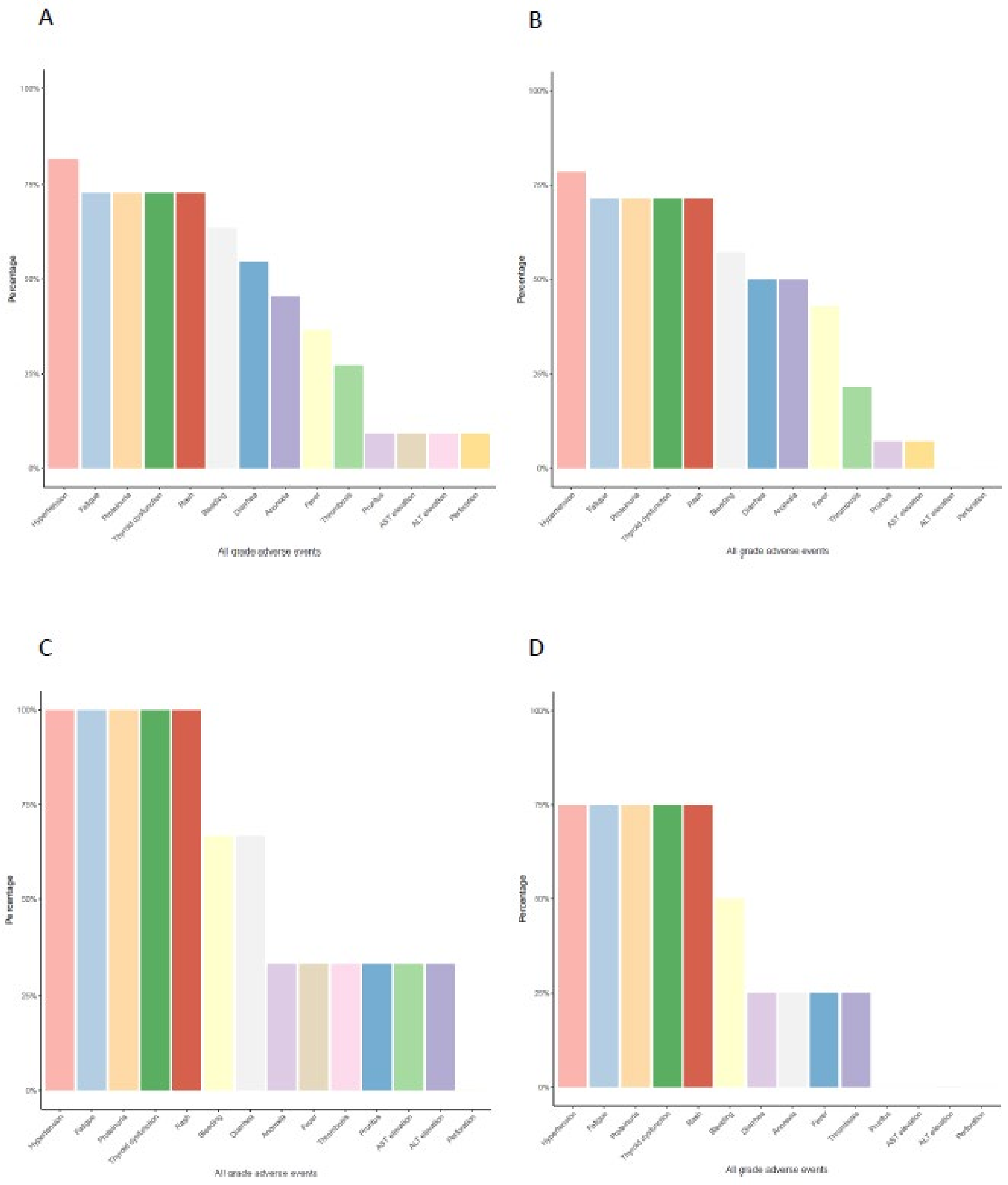

Fig. S2

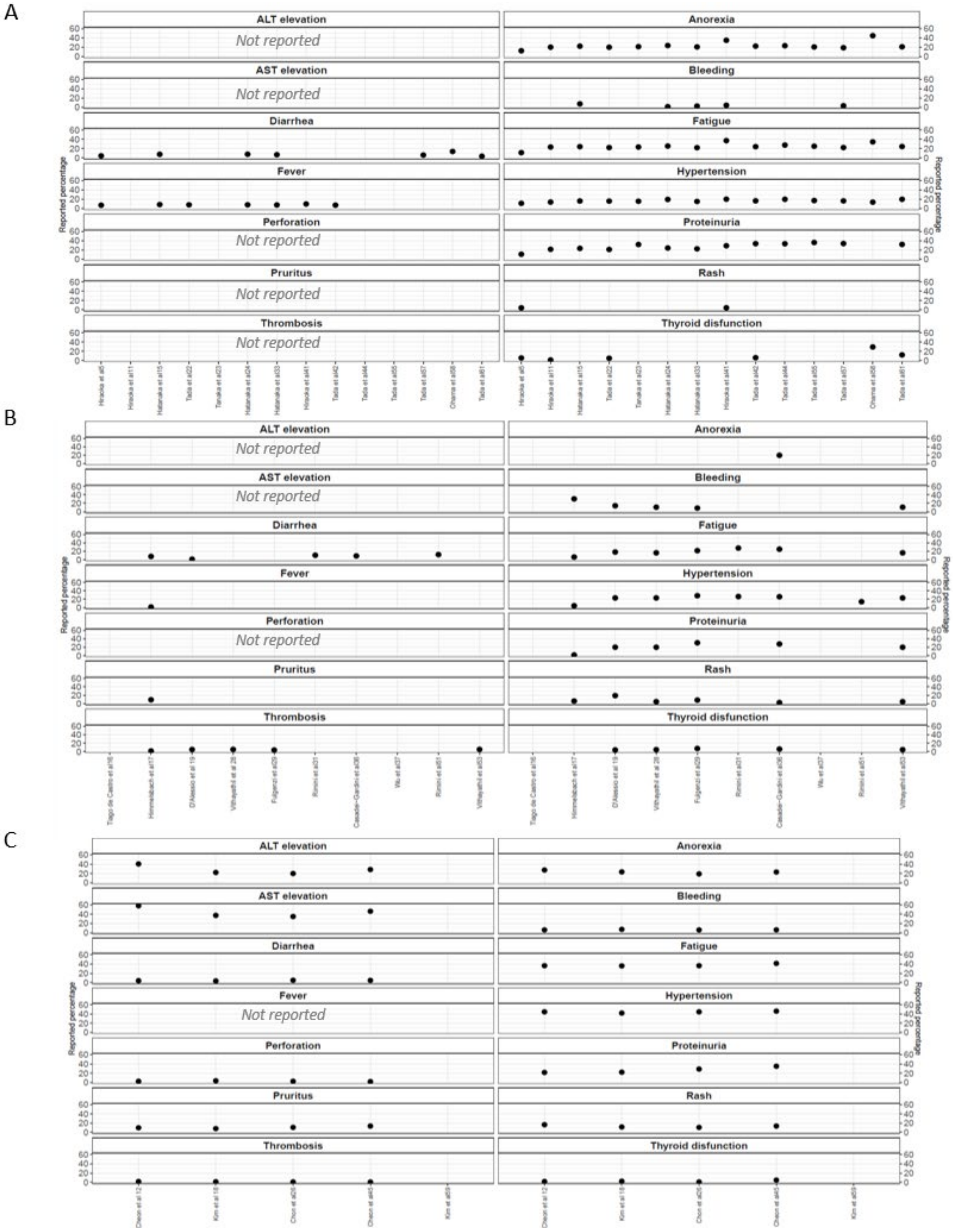
A
ALT elevation
Not reported
Anorexia
AST elevation
Not reported
Bleeding
Diarrhea
Fatigue
Fever
Hypertension
Perforation
Not reported
Proteinuria
Pruritus
Not reported
Rash
Thrombosis
Not reported
Thyroid disfunction
Reported percentage
B
ALT elevation
Not reported
Anorexia
AST elevation
Not reported
Bleeding
Diarrhea
Fatigue
Fever
Hypertension
Perforation
Not reported
Proteinuria
Pruritus
Rash
Thrombosis
Thyroid disfunction
Reported percentage
C
ALT elevation
Anorexia
AST elevation
Bleeding
Diarrhea
Fatigue
Fever
Not reported
Hypertension
Perforation
Proteinuria
Pruritus
Rash
Thrombosis
Thyroid disfunction
Reported percentage

Fig. S3

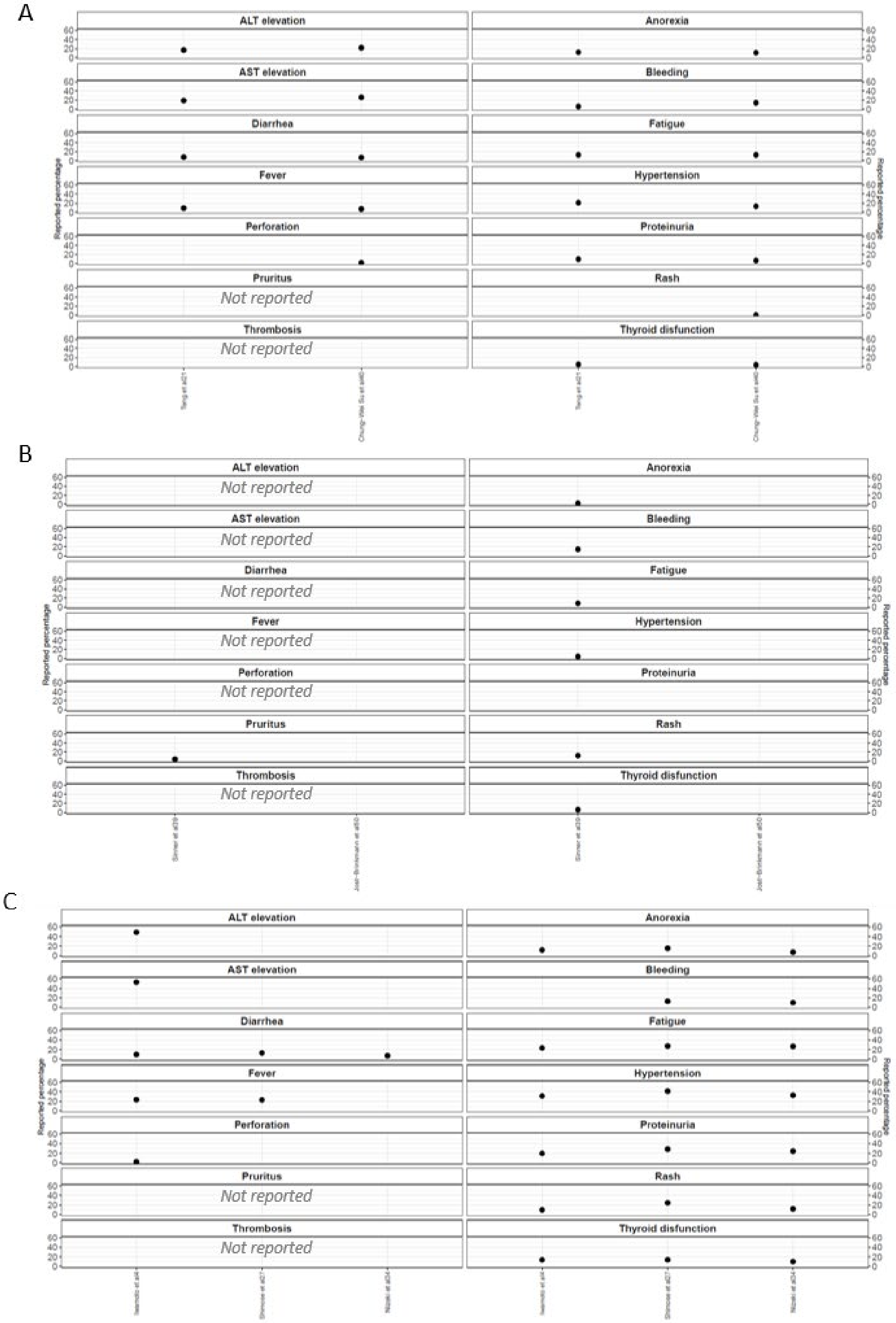
A
ALT elevation
Anorexia
AST elevation
Bleeding
Diarrhea
Fatigue
Fever
Hypertension
Perforation
Proteinuria
Pruritus
Not reported
Rash
Thrombosis
Not reported
Thyroid disfunction
Reported percentage
B
ALT elevation
Not reported
Anorexia
AST elevation
Not reported
Bleeding
Diarrhea
Not reported
Fatigue
Fever
Not reported
Hypertension
Perforation
Not reported
Proteinuria
Pruritus
Rash
Thrombosis
Not reported
Thyroid disfunction
Reported percentage
C
ALT elevation
Anorexia
AST elevation
Bleeding
Diarrhea
Fatigue
Fever
Hypertension
Perforation
Proteinuria
Pruritus
Not reported
Rash
Thrombosis
Not reported
Thyroid disfunction
Reported percentage

Supplementary tables

Table S1. Variables collected for each study

Authors
Journal
Impact factor
Title
Publication Date
Retrospective/Prospective
Monocentric/Multicentric
Asia/Europe
Cohorts
Number of patients included in safety analysis
Primary endpoint of the study
Median time of treatment with Atezo-Beva (months)
Median overall survival (months)
Median progression-free survival (months)
Number of patients included in survival analysis
Child-Pugh class A only (Yes/no)
Percentage of Child-Pugh class non-A
Atezo-Beva used as first line only
Percentage of HBV
Percentage of HCV
Percentage of metabolic syndrome
Percentage of alcohol
Percentage of male
Median Age patients Atezo-Beva
Reported adverse events (Yes/No)
Evaluation method of adverse events
Any grade all AE reported (Yes/No)
Any grade all AE (Percentage)
Grade1/2 all AE reported
Adverse events grade 1/2 all AE(Percentage)
Grade 3 all AE reported
Adverse events grade 3 all AE(Percentage)
Grade 4 all AE reported
Adverse events grade 4 all AE(Percentage)
Grade 5 all AE reported
Adverse events grade 5 all AE(death, Percentage)

Adverse events grade 5 (death causes)
Grade ≥3 all AE reported
Adverse events grade ≥3 all AE(Percentage)
Hypertension reported (Yes/No)
Hypertension (Percentage of all grade)
Hypertension (Percentage of grade 1/2)
Hypertension (Percentage of grade 3/4)
Hypertension (Percentage of grade 5)
Bleeding reported (Yes/No)
Bleeding (Percentage of all grade)
Type of Bleeding considered
Bleeding (Percentage of grade 1/2)
Bleeding (Percentage of grade 3/4)
Bleeding (Percentage of grade 5)
Thrombosis reported (Yes/No)
Thrombosis / PE (Percentage of all grade)
Thrombosis / PE (Percentage of grade 1/2)
Thrombosis /PE (Percentage of grade 3/4)
Thrombosis / PE (Percentage of grade 5)
Proteinuria reported (Yes/No)
Proteinuria Percentage of all grade
Proteinuria Percentage of (grade 1/2)
Proteinuria Percentage of (grade 3)
Fever/Pyrexia reported (Yes/No)
Fever/pyrexia (Percentage of all grade)
Fever/pyrexia (Percentage of grade 1/2)
Fever/pyrexia (Percentage of grade 3/4)
Fever/pyrexia (Percentage of grade 5)
Perforation reported (Yes/No)
Perforation (Percentage of all grade)
Perforation (Percentage of grade 1/2)
Perforation (Percentage of grade 3/4)
Perforation (Percentage of grade 5)
AST elevation reported (Yes/No)
AST elevation (Percentage of all grade)
AST elevation (Percentage of grade 1/2)
AST elevation (Percentage of grade 3/4)
AST elevation (Percentage of grade 5)
ALT elevation reported (Yes/No)
ALT elevation (Percentage of all grade)
ALT elevation (Percentage of grade 1/2)
ALT elevation (Percentage of grade 3/4)

ALT elevation (Percentage of grade 5)
Immune related other AE reported (Yes/No)
Immune related other AE (Percentage of all grade)
Type of immune related AE (free text)
Thyroid dysfunction reported (Yes/No)
Thyroid dysfunction (Percentage of all grade)
Thyroid dysfunction (Percentage of grade 1/2)
Thyroid dysfunction (Percentage of grade 3/4)
Thyroid dysfunction (Percentage of grade 5)
Diarrhea reported (Yes/No)
Diarrhea (Percentage of all grade)
Diarrhea (Percentage of grade 1/2)
Diarrhea (Percentage of grade 3/4)
Diarrhea (Percentage of grade 5)
Fatigue reported (Yes/No)
Fatigue (Percentage of all grade)
Fatigue (Percentage of grade 1/2)
Fatigue (Percentage of grade 3)
Rash/Skin toxicity reported (Yes/No)
Rash/Skin toxicity (Percentage of all grade)
Rash/ Skin toxicity (Percentage of grade 1/2)
Rash/Skin toxicity (Percentage of grade 3/4)
Rash/Skin toxicity (Percentage of grade 5)
Pruritus reported (Yes/No)
Pruritus (Percentage of all grade)
Pruritus (Percentage of grade 1/2)
Pruritus (Percentage of grade 3)
Anorexia reported (Yes/No)
Anorexia (Percentage of all grade)
Anorexia (Percentage of grade 1/2)
Anorexia (Percentage of grade 3/4)
Anorexia (Percentage of grade 5)
Other adverse events (free text)
Corticosteroids use reported (Yes/no)
Corticosteroids use reasons
Percentage of corticosteroids
AE leading to dose interruption (percentage of definitive or temporary interruption)
Percentage of AE leading to permanent withdrawal of the drug

AE: adverse event; ALT: Alanine aminotransferase; AST: aspartate aminotransferase; Atezo-Beva: atezolizumab-bevacizumab; HBV: Hepatitis B Virus: HCV: Hepatitis C Virus:

Table S2. Characteristics of the studies (n=30).

Variable	Available data	Median (IQR) or n (%)
**Design**		
Prospective	30	5 (17.0)
Retrospective		25 (83.0)
**Center**		
Monocentric	30	10 (33.0)
Multicentric		20 (67.0)
**Region**		
Asia	30	27 (90.0)
Europe		1 (3.3)
Both		2 (6.7)
**Atezolizumab-Bevacizumab only as first line (yes)**	28	7 (23.3%)
**Atezolizumab-Bevacizumab only in CP class A (yes)**	28	3 (10.0%)
**Adverse events classification used**		
CTCAE v.4	27	6 (20.0)
CTCAE v.5		21 (70.0)
**Patients with cirrhosis**	4	89.00 (71.88-100.00)
**Patients with chronic hepatitis B**	25	19.20 (15.00-25.60)
**Patients with chronic hepatitis C**	25	31.30 (21.70-33.00)
**Patients with metabolic syndrome**	7	25.20 (16.60-26.83)
**Patients with chronic alcohol consumption**	12	22.45 (16.20-26.83)
**Male patients**	30	80.85 (78.80-84.22)
**Age of patients**	27	72 (63.50-73.55)

CP: Child-Pugh. CTCAE: Common Terminology Criteria for Adverse Events. HBV: Hepatitis B Virus. HCV: Hepatitis C Virus.
The two phase Ib and phase III studies were excluded from the calculations of medians (IQR) and numbers (percentages).
Median and IQR are used for descriptive analysis.

Table S3. Association among percentage of each specific adverse event of any grade and prognosis

	Raw data				Exposed Adjusted Incidence Rate			
	Overall Survival (n=11)		Progression-Free Survival (n=15)		Overall Survival (n=3)		Progression-Free Survival (n=4)	
	Coefficient correlation*	p-value	Coefficient correlation*	p-value	Coefficient correlation*	p-value	Coefficient correlation*	p-value
**Hypertension**	0.323	0.43	0.165	0.65	0.406	0.73	0.726	0.48
**Fatigue**	0.594	0.16	0.361	0.34	0.922	0.25	0.985	0.11
**Proteinuria**	-0.137	0.77	0.426	0.25	-0.500	0.67	-0.602	0.59
**Thyroid dysfunction**	-0.105	0.84	0.090	0.85	-0.655	0.55	0.143	0.91
**Rash**	0.422	0.34	0.292	0.44	0.661	0.54	-0.220	0.86
**Bleeding**	0.275	0.55	0.141	0.72	-	-	-	-
**Diarrhea**	0.407	0.50	0.190	0.72	-	-	-	-
**Anorexia**	0.434	0.56	-0.411	0.42	-	-	-	-

*Partial correlation between mOS and adverse events (for which we have at least 50% of data available) controlling for the effect of number of patients of survival analysis.

Table S4. List of the 30 Studies included in the analysis

Author	Journal	Retrospective/ Prospective	Monocentric/ Multicentric	Country	Number of patients	Corticosteroid prescription	Corticosteroid prescription in all population included	Liver-related adverse event reported	Liver-related adverse event terminology
Kuzuya et al (1)	Cancer Diagnosis & Prognosis	Retrospective	Monocentric	Asia	23	NA	NA	Yes	Cholangitis
Sho et al (2)	Hepatology Research	Retrospective	Multicentric	Asia	58	No	NA	Yes	AST elevation, ALT elevation, GGT increase, blood bilirubine increase
Ando et al (3)	Cancers (MDPI)	Retrospective	Monocentric	Asia	40	Yes	7.50%	Yes	Liver dysfunction
Hayakawa et al (4)	Investigational New Drugs	Retrospective	Monocentric	Asia	52	Yes	7.70%	Yes	Transaminases increase
Eso et al (5)	Current Oncology	Prospective	Monocentric	Asia	40	NA	NA	No	NA
Chuma et al (6)	Hepatology Research	Retrospective	Multicentric	Asia	94	NA	NA	Yes	AST elevation, ALT elevation
Yang-Cheng et al (7)	In Vivo	Retrospective	Multicentric	Asia	35	NA	NA	No	NA
Wang et al (8)	Cancers (MDPI)	Retrospective	Monocentric	Asia	48	NA	NA	Yes	Aspartate/Alanine aminotransferase increase
Maesaka et al (9)	Hepatology Research	Prospective	Multicentric	Asia	66	NA	NA	Yes	Increased AST or ALT
Teng et al (10)	American Journal of Cancer Research	Retrospective	Monocentric	Asia	89	NA	NA	Yes	AST elevation, ALT elevation
Tomonari et al (11)	Cancer Medicine	Retrospective	Multicentric	Asia	71	NA	NA	Yes	Increased transaminase
Ochi et al (12)	Hepatology Research	Retrospective	Multicentric	Asia	242	NA	NA	Yes	Liver injury
Sugimoto et al (13)	Medicine	Prospective	Multicentric	Asia	31	Yes	NA	Yes	AST elevation, ALT elevation, Blood bilirubin increase, HCC rupture
Niizeki et al (14)	Targeted Oncology	Retrospective	Multicentric	Asia	152	NA	NA	Yes	Liver disorder
Nakagawa et al (15)	Cancer	Retrospective	Multicentric	Asia	123	NA	NA	Yes	AST elevation, ALT elevation, Blood bilirubin increase
Casadei-Gardini et al (16)	European Journal of Cancer	Retrospective	Multicentric	Both	864	NA	NA	No	NA
Charonpongsuntorn et al (17)	JCO Global Oncology	Prospective	Multicentric	Asia	30	NA	NA	Yes	Aspartate aminotransferase/alanine aminotransferase elevation
Unome et al (18)	Cancers (MDPI)	Retrospective	Multicentric	Asia	69	NA	NA	Yes	Liver dysfunction
Cheon et al (19)	Therapeutic Advances in Medical Oncology	Retrospective	Multicentric	Asia	169	NA	NA	Yes	AST elevation, ALT elevation, Hyperbilirubinemia
Zeng et al (20)	Frontiers in Immunology	Retrospective	Monocentric	Asia	30	NA	NA	Yes	Increased ALT or AST, Increased blood bilirubin
Matoya et al (21)	Hepatology Research	Retrospective	Multicentric	Asia	110	NA	NA	Yes	ALT elevation
Kulkarni et al (22)	Journal of Cilinical and Experimental Hepatology	Retrospective	Multicentric	Asia	67	NA	NA	Yes	Rise in AST/ALT, rise in bilirubin >3 mg/dl
Tokunaga et al (23)	Cancers (MDPI)	Retrospective	Multicentric	Asia	100	NA	NA	Yes	AST elevation, ALT elevation, hepatitis
Jost-Brinkmann et al (24)	AP&T Alimentary Pharmacology & Therapeutics	Retrospective	Monocentric	Europe	100	NA	NA	No	NA
Takaki et al (25)	Investigational New Drugs	Retrospective	Multicentric	Asia	268	NA	NA	Yes	Increased AST or AL, increased bilirubin level
Fukushima et al (26)	The Oncolongist	Retrospective	Multicentric	Asia	150	Yes	14.60%	Yes	Liver injury, Hepatic irAE
Yano et al (27)	Journal of Gastroenterology and Hepatology	Retrospective	Multicentric	Asia	136	NA	NA	Yes	Liver dysfunction
Tada et al (28)	Journal of Gastroenterology	Retrospective	Multicentric	Asia	506	NA	NA	Yes	Hepatic examination abnormality
Takada et al (29)	Hepatology Research	Retrospective	Monocentric	Asia	61	Yes	13.10%	Yes	Liver injury
Larrey et al (30)	Liver International	Prospective	Monocentric	Europe	43	NA	NA	NA	NA

CTCAE: Common Terminology Criteria for Adverse Events. HBV: Hepatitis B Virus. HCV: Hepatitis C Virus. DCR: Disease Control Rate. ORR: Objective Response Rate. OS: Overall Survival. PFS: Progression-Free Survival. QOL: Quality Of Life. TTP: Time To Progression.

Table S5. Description of the bleeding events across the 30 studies

Variable	N (%)
Bleeding event reported	20 (66.7)
Type of bleeding*	
Not specified	6 (30.0)
Extra-GI^	3 (15.0)
GI	2 (10.0)
Extra-GI and GI	2 (10.0)
Extra-GI and variceal	1 (5.0)
Variceal and GI	2 (10.0)
Variceal	1 (5.0)

* The percentages are calculated based on the total number of studies that reported bleeding (n=20).
Extra-GI: the bleeding described in the study occurred outside the gastrointestinal system (epistaxis, bronchopulmonary). GI: gastrointestinal tract bleeding without any details regarding potential variceal bleeding. Variceal: studies that reported considering variceal bleeding among the types of bleeding.

Table S6. Characteristics of the studies included in the analysis of intra- and inter-cohort variability (n=36)

References	Journal	Retrospective/ Prospective	Monocentric/ Multicentric	Country	Number of patients	Primary endpoint	Median OS	Median PFS	Child Pugh B/C	HBV (%)	HCV (%)	Metabolic (%)	Alcohol (%)	Male (%)	Median age (years)	Adverse events evaluation method
Iwamoto et al[%] (31)	Cancers (MDPI)	Retrospective	Multicentric	Asia	51	ORR, PFS, DCR	Not reached	5,4	7,8	13,7	37,2	NA	NA	88,2	71	CTCAE v5.0
Hiraoka et al[$] (32)	Cancer Reports	Retrospective	Multicentric	Asia	171	ORR	NA	NA	4,1	15,7	35	NA	18,1	84,2	73	CTCAE v4.0
Hiraoka et al[$] (33)	Hepatology Research	Retrospective	Multicentric	Asia	95	ORR	NA	8,0	0	12,6	26,3	NA	21	76,8	76	CTCAE v5.0
Cheon et al[^] (34)	Liver International	Retrospective	Multicentric	Asia	121	OS, PFS, ORR	Not reached	6,5	0	76,9	5	NA	NA	83,5	61	CTCAE v5.0
Hatanaka et al[$](35)	Hepatology Research	Retrospective	Multicentric	Asia	239	OS, PFS, ORR	NA	NA	0	18	30,1	NA	19,2	81,6	73	CTCAE v5.0
Tiago de Castro et al*(36)	Therapeutic Advances in Medical Oncology	Retrospective	Multicentric	Europe	147	ORR, DCR	NA	NA	27,9	8,2	25,9	23,1	26,5	85	68,7	CTCAE v5.0
Himmelsbach et al* (37)	Cancers (MDPI)	Retrospective	Multicentric	Europe	66	OS, PFS, ORR, Safety	NA	NA	42,4	13,6	21,2	27,3	37,9	81,8	65	CTCAE v4.0
Kim et al[^](38)	Cancers (MDPI)	Retrospective	Multicentric	Asia	86	OS, PFS, ORR	Not reached	5,7	4,7	72,1	2,5	NA	12,8	81,4	62	CTCAE v5.0
D'Alessio et al* (39)	Hepatology	Retrospective	Multicentric	Asia	202	OS, PFS, ORR, DCR, Safety	14,9	6,8	24	17	36	11,3	19,3	85	69	CTCAE v5.0
Teng et al[#](10)	American Journal of Cancer Research	Retrospective	Monocentric	Asia	89	Biomarker OS	NA	NA	14,6	77,5	11,2	NA	NA	84,3	61,3	CTCAE v5.0
Tada et al[$](40)	Cancer Medicine	Retrospective	Multicentric	Asia	317	OS, PFS, Safety	NA	NA	5,6	17,3	33,1	NA	NA	81,4	74	CTCAE v5.0
Tanaka et al[$] (41)	Hepatology Research	Retrospective	Multicentric	Asia	427	ORR	NA	NA	6,5	17,3	34,1	NA	17,9	80,5	74	CTCAE v5.0
Hatanaka et al[$](35)	Hepatology International	Retrospective	Multicentric	Asia	297	Biomarker, OS, PFS	NA	NA	6,1	16,8	33,3	20,2	19,2	81,8	73	CTCAE v5.0
Chon et al[^](42)	Cancer Medicine	Retrospective	Multicentric	Asia	121	Biomarker, OS, PFS	Not reached	5,7	10	60,6	5,8	NA	15,7	82,6	63	CTCAE v5.0
Shimose et al[%](43)	Cancers (MDPI)	Retrospective	Multicentric	Asia	130	OS, Safety	18,2	6,4	NA	14,6	46,1	16,1	23	78,4	72,5	CTCAE v5.0
Vithayathil et al*(44)	Liver International	Retrospective	Multicentric	Asia	191	OS, PFS, ORR, DCR	NA	NA	23	19,4	37,7	13,1	38,2	84,3	68,4	CTCAE v5.0
Fulgenzi et al*(45)	European Journal of Cancer	Prospective	Multicentric	Both	296	OS, PFS, ORR	15,7	6,9	0	40,6	25,3	NA	NA	82,7	66	CTCAE v5.0
Jost-Brinkmann et al[~](24)	AP&T Alimentary Pharmacology & Therapeutics	Retrospective	Monocentric	Europe	100	OS, PFS, ORR, DCR, Safety, TTP	NA	6,3	39	NA	NA	NA	NA	87	67	CTCAE v5.0
Rimini et al*(46)	ESMO Journal	Retrospective	Multicentric	Both	190	OS	12,1	5,5	5,8	NA	NA	43,1	NA	78,4	NA	CTCAE v5.0
Hatanaka et al[$](47)	Cancer Medicine	Retrospective	Multicentric	Asia	252	PFS, ORR, DCR, Safety	Not reached	NA	0	19,2	31,9	17,3	18,3	80,2	73	CTCAE v5.0
Niizeki et al[%](14)	Targeted Oncology	Retrospective	Multicentric	Asia	152	OS, PFS, ORR	Not reached	8,3	NA	13,6	39,1	NA	NA	76,4	73	CTCAE v5.0
Casadei-Gardini et al*(16)	European Journal of Cancer	Retrospective	Multicentric	Both	864	OS, TTP, Safety	16,4	NA	7,2	23,5	31,3	6,8	NA	79,9	72	CTCAE v5.0
Wu et al*(48)	Cancers (MDPI)	Retrospective	Multicentric	Both	296	Biomarker, OS, PFS	NA	NA	0	NA	NA	NA	NA	82,7	66	CTCAE v5.0
Sinner et al (49)[~]	Cancers (MDPI)	Retrospective	Multicentric	Europe	50	OS, PFS, ORR, DCR	16,0	7,1	27	12	21	23	25	82	65	CTCAE v5.0
Chung-Wei Su et al[#](50)	Cancer Medicine	Retrospective	Monocentric	Asia	46	OS, PFS, ORR	Not reached	5,3	13	NA	NA	NA	NA	82,6	61,2	CTCAE v5.0
Hiraoka et al[$](51)	Liver Cancer	Retrospective	Multicentric	Asia	229	Safety	NA	NA	7,4	14,4	35,4	NA	17,4	81,2	74	CTCAE v5.0
Tada et al[$](52)	Cancer Medicine	Retrospective	Multicentric	Asia	421	Biomarker, OS, PFS	17,8	6.7	7,4	16,1	34,2	NA	NA	80,8	74	CTCAE v5.0
Tada et al[$](53)	Cancer Medicine	Retrospective	Multicentric	Asia	263	OS, PFS, Safety	Not reached	7,1	7,6	15,6	35,7	NA	NA	79,1	74	CTCAE v5.0
Cheon et al[^](19)	Therapeutic Advances in Medical Oncology	Retrospective	Multicentric	Asia	169	OS, PFS, ORR	NA	NA	17,7	66,8	6,5	NA	14,7	82,2	61	CTCAE v5.0
Rimini et al*(54)	Journal of Cancer Research and Clinical Oncology	Retrospective	Multicentric	Both	65	OS, PFS	8,2	6,9	100	NA	NA	32	NA	89	NA	CTCAE v5.0
Vithayathil et al*(55)	Hepatology International	Retrospective	Multicentric	Both	191	OS, PFS, ORR, DCR	14,9	6,7	23	19,4	37,7	13,1	38,2	84,3	68,4	CTCAE v5.0
Tada et al[$](28)	Journal of Gastroenterology and Hepatology	Retrospective	Multicentric	Asia	430	OS, PFS	20	7.0	0	19	33,2	NA	NA	81,1	74	CTCAE v5.0
Tada et al[$](56)	Journal of Gastroenterology	Retrospective	Multicentric	Asia	506 (EGV group = 151, non-EGV group = 355)	ORR	NA	NA	10,6	16,8	33	NA	21,9	77,8	74	CTCAE v5.0
Ohama et al[$](57)	Oncology	Retrospective	Multicentric	Asia	29	OS, PFS	5,7	5,0	100	NA	NA	NA	NA	79,3	72	CTCAE v5.0
Kim et al[^](38)	Cancers (MDPI)	Retrospective	Multicentric	Asia	114	OS, PFS	5,8	4,3	7	64,04	3,51	NA	17,54	86,8	63,3	NA

Tada et al[$](58)	Liver International	Retrospective	Multicentric	Asia	177	PFS	24,0	10,8	7,9	10,2	33,3	NA	NA	75,7	74	CTCAE v5.0

CTCAE: Common Terminology Criteria for Adverse Events. HBV: Hepatitis B Virus. HCV: Hepatitis C Virus. DCR: Disease Control Rate. ORR: Objective Response Rate. OS: Overall Survival. PFS: Progression-Free Survival. QOL: Quality Of Life. TTP: Time To Progression.

*[$]RELPEC. [%] Japan (not RELPEC). [^] Korea *Collaborative European and non-European centers. [#]Taiwain. ~ German (not included in the collaborative European and non-European group).*

Supplementary figure legends

Fig. S1. Percentage of studies reporting data on frequency of each adverse event of any grade and prognosis

Each bar illustrates the percentage of studies reporting both data of each specific adverse event of any grade and (A) median overall survival (n=11) or (B) median progression free survival (n=15). Each bar in (C) and (D) depicts the percentage of studies for which we were able to calculate the Exposed Adjusted Incidence Rate and that also reported median overall survival (n=3) and median progression-free survival (n=4), respectively.

Fig. S2. Percentage of adverse events reported in studies published by the same research group (5 studies or more per research group)

Each scatterplot illustrates the percentage of a specific adverse event (raw data) reported in studies published by the same research group, regardless of the severity grade. (A) RELPEC group (14 studies) (B) European and non-European cohorts (10 studies) (C) Korean cohorts (5 studies).

Fig. S3. Percentage of adverse events reported in studies published by the same research group (less than 5 studies per research group)

Each scatterplot illustrates the percentage of a specific adverse event (raw data) reported in studies published by the same research group, regardless of the severity grade (A) Taiwanese cohorts (2 studies) (B) German cohorts (2 studies) (C) Japanese cohorts not included in RELPEC group (3 studies).

Supplementary references

1. Kuzuya T, Kawabe N, Hashimoto S, et al. Initial Experience of Atezolizumab Plus Bevacizumab for Advanced Hepatocellular Carcinoma in Clinical Practice. CDP. 2021 May 3;1(2):83–8.

2. Sho T, Suda G, Ogawa K, et al. Early response and safety of atezolizumab plus bevacizumab for unresectable hepatocellular carcinoma in patients who do not meet IMbrave150 eligibility criteria. Hepatology Research. 2021 Sep;51(9):979–89.

3. Ando Y, Kawaoka T, Kosaka M, et al. Early Tumor Response and Safety of Atezolizumab Plus Bevacizumab for Patients with Unresectable Hepatocellular Carcinoma in Real-World Practice. Cancers. 2021 Aug 5;13(16):3958.

4. Hayakawa Y, Tsuchiya K, Kurosaki M, et al. Early experience of atezolizumab plus bevacizumab therapy in Japanese patients with unresectable hepatocellular carcinoma in real-world practice. Invest New Drugs. 2022 Apr;40(2):392–402.

5. Eso Y, Takeda H, Taura K, et al. Pretreatment Neutrophil-to-Lymphocyte Ratio as a Predictive Marker of Response to Atezolizumab Plus Bevacizumab for Hepatocellular Carcinoma. Current Oncology. 2021 Oct 14;28(5):4157–66.

6. Chuma M, Uojima H, Hattori N, et al. Safety and efficacy of atezolizumab plus bevacizumab in patients with unresectable hepatocellular carcinoma in early clinical practice: A multicenter analysis. Hepatology Research. 2022 Mar;52(3):269–80.

7. Lee YC, Huang WT, Lee MY, et al. Bevacizumab and Atezolizumab for Unresectable Hepatocellular Carcinoma: Real-world Data in Taiwan-Tainan Medical Oncology Group H01 Trial. In Vivo. 2023;37(1):454–60.

8. Wang JH, Chen YY, Kee KM, et al. The Prognostic Value of Neutrophil-to-Lymphocyte Ratio and Platelet-to-Lymphocyte Ratio in Patients with Hepatocellular Carcinoma Receiving Atezolizumab Plus Bevacizumab. Cancers. 2022 Jan 11;14(2):343.

9. Maesaka K, Sakamori R, Yamada R, et al. Comparison of atezolizumab plus bevacizumab and lenvatinib in terms of efficacy and safety as primary systemic chemotherapy for hepatocellular carcinoma. Hepatology Research. 2022 Jul;52(7):630–40.

10. Teng W, Lin CC, Su CW, et al. Combination of CRAFITY score with Alpha-fetoprotein response predicts a favorable outcome of atezolizumab plus bevacizumab for unresectable hepatocellular carcinoma. Am J Cancer Res. 2022;12(4):1899–911.

11. Tomonari T, Tani J, Sato Y, et al. Initial therapeutic results of atezolizumab plus bevacizumab for unresectable advanced hepatocellular carcinoma and the importance of hepatic functional reserve. Cancer Medicine. 2023 Feb;12(3):2646–57.

12. Ochi H, Kurosaki M, Joko K, et al. Usefulness of neutrophil-to-lymphocyte ratio in predicting progression and survival outcomes after atezolizumab–bevacizumab treatment for hepatocellular carcinoma. Hepatology Research. 2023 Jan;53(1):61–71.

13. Sugimoto R, Satoh T, Ueda A, et al. Atezolizumab plus bevacizumab treatment for unresectable hepatocellular carcinoma progressing after molecular targeted therapy: A multicenter prospective observational study. Medicine. 2022 Oct 7;101(40):e30871.

14. Niizeki T, Tokunaga T, Takami Y, et al. Comparison of Efficacy and Safety of Atezolizumab Plus Bevacizumab and Lenvatinib as First-Line Therapy for Unresectable Hepatocellular Carcinoma: A Propensity Score Matching Analysis. Targ Oncol. 2022 Nov;17(6):643–53.

15. Nakagawa M, Inoue M, Ogasawara S, et al. Clinical effects and emerging issues of atezolizumab plus bevacizumab in patients with advanced hepatocellular carcinoma from Japanese real-world practice. Cancer. 2023 Feb 15;129(4):590–9.

16. Casadei-Gardini A, Rimini M, Tada T, et al. Atezolizumab plus bevacizumab versus lenvatinib for unresectable hepatocellular carcinoma: a large real-life worldwide population. European Journal of Cancer. 2023 Feb;180:9–20.

17. Charonpongsuntorn C, Tanasanvimon S, Korphaisarn K, et al. Efficacy, Safety, and Patient-Reported Outcomes of Atezolizumab Plus Bevacizumab for Unresectable Hepatocellular Carcinoma in Thailand: A Multicenter Prospective Study. JCO Global Oncology. 2022 Dec;(8):e2200205.

18. Unome S, Imai K, Takai K, et al. Changes in ALBI Score and PIVKA-II within Three Months after Commencing Atezolizumab Plus Bevacizumab Treatment Affect Overall Survival in Patients with Unresectable Hepatocellular Carcinoma. Cancers. 2022 Dec 10;14(24):6089.

19. Cheon J, Kim H, Kim HS, et al. Atezolizumab plus bevacizumab in patients with child–Pugh B advanced hepatocellular carcinoma. Ther Adv Med Oncol. 2023 Jan;15:175883592211485.

20. Zeng H, Xu Q, Wang J, et al. The effect of anti-PD-1/PD-L1 antibodies combined with VEGF receptor tyrosine kinase inhibitors versus bevacizumab in unresectable hepatocellular carcinoma. Front Immunol. 2023 Jan 23;14:1073133.

21. Matoya S, Suzuki T, Matsuura K, et al. The neutrophil-to-lymphocyte ratio at the start of the second course during atezolizumab plus bevacizumab therapy predicts therapeutic efficacy in patients with advanced hepatocellular carcinoma: A multicenter analysis. Hepatology Research. 2023 Jun;53(6):511–21.

22. Kulkarni AV, Krishna V, Kumar K, et al. Safety and Efficacy of Atezolizumab-Bevacizumab in Real World: The First Indian Experience. Journal of Clinical and Experimental Hepatology. 2023 Jul;13(4):618–23.

23. Tokunaga T, Tateyama M, Kondo Y, et al. Therapeutic Modifications without Discontinuation of Atezolizumab Plus Bevacizumab Therapy Are Associated with Favorable Overall Survival and Time to Progression in Patients with Unresectable Hepatocellular Carcinoma. Cancers. 2023 Mar 2;15(5):1568.

24. Jost-Brinkmann F, Demir M, Wree A, et al. Atezolizumab plus bevacizumab in unresectable hepatocellular carcinoma: Results from a German real-world cohort. Aliment Pharmacol Ther. 2023 Jun;57(11):1313–25.

25. Takaki S, Kurosaki M, Mori N, et al. Effects on survival of the adverse event of atezolizumab plus bevacizumab for hepatocellular carcinoma: a multicenter study

by the Japan Red Cross Liver Study Group. Invest New Drugs. 2023 Apr;41(2):340–9.

26. Fukushima T, Morimoto M, Kobayashi S, et al. Association Between Immune-Related Adverse Events and Survival in Patients with Hepatocellular Carcinoma Treated With Atezolizumab Plus Bevacizumab. The Oncologist. 2023 Jul 5;28(7):e526–33.

27. Yano Y, Yamamoto A, Mimura T, et al. Factors associated with the response to atezolizumab/bevacizumab combination therapy for hepatocellular carcinoma. JGH Open. 2023 Jul;7(7):476–81.

28. Tada T, Kumada T, Hiraoka A, et al. Impact of first-line systemic therapy with atezolizumab plus bevacizumab in patients with hepatocellular carcinoma. J of Gastro and Hepatol. 2023 Aug;38(8):1389–97.

29. Takada H, Yamashita K, Osawa L, et al. Significance of the autoantibody assay in predicting the development of immune-related adverse events in patients receiving atezolizumab plus bevacizumab combination therapy for unresectable hepatocellular carcinoma. Hepatology Research. 2024 Feb;54(2):162–73.

30. Larrey E, Campion B, Evain M, et al. A history of variceal bleeding is associated with further bleeding under atezolizumab–bevacizumab in patients with HCC. Liver International. 2022 Dec;42(12):2843–54.

31. Iwamoto H, Shimose S, Noda Y, et al. Initial Experience of Atezolizumab Plus Bevacizumab for Unresectable Hepatocellular Carcinoma in Real-World Clinical Practice. Cancers. 2021 Jun 3;13(11):2786.

32. Hiraoka A, Kumada T, Tada T, et al. Atezolizumab plus bevacizumab treatment for unresectable hepatocellular carcinoma: Early clinical experience. Cancer Reports. 2022 Feb;5(2):e1464.

33. Hiraoka A, Kumada T, Tada T, et al. Early experience of atezolizumab plus bevacizumab treatment for unresectable hepatocellular carcinoma BCLC-B stage patients classified as beyond up to seven criteria – Multicenter analysis. Hepatology Research. 2022 Mar;52(3):308–16.

34. Cheon J, Yoo C, Hong JY, et al. Efficacy and safety of atezolizumab plus bevacizumab in Korean patients with advanced hepatocellular carcinoma. Liver International. 2022 Mar;42(3):674–81.

35. Hatanaka T, Kakizaki S, Hiraoka A, et al. Prognostic impact of C-reactive protein and alpha-fetoprotein in immunotherapy score in hepatocellular carcinoma patients treated with atezolizumab plus bevacizumab: a multicenter retrospective study. Hepatol Int. 2022 Oct;16(5):1150–60.

36. De Castro T, Jochheim LS, Bathon M, et al. Atezolizumab and bevacizumab in patients with advanced hepatocellular carcinoma with impaired liver function and prior systemic therapy: a real-world experience. Ther Adv Med Oncol. 2022 Jan;14:175883592210802.

37. Himmelsbach V, Pinter M, Scheiner B, et al. Efficacy and Safety of Atezolizumab and Bevacizumab in the Real-World Treatment of Advanced Hepatocellular Carcinoma: Experience from Four Tertiary Centers. Cancers. 2022 Mar 28;14(7):1722.

38. Kim J, Nam HC, Kim CW, et al. Comparative Analysis of Atezolizumab Plus Bevacizumab and Hepatic Artery Infusion Chemotherapy in Unresectable Hepatocellular Carcinoma: A Multicenter, Propensity Score Study. Cancers. 2023 Aug 24;15(17):4233.

39. D'Alessio A, Fulgenzi CAM, Nishida N, et al. Preliminary evidence of safety and tolerability of atezolizumab plus bevacizumab in patients with hepatocellular carcinoma and Child-Pugh A and B cirrhosis: A real-world study. Hepatology. 2022 Oct;76(4):1000–12.

40. Tada T, Kumada T, Hiraoka A, et al. Safety and efficacy of atezolizumab plus bevacizumab in elderly patients with hepatocellular carcinoma: A multicenter analysis. Cancer Medicine. 2022 Oct;11(20):3796–808.

41. Tanaka T, Hiraoka A, Tada T, et al. Therapeutic efficacy of atezolizumab plus bevacizumab treatment for unresectable hepatocellular carcinoma in patients with Child-Pugh class A or B liver function in real-world clinical practice. Hepatology Research. 2022 Sep;52(9):773–83.

42. Chon YE, Cheon J, Kim H, et al. Predictive biomarkers of survival in patients with advanced hepatocellular carcinoma receiving atezolizumab plus bevacizumab treatment. Cancer Medicine. 2023 Feb;12(3):2731–8.

43. Shimose S, Iwamoto H, Tanaka M, et al. Association between Adverse Events and Prognosis in Patients with Hepatocellular Carcinoma Treated with Atezolizumab Plus Bevacizumab: A Multicenter Retrospective Study. Cancers. 2022 Sep 1;14(17):4284.

44. Vithayathil M, D'Alessio A, Fulgenzi CAM, et al. Impact of older age in patients receiving atezolizumab and bevacizumab for hepatocellular carcinoma. Liver International. 2022 Nov;42(11):2538–47.

45. Fulgenzi CAM, Cheon J, D'Alessio A, et al. Reproducible safety and efficacy of atezolizumab plus bevacizumab for HCC in clinical practice: Results of the AB-real study. European Journal of Cancer. 2022 Nov;175:204–13.

46. Rimini M, Rimassa L, Ueshima K, et al. Atezolizumab plus bevacizumab versus lenvatinib or sorafenib in non-viral unresectable hepatocellular carcinoma: an international propensity score matching analysis. ESMO Open. 2022 Dec;7(6):100591.

47. Hatanaka T, Kakizaki S, Hiraoka A, et al. Comparative efficacy and safety of atezolizumab and bevacizumab between hepatocellular carcinoma patients with viral and non-viral infection: A Japanese multicenter observational study. Cancer Medicine. 2023 Mar;12(5):5293–303.

48. Wu YL, Fulgenzi CAM, D'Alessio A, et al. Neutrophil-to-Lymphocyte and Platelet-to-Lymphocyte Ratios as Prognostic Biomarkers in Unresectable Hepatocellular Carcinoma Treated with Atezolizumab plus Bevacizumab. Cancers. 2022 Nov 26;14(23):5834.

49. Sinner F, Pinter M, Scheiner B, et al. Atezolizumab Plus Bevacizumab in Patients with Advanced and Progressing Hepatocellular Carcinoma: Retrospective Multicenter Experience. Cancers. 2022 Dec 2;14(23):5966.

50. Su CW, Teng W, Lin PT, et al. Similar efficacy and safety between lenvatinib versus atezolizumab plus bevacizumab as the first-line treatment for unresectable hepatocellular carcinoma. Cancer Med. 2023 Mar;12(6):7077–89.

51. Hiraoka A, Kumada T, Tada T, et al. Relationship of Atezolizumab plus Bevacizumab Treatment with Muscle Volume Loss in Unresectable Hepatocellular Carcinoma Patients: Multicenter Analysis. Liver Cancer. 2023;12(3):209–17.

52. Tada T, Kumada T, Hiraoka A, et al. New prognostic system based on inflammation and liver function predicts prognosis in patients with advanced unresectable hepatocellular carcinoma treated with atezolizumab plus bevacizumab: A validation study. Cancer Medicine. 2023 Mar;12(6):6980–93.

53. Tada T, Kumada T, Hiraoka A, et al. Adverse events as potential predictive factors of therapeutic activity in patients with unresectable hepatocellular carcinoma treated with atezolizumab plus bevacizumab. Cancer Medicine. 2023 Apr;12(7):7772–83.

54. Rimini M, Persano M, Tada T, et al. Survival outcomes from atezolizumab plus bevacizumab versus Lenvatinib in Child Pugh B unresectable hepatocellular carcinoma patients. J Cancer Res Clin Oncol. 2023 Aug;149(10):7565–77.

55. Vithayathil M, D'Alessio A, Fulgenzi CAM, et al. Impact of body mass index in patients receiving atezolizumab plus bevacizumab for hepatocellular carcinoma. Hepatol Int. 2023 Aug;17(4):904–14.

56. Tada F, Hiraoka A, Tada T, et al. Efficacy and safety of atezolizumab plus bevacizumab treatment for unresectable hepatocellular carcinoma patients with esophageal–gastric varices. J Gastroenterol. 2023 Nov;58(11):1134–43.

57. Ohama H, Hiraoka A, Tada T, et al. Comparison between Atezolizumab Plus Bevacizumab and Lenvatinib for Hepatocellular Carcinoma in Patients with Child-Pugh Class B in Real-World Clinical Settings. Oncology. 2023;101(9):542–52.

58. Tada T, Kumada T, Hiraoka A, et al. Comparison of prognostic impact of atezolizumab plus bevacizumab versus lenvatinib in patients with intermediate-stage hepatocellular carcinoma. Liver International. 2024 Jan;44(1):113–24.
