# Supplementary material for: Heterogeneity in adverse events related to atezolizumab-bevacizumab for hepatocellular carcinoma reported in real-world studies
Source: JHEP Rep. 2024 Aug 22;6(11):101190. doi: 10.1016/j.jhepr.2024.101190 (PMC11550199; doi:10.1016/j.jhepr.2024.101190)
Supplement: Multimedia component 4 [file mmc4.pdf]

Heterogeneity in adverse events related to atezolizumab-bevacizumab for hepatocellular carcinoma reported in real-world studies

Claudia Campani[1,2,†], **Dimitrios Pallas**[3,†], Sabrina Sidali[1], Olga Giouleme[4], Lorraine Blaise[1,3], Véronique Grando[1,3], Gisele Nkontchou[1,3], Alix Demory[1,3], Pierre Nahon[1,3], Nathalie Ganne-Carrié[1,3], Jean-Charles Nault[1,3,*]

JHEP Reports **2024**. vol. 6 | 1–13

Check for updates

Background & Aims: Safety data for patients with hepatocellular carcinoma (HCC) treated with atezolizumab-bevacizumab in the real-world setting remain uncertain. Thus, the aim of this study was to evaluate the incidence of adverse events (AEs) in patients with HCC treated with atezolizumab-bevacizumab in the literature.

Methods: In this systematic review and meta-analysis, we searched PubMed for original studies reporting percentages of AEs in patients with HCC receiving atezolizumab-bevacizumab between 2020 to 2023, using the search terms "Atezolizumab/Bevacizumab", "HCC" and "Adverse events". We summarized the incidence of AEs and performed a meta-analysis in order to evaluate the incidence of AEs reported in the literature.

Results: A total of 30 studies (3,867 patients) were included. The analysis revealed heterogeneity in AE reporting, with arterial hypertension, proteinuria, and fatigue being the most frequently reported AEs whereas incidence of bleeding was reported in 66.7% of the studies and rare immune-related AEs were reported in 26.7% of the studies. The meta-analysis revealed pooled incidence rates of 79% for any grade AEs: 56% for grade 1/2 and 30% for grade ≥3. While the pooled rates of hypertension, anorexia, bleeding, pruritus, rash, and thyroid dysfunction were similar to those reported in the IMbrave150 trial, higher rates were observed in the literature for proteinuria, fatigue, ALT and AST elevations and gastrointestinal perforation. For grade ≥3 AEs, the percentages were consistent with the IMbrave150 trial, except for lower incidences of arterial hypertension and thrombosis in the literature. The exposure-adjusted incidence rates for proteinuria (55.7%), hypertension (45.3%) and fatigue (33.6%) were high. Heterogeneity was observed in the analysis of AEs across articles within the same cohorts of patients.

Conclusion: We observed a significant variability in AE reporting for atezolizumab-bevacizumab treatment in HCC in the literature, underscoring the need for standardized reporting practices.

© 2024 The Author(s). Published by Elsevier B.V. on behalf of European Association for the Study of the Liver (EASL). This is an open access article under the CC BY license (http://creativecommons.org/licenses/by/4.0/).

Introduction

The combination of monoclonal antibodies targeting PD-L1 (programmed death-ligand 1) (atezolizumab) and VEGF (vascular endothelial growth factor) (bevacizumab) represents the new first-line standard of care for patients with unresectable hepatocellular carcinoma (HCC).[1,2] In the IMbrave150 trial, the atezolizumab-bevacizumab combination demonstrated improvements in overall survival (OS), progression-free survival (PFS) and objective response rate, as well as patient-reported outcomes compared to sorafenib.[3,4] Adverse events (AEs) of any grade occurred in 98% of patients treated with atezolizumab-bevacizumab *vs.* 99% in patients treated with sorafenib with 49% experiencing severe AEs *vs.* 33% in the sorafenib arm. The most common treatment-related AEs were proteinuria, arterial hypertension, increased aspartate aminotransferase (AST), and fatigue with a percentage of AEs leading to treatment withdrawal of 22%.[3,4] The most common immune-mediated AEs were rash (22%) and thyroid dysfunction (19%).[3,4] While a high rate of AST increase was also observed (53%), not all cases could be adjudicated to atezolizumab and immune-related hepatitis. Moreover, the percentage of corticosteroid use for immune-related AEs was not reported. In the atezolizumab-bevacizumab arm, there were six cases of bleeding, five gastrointestinal and one non-gastrointestinal hemorrhage, with a single case of intraperitoneal hemorrhage among patients treated with sorafenib. However, eligibility for IMbrave 150 trial was restricted to patients without prior exposure to systemic therapy, with preserved liver function (Child-Pugh A), and optimal control of portal hypertension.[3,4] For this reason, the safety data remain uncertain for patients treated with atezolizumab-bevacizumab in the real-world setting, including rare AEs such as gastrointestinal perforation, bleeding, thrombosis and immune-related AEs requiring

* Corresponding author. Address: AP-HP, Avicenne Hospital, Hepatology Unit, Cordeliers Research Center, 125 rue de Stalingrad 93000 Bobigny, France.
E-mail address: naultjc@gmail.com (J.-C. Nault).
† Co first authorship
https://doi.org/10.1016/j.jhepr.2024.101190

corticosteroids.[5–8] Accurate and consistent reporting of AEs is crucial for evaluating the safety profile of any therapeutic regimen, guiding clinical decision-making, and informing patient management. The analysis of incidence of AEs in studies from patients treated in the real-world setting is also useful to identify potential new toxicity signals not reported in the phase III clinical trials. The incidence of the different AEs associated with systemic treatments in other solid cancers across studies published in the literature is highly heterogeneous, both for lower grade and more severe AEs.[9–11] Inconsistencies in the reporting of AEs can stem from differences in study design (retrospective *vs.* prospective, monocentric, multicentric), sample size, patient demographics, and the way AEs are collected. These discrepancies complicate the interpretation of safety data and can obscure the true incidence and severity of AEs. However, this issue was not studied in patients with advanced HCC treated with systemic treatment.

We aimed to perform a systematic review and meta-analysis of the literature to assess the frequencies of AEs reported in studies evaluating atezolizumab-bevacizumab in patients with advanced HCC in order to evaluate the need for a more precise standardization of AE reports in the literature.

Material and methods

Search strategy

A systematic search for records from 14 May 2020 to 25 October 2023 in PubMed Central was performed using different combinations of the following keywords: "Atezolizumab plus Bevacizumab" AND "Hepatocellular Carcinoma", OR "Atezolizumab plus Bevacizumab" AND "HCC", OR "Atezolizumab and Bevacizumab" AND Hepatocellular Carcinoma", OR "Atezolizumab and Bevacizumab" AND "HCC", OR "Atezolizumab-Bevacizumab" AND "Hepatocellular Carcinoma" AND "Adverse events", OR "Atezolizumab-Bevacizumab" AND "HCC" AND "Adverse events", OR "Atezolizumab-Bevacizumab" AND "Hepatocellular Carcinoma", OR "Atezolizumab-Bevacizumab" AND "HCC". This study was conducted in accordance with PRISMA (Preferred Reporting Items for Systematic Review and Meta-Analysis) guidelines.[12]

Study selection and data extraction

The studies identified by the search were subsequently evaluated according to the following inclusion and exclusion criteria. Inclusion criteria: articles written in English, target population of the original paper was patients with HCC treated with atezolizumab-bevacizumab, general or specific percentages of AEs occurring during atezolizumab-bevacizumab treatment were reported. Exclusion criteria: phase Ib and phase III randomized clinical trial evaluating atezolizumab-bevacizumab, case-reports, systematic reviews and meta-analyses, sub-analyses of phase Ib and phase III trials, articles reporting AEs occurring when atezolizumab-bevacizumab was used in combination with other treatments (*e.g.* loco-regional treatments), articles reporting AEs occurring when atezolizumab-bevacizumab was used as adjuvant treatment, articles reporting AEs occurring during therapy with immune checkpoint inhibitors including atezolizumab-bevacizumab, but where the percentages of AEs for this specific combination, among all the systemic treatments, were not clearly reported.

Published systematic reviews and meta-analyses were screened to ensure that all studies reporting AEs during atezolizumab-bevacizumab were included.

Two reviewers (DP and CC) independently screened titles and abstracts of the articles extracted. The full texts of the eligible articles were then independently reviewed. Any discrepancies in article selection were resolved by discussion with a third expert reviewer (JCN). Data extraction from the included studies was then performed independently by two reviewers (DP and CC). The complete list of variables extracted from the included studies are reported in Table S1.

The outcomes of interest were the reporting of AEs and the incidences of these AEs during atezolizumab-bevacizumab treatment. In particular, we recorded general percentages of any AEs regardless of their severity, percentages of any AEs divided by severity grade according to the classification used in each study, and percentages of each specific AE regardless of grade and divided by severity grades. We recorded if frequent potential immune-related AEs, such as rash, hepatitis and thyroid disorder, were reported (and their incidence), as well as less common immune-related AEs (such as colitis, pneumonitis, nephritis, neuropathy, myositis, adrenal insufficiency, hypophysitis and/or rheumatological diseases) and the use of corticosteroids to treat immune-related AEs.

Statistical analysis

First, we conducted a descriptive analysis to assess the number of studies reporting each AE, as well as evaluating the characteristics of the included population. For each AE reported in these studies, we evaluated the distribution of percentages by calculating the minimum, maximum, median, and interquartile range (raw data). Distinct analyses encompassing all grades of AEs and each severity grade (grade 1/2, grade 3/4, grade 5) were performed. Initially, we performed the analysis by excluding studies from the same research group to eliminate potential overlap. If multiple publications reported on the same study population, the one with the higher number of patients was included. We considered studies to be from the same research group when they were explicitly declared as such or when we identified recurring authors across different papers.

In a second step, we conducted a meta-analysis using the meta-packages and metaprop functions of R statistical software version 4.1.1. The proportion of patients experiencing each AE and the corresponding number of patients were extracted from each study along with the total number of patients included in each study. To account for between-study heterogeneity, an inverse variance random-effects model was employed, assuming a common between-study variance. The DerSimonian-Laird estimator was used to estimate the between-study variance (τ^2), which quantifies the amount of heterogeneity among the included studies. Forest plots were generated to visualize the individual study estimates along with the overall pooled estimate, with confidence intervals representing the uncertainty around the pooled estimate.

Next, we studied the occurrence of AEs adjusted to the length of exposure to atezolizumab-bevacizumab. For studies where the median treatment duration with atezolizumab-bevacizumab was available, the outcome of interest was reported as exposure-adjusted incidence rate. Incidence rates per 100 patient-years were calculated by dividing the total

number of patients experiencing each AE by the sum of all patients' time (in 100 years) of exposure during the treatment period.[13] Finally, we analyzed differences in reported AEs across the same cohorts, including all identified reports for each cohort. For each study, we also recorded the median overall survival (OS) and progression-free survival (PFS) when available, along with the HR and its corresponding 95% CIs, as well as the number of patients included in the survival analysis. Values of p <0.05 were considered significant. All the analyses were conducted in R statistical software version 4.1.1 (R: A Language and Environment for Statistical Computing, Vienna, Austria).

Results

Study selection and characteristics

Our systematic literature search initially identified 622 relevant publications. After the removal of case reports, systematic reviews and meta-analyses, 94 records remained. With the evaluation of the full-text articles, we further excluded 32 irrelevant publications including articles without clear data about AEs (n = 14) and combination therapies (n = 1). Consequently 62 eligible studies involving patients were included (Fig. 1; Table 1). Thirty studies were further excluded as they were from the same research group, suggesting potential overlap among patients, two studies were excluded as they were phase Ib and phase III randomized-controlled trials. Finally, 30 studies considering 3,867 patients were included in the qualitative analysis (Fig. 1). Most of the included studies (83%) were retrospective, whereas four studies (17%) collected data prospectively (Fig. 2A). Sixty-seven percent of the studies were multicentric, with the majority conducted in Asia (90%) (Table S2). The median age of patients was 72 years and ranged from 53.7 to 76 years. Most of the patients reflected the real-world setting of atezolizumab-bevacizumab prescription rather than the inclusion criteria of the IMbrave 150 trial, given that most studies (83%) included patients with Child-Pugh class ≥A and that 75% of the studies included patients who received atezolizumab-bevacizumab as first, second, or later lines of treatment. Temporary or definitive withdrawal of

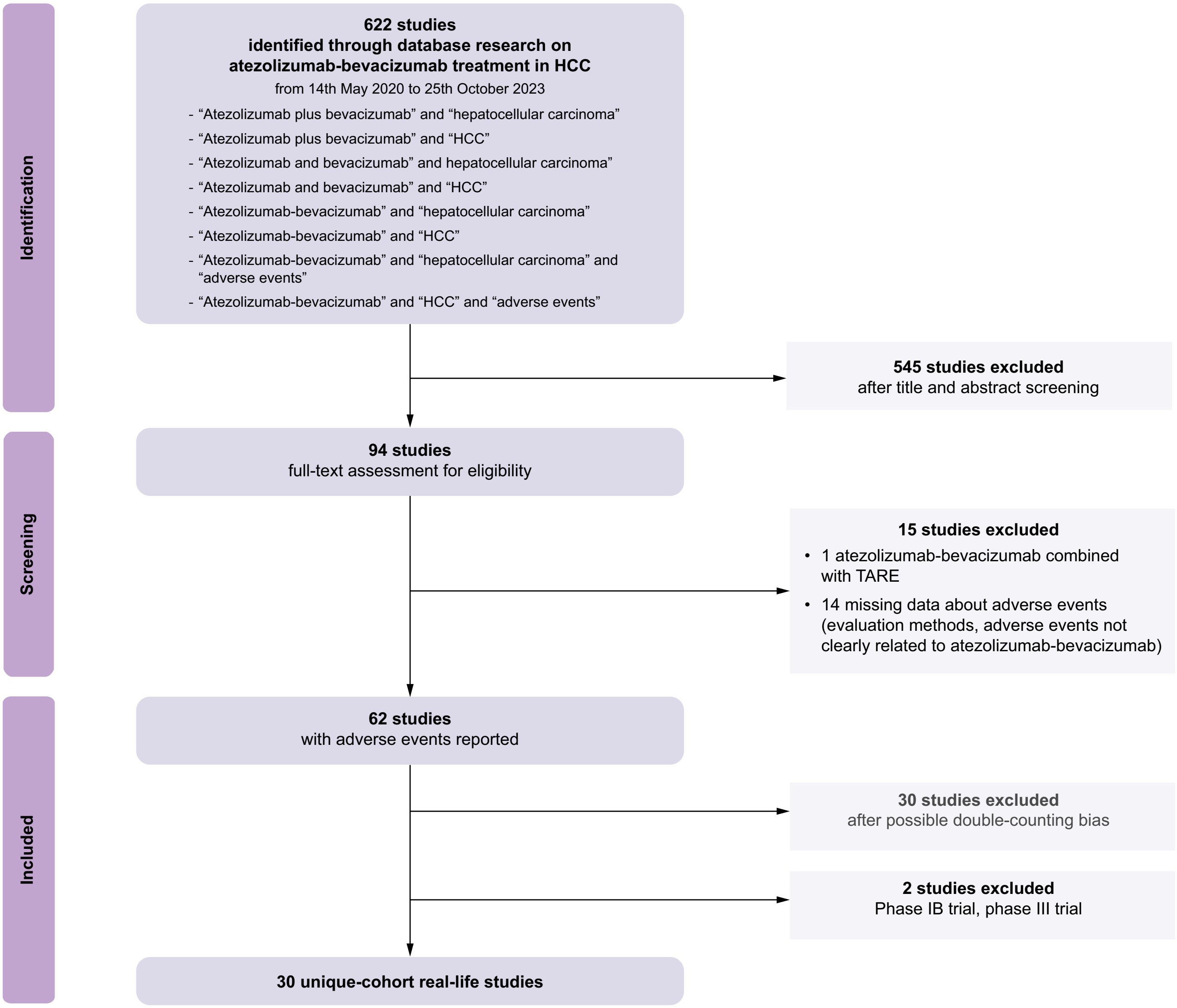

Fig. 1. Flow chart of the study (adapted from PRISMA 2020 flow diagram). HCC, hepatocellular carcinoma; TARE, trans-arterial radioembolization.

Table 1. Description of the studies included in the analysis (n = 30).

References	Retrospective/ prospective	Monocentric/ multicentric	Country	Number of patients	Primary endpoint	Median OS	Median PFS	Child-Pugh B/C (%)	HBV (%)	HCV (%)	MASLD (%)	Alcohol (%)	Male (%)	Median age (years)	Adverse events evaluation
Kuzuya *et al.*[24]	Retrospective	Monocentric	Asia	23	ORR, safety	NA	NA	0	17,4	21,7	NA	NA	78,3	74	CTCAE v5.0
Sho *et al.*[25]	Retrospective	Multicentric	Asia	58	ORR, DCR	NA	NA	7,8	25	23,4	NA	NA	85,9	72	CTCAE v4.0
Ando *et al.*[26]	Retrospective	Monocentric	Asia	40	ORR, safety	NA	NA	0	NA	NA	NA	NA	75	69	CTCAE v5.0
Hayakawa *et al.*[27]	Retrospective	Monocentric	Asia	52	ORR, safety, DCR	Not reached	4,7	7,7	19,2	38,5	NA	25	80,8	73	CTCAE v5.0
Eso *et al.*[28]	Prospective	Monocentric	Asia	40	Biomarker, ORR	NA	5,0	5	15	32,5	NA	NA	87,5	70,5	CTCAE v5.0
Chuma *et al.*[29]	Retrospective	Multicentric	Asia	94	ORR, safety	NA	NA	13,8	19,1	33	NA	NA	77,6	73	CTCAE v4.0
Yang-Cheng *et al.*[30]	Retrospective	Multicentric	Asia	35	OS, PFS, ORR, DCR	22,2	5,2	17	63	17	NA	NA	89	61	CTCAE v5.0
Wang *et al.*[31]	Retrospective	Monocentric	Asia	48	Biomarker, PFS	NA	NA	12,5	58,3	27,1	NA	NA	79,2	62	CTCAE v5.0
Maesaka *et al.*[32]	Prospective	Multicentric	Asia	66	ORR	Not reached	8,8	2,9	NA	NA	NA	NA	76,8	76	CTCAE v4.0
Teng *et al.*[33]	Retrospective	Monocentric	Asia	89	Biomarker OS	NA	NA	14,6	77,5	11,2	NA	NA	84,3	61,3	CTCAE v5.0
Tomonari *et al.*[34]	Retrospective	Multicentric	Asia	71	Biomarker, OS, PFS	NA	NA	8,4	11,3	42,2	NA	NA	81,7	71	CTCAE v5.0
Ochi *et al.*[35]	Retrospective	Multicentric	Asia	242	Biomarker, PFS, ORR, DCR	Not reached	NA	NA	NA	NA	NA	NA	79,3	NA	CTCAE v5.0
Sugimoto *et al.*[36]	Prospective	Multicentric	Asia	31	OS, PFS, ORR, safety	NA	NA	13	13	35	29	23	84	72	CTCAE v4.0
Niizeki *et al.*[37]	Retrospective	Multicentric	Asia	152	OS, PFS, ORR	Not reached	8,3	NA	13,6	39,1	NA	NA	76,4	73	CTCAE v5.0
Nakagawa *et al.*[38]	Retrospective	Multicentric	Asia	123	PFS, safety	NA	NA	7,3	19,5	30,1	25,2	NA	82,9	NA	CTCAE v5.0
Casadei-Gardini *et al.*[39]	Retrospective	Multicentric	Both	864	OS, TTP, safety	16,4	NA	7,2	23,5	31,3	6,8	NA	79,9	72	CTCAE v5.0
Charonpongsuntorn *et al.*[40]	Prospective	Multicentric	Asia	30	OS, PFS, safety, QOL	10,2	6,7	0	63,3	10	10	16,7	90	58	CTCAE v4.0
Unome *et al.*[41]	Retrospective	Multicentric	Asia	69	OS, PFS, ORR	12,5	5,4	11,6	17,4	31,9	23,2	17,4	79,7	74,4	CTCAE v5.0
Cheon *et al.*[42]	Retrospective	Multicentric	Asia	169	OS, PFS, ORR	NA	NA	17,7	66,8	6,5	NA	14,7	82,2	61	CTCAE v5.0
Zeng *et al.*[43]	Retrospective	Monocentric	Asia	30	OS, PFS, ORR, DCR	16,6	7,3	NA	93,3	3,3	NA	NA	86,7	53,7	CTCAE v4.0
Matoya *et al.*[44]	Retrospective	Multicentric	Asia	110	Biomarker OS, PFS	Not reached	NA	8,2	12,7	29,1	NA	8,2	80,9	74	NA
Kulkarni *et al.*[45]	Retrospective	Multicentric	Asia	67	OS	12,0	8,0	64,1	19,4	16,4	55,2	7,5	86,5	61	NA
Tokunaga *et al.*[46]	Retrospective	Multicentric	Asia	100	OS, TTP	21,9	NA	16	20	30	NA	35	84	NA	CTCAE v5.0
Jost-Brinkmann *et al.*[47]	Retrospective	Monocentric	Europe	100	OS, PFS, ORR, DCR, safety, TTP	NA	6,3	39	NA	NA	NA	NA	87	67	CTCAE v5.0
Takaki *et al.*[48]	Retrospective	Multicentric	Asia	268	OS, PFS, safety	15,4	8,0	25,4	10,4	31,3	NA	25,1	78,7	75	CTCAE v5.0
Fukushima *et al.*[49]	Retrospective	Multicentric	Asia	150	OS, PFS	NA	NA	12	18	32,6	NA	NA	80	72	CTCAE v5.0
Yano *et al.*[50]	Retrospective	Multicentric	Asia	136	OS, ORR, safety	10,2	4,0	4	NA	NA	NA	32	77	73,1	CTCAE v5.0
Tada *et al.*[51]	Retrospective	Multicentric	Asia	506	ORR	NA	NA	10,6	16,8	33	NA	21,9	77,8	74	CTCAE v5.0
Takada *et al.*[52]	Retrospective	Monocentric	Asia	61	Biomarker, safety	19,0	7,7	26	13	46	NA	NA	83,6	74,4	CTCAE v5.0
Larrey *et al.*[18]	Prospective	Monocentric	Europe	43	Safety	12,0	7,9	14	25.6	48.8	41.9	44.2	79.1	65	NA

CTCAE, Common Terminology Criteria for Adverse Events; DCR, disease control rate; ORR, objective response rate; OS, overall survival; PFS, progression-free survival; QOL, quality of life; TTP, time to progression. The number of patients reported were those included in the safety analysis.

atezolizumab-bevacizumab was reported in 11 (36.6%) studies with percentages ranging from 0 to 70%, whereas permanent withdrawal was described in only nine studies (30.0%) with percentages ranging from 0 to 41%. The median OS of patients treated with atezolizumab-bevacizumab was reported in 16 (53.3%) of the 30 studies. Specifically, five of these studies indicated that the median OS had not yet been reached, while the median survival in the remaining 11 studies was 15.4 months (IQR 12-19 months). The hazard ratios (HRs) and 95% CIs were provided in only four studies. The median PFS was reported in 15 (50%) of the studies, with a median value of 7 months (IQR 5.3-8 months). HRs and their corresponding 95% CIs were reported in just two studies.

Availability of data on AEs

First, we assessed how, and which, AEs were reported across the 30 studies. Adverse events were reported using Common Terminology Criteria for Adverse Events (CTCAE) version 4 in 20.0% of studies and CTCAE version 5 in 70.0% of the studies (Table S2), while in three studies (10%) the system used to grade AEs was not reported. In less than half of the studies (40.0%), the percentage of patients developing AEs of any grade regardless of causality was reported. Moreover, only 33% of the studies reported the total percentage of grade 1/2 AEs, and 40% the total percentage of grade ≥3 AEs. The most frequently reported AE was arterial hypertension, which was reported in 86.7% of the 30 included studies (Fig. 2A). The other most frequently reported AEs were proteinuria (83.3%), fatigue (83.3%), anorexia (73.3%), diarrhea (70.0%), rash (70.0%), bleeding (66.7%), thyroid dysfunction (60.0%), and fever (53.3%), while other AEs were reported in less than 50% of the studies (Fig. 2A). The percentages of thromboembolic events were available in only four studies (13.3%), while perforation was evaluated in only two studies (6.7%).

Incidence of frequent AEs: raw data and meta-analysis

First, we evaluated the raw data on the incidence of AEs based on the severity grade as well as their variations assessed by IQR (Fig. 2B describing the raw data). For all AEs, the dispersion of the percentages, as indicated by the higher IQR, was significantly higher for grade 1/2 compared to grade 3/4 AEs (Fig. 2B describing the raw data). Specifically, the IQR of grade 1/2 AEs was nine-fold greater for rash, six-fold greater for both diarrhea and ALT (alanine aminotransferase) elevation, and five-fold greater for fatigue, compared to grade 3/4 AEs. The availability of data on each AE, and median OS and PFS, is depicted in Fig. S1 and no significant correlation was found between the percentage of each AE and median OS or PFS (Table S3).

Next, we performed a meta-analysis in order to accurately describe the incidence of AEs. The incidence rates of any grade, grade 1/2, and grade 3 or higher treatment-related AEs (trAEs) were 79% (95% CI 68%-89%), 56% (95% CI 38%-73%) and 30% (95% CI 20%-41%), respectively. In the IMbrave 150 trial, the incidence rates of any grade, grade 1/2, grade 3 or higher trAEs were 98.2%, 41.7% and 56.5%, respectively. Thus, we conducted a separate meta-analysis to evaluate the incidence of 14 different AEs linked to atezolizumab-bevacizumab, synthesizing data from the 30 independent studies and comparing these percentages with those described in the phase Ib and phase III studies (Figs 3 and 4). When considering all-grade trAEs, significant heterogeneity was observed among the included studies, requiring the use of random-effects models to obtain reliable pooled estimates. In contrast, for grade ≥3 AEs, the rates among studies were more consistent with heterogeneity not significant in most cases. This allows for the use of fixed-effects models to provide more precise estimates. While the pooled rates of arterial hypertension (28%), anorexia (19%), bleeding (8.9%), fever (15%), pruritus (16%), rash (9%) and thyroid dysfunction (7%) of any grade were similar to those reported in the IMbrave 150 trial; the rates of proteinuria (31%), fatigue (27%), ALT elevation (20%), AST elevation (35%) and perforation (2%) were higher, while the rates of diarrhea (8%) and thrombosis (9%) were lower (Figs 3 and 4). When focusing on grade ≥3 AEs, the percentages were consistent with those reported in the IMbrave 150 trial, except for arterial hypertension (5% in our analysis *vs.* 15.2% in IMbrave 150) and thrombosis (0% in our analysis *vs.* 2.7% in IMbrave150), which were more frequently observed in the IMbrave 150 trial.

Exposure-adjusted incidence rates of frequent AEs

Seven of the 30 studies including a total of 512 patients reported the median duration of treatment with atezolizumab-bevacizumab, which ranged from 2.8 to 9.4 months. For these studies, we calculated the exposure-adjusted incidence rate (EAIR) for each of the 14 AEs we considered (Figs 5 and S1). Our analysis revealed that the AEs of any grade with the highest EAIR were proteinuria (55.7%), arterial hypertension (45.3%), and fatigue (33.6%). Similarly, when considering grade ≥3 AEs, the highest EAIRs were observed for proteinuria (5.7%), arterial hypertension (4.5%) and bleeding (4.5%). The delta between the unadjusted incidence rate *vs.* the EAIR of AEs of any grade was 29.08% for proteinuria, 23.6% for arterial hypertension, 17.5% for fatigue, 11.5% for anorexia, 10.2% for AST elevation and 10.2% for bleeding.

Incidence of bleeding, thrombosis, perforation and rare immune-related AEs

Furthermore, among the 21 studies that reported the occurrence of immune-related AEs (including mainly the more frequent immune-related AEs such as thyroid dysfunction, diarrhea, hepatitis and rash), no information was provided regarding the use of corticosteroids in 80.7% of the cases. Eight percent (IQR 5.6-9.0%) of patients treated with atezolizumab-bevacizumab received corticosteroids for immune-related AEs among the four studies reporting these data (Table S4). The main reason for corticosteroid therapy was predominantly related to immune-mediated hepatitis. However, the terminology and definition of this AE varied between the studies (liver dysfunction, hepatitis, liver injury, etc.) (Table S4). Only 8 (26.7%) of the studies reported the incidence of "rare" immune-mediated AEs such as colitis, pneumonitis, nephritis, neuropathy, myositis, adrenal insufficiency, hypophysitis and/or rheumatological diseases.

Regarding other severe AEs, 20 (66.7%) studies reported data about bleeding, 4 (13.8%) studies the occurrence of thromboembolic events, and only 2 (6.9%) studies the occurrence of gastrointestinal perforation (Fig. 2A).

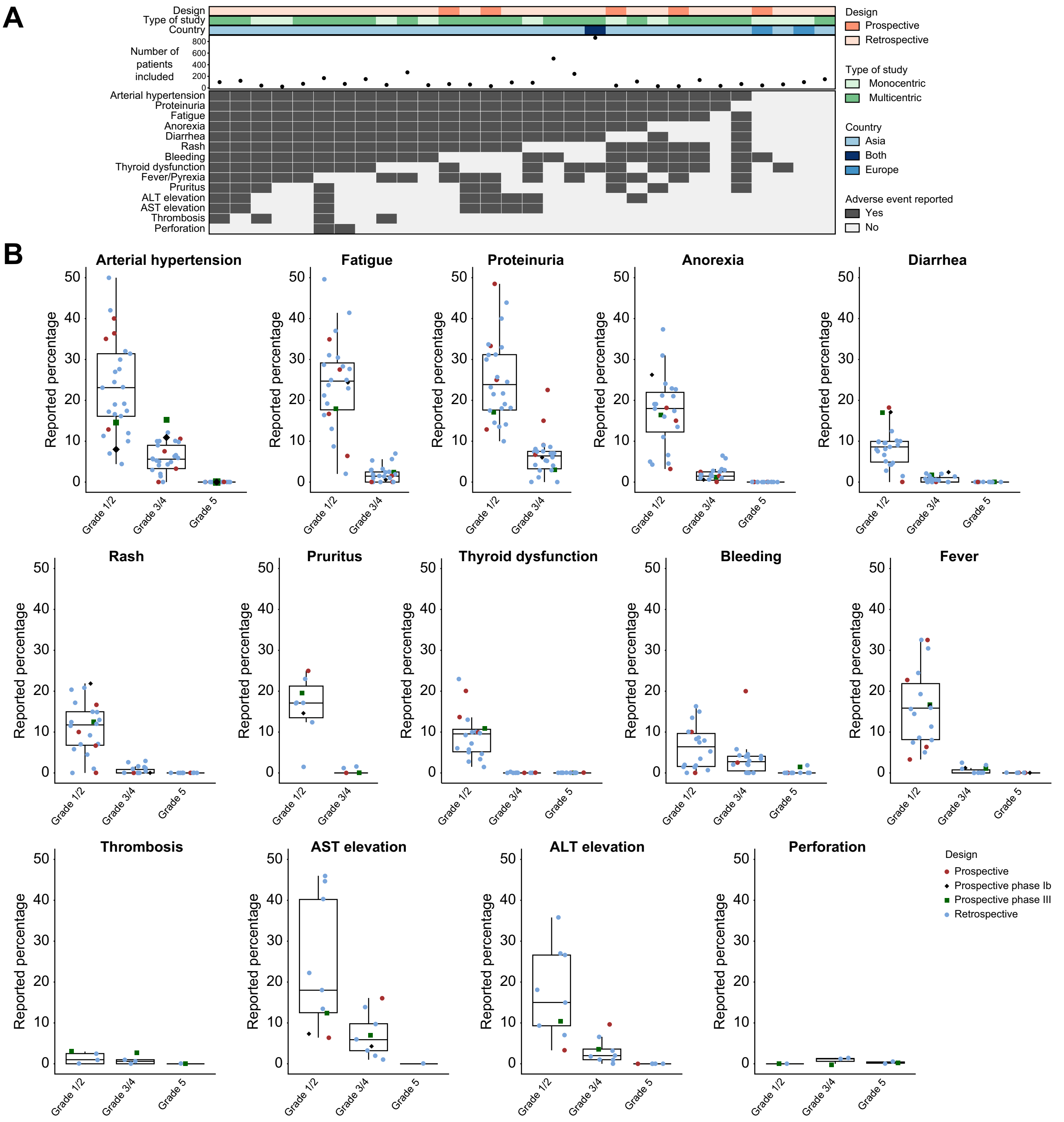

Fig. 2. Raw data on adverse events reported across 30 studies. (A) Heatmap illustrating the presence or absence of descriptions of adverse events, regardless of their grade, across 30 included studies. Each column represents a study analyzed with its annotations. (B) Boxplots displaying the distribution of percentages of each adverse event (raw data) across studies, categorized according to their severity. Boxes represent the IQR of the data, the horizontal line the median value and whiskers the range of the data, extending to 1.5 times the IQR from the quartiles. Individual data points corresponding to each study are plotted and colored differently based on the type of study. On the graph of each adverse event, the percentages reported in the phase Ib and phase III studies have also been included for comparison; however, the data from phase Ib and phase III were not used in the calculation of the median and IQR. ALT, alanine aminotransferase; AST, aspartate aminotransferase.

Bleeding of any grade, regardless of type, occurred at a median frequency of 9.1% (IQR 6.0-15.5%) similar to that reported in the phase III trial (7.0%). However, bleeding was described in a highly heterogeneous manner across the studies (Table S5). Among the 20 studies reporting the percentages of bleeding, six did not report the cause of bleeding, three reported only bleeding such as epistaxis or hemoptysis (but not the incidence of gastrointestinal bleeding) and finally 11

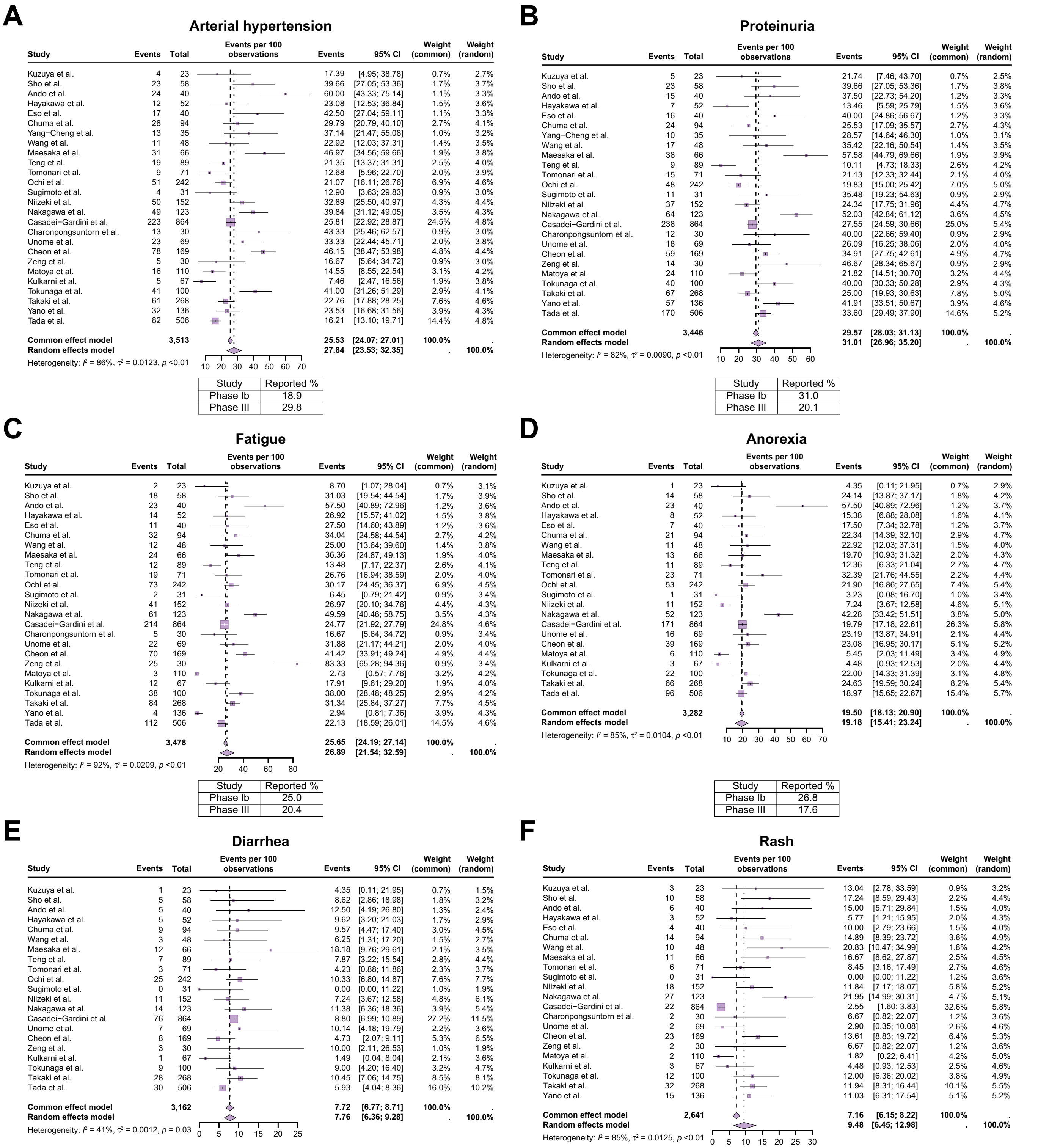

Study	Reported %
Phase Ib	18.9
Phase III	29.8

Study	Reported %
Phase Ib	31.0
Phase III	20.1

Study	Reported %
Phase Ib	25.0
Phase III	20.4

Study	Reported %
Phase Ib	26.8
Phase III	17.6

Study	Reported %
Phase Ib	19.5
Phase III	18.8

Study	Reported %
Phase Ib	21.9
Phase III	12.5

Fig. 3. Meta-analysis for frequent adverse events occurring during atezolizumab-bevacizumab treatment. Each forest plot represents a specific adverse event category, regardless of the severity grade. (A) Arterial hypertension. (B) Proteinuria. (C) Fatigue. (D) Anorexia. (E) Diarrhea. (F) Rash. Squares indicate estimates; size of squares, study weights; whiskers, 95% CIs; diamonds, mean estimates.

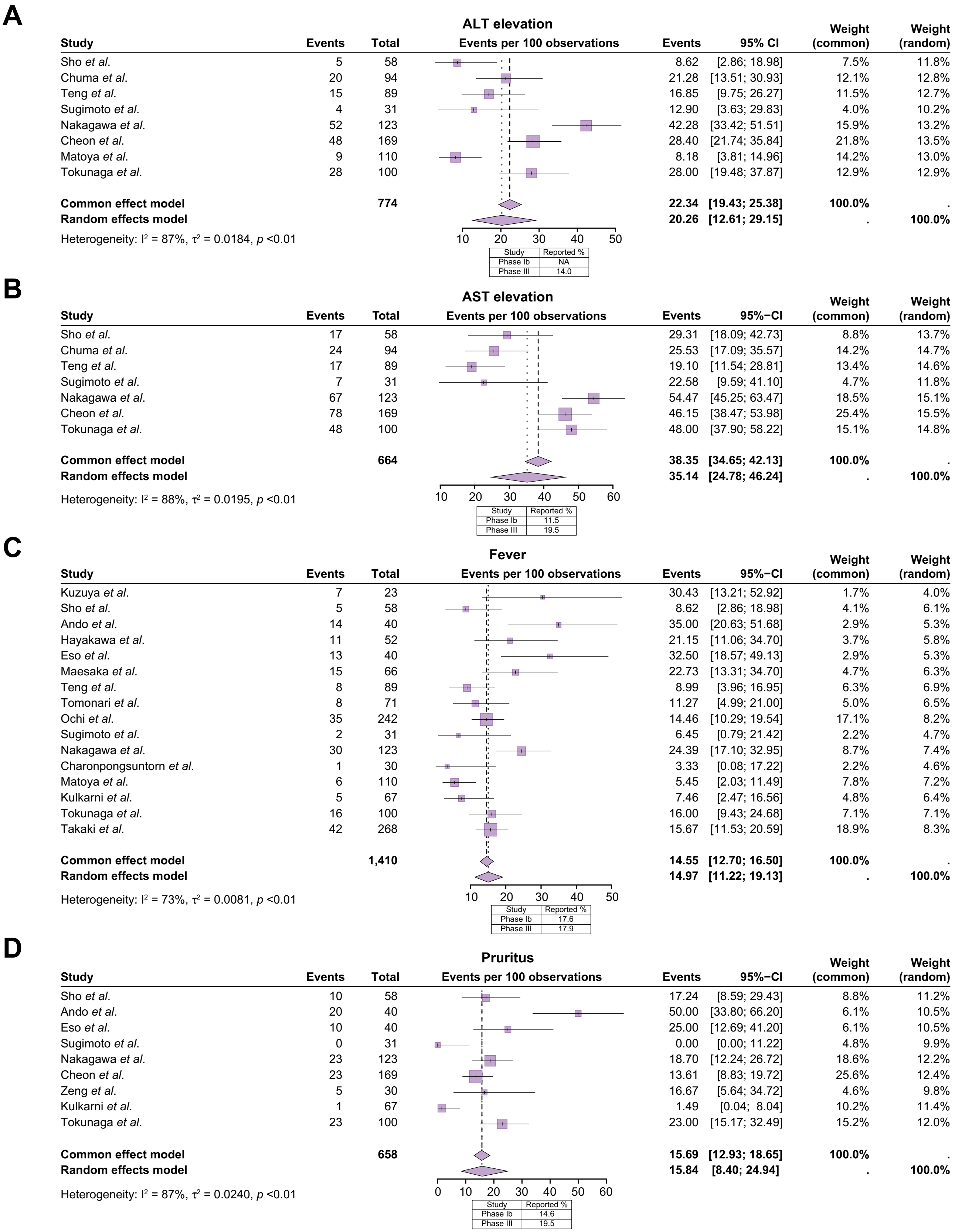

Fig. 4. Meta-analysis for the less frequent adverse events occurring during atezolizumab-bevacizumab treatment. Each forest plot represents a specific adverse event category, regardless of the severity grade. (A) ALT elevation. (B) AST elevation. (C) Fever. (D) Pruritus. Squares indicate estimates; size of squares, study weights; whiskers, 95% CIs; diamonds, mean estimates. ALT, alanine aminotransferase; AST, aspartate aminotransferase.

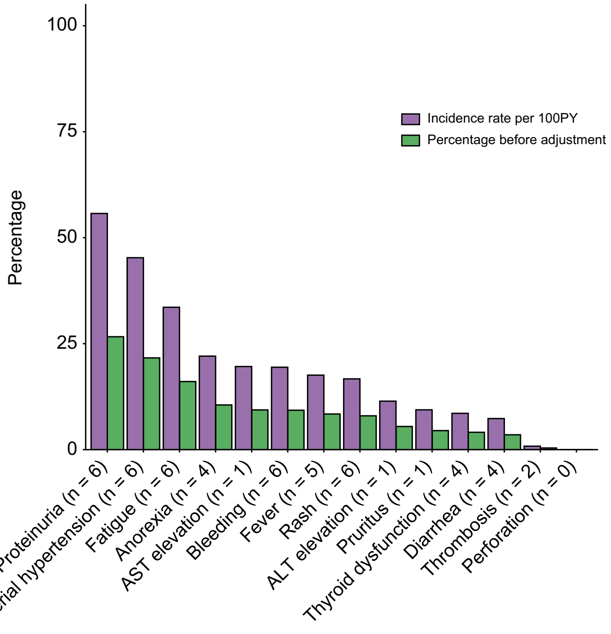

Fig. 5. Exposure-adjusted incidence rate of adverse events in patients with HCC treated with atezolizumab-bevacizumab. The blue set of columns represents exposure-adjusted rates per 100 PY, while the yellow set represents percentages reported across the seven studies for which the median duration of treatment with atezolizumab-bevacizumab was known. ALT, alanine aminotransferase; AST, aspartate aminotransferase; PY, patient-years.

reported the rate of gastrointestinal bleeding (including precise data on variceal bleeding in only four of the studies). Among the four studies reporting the rate of variceal bleeding, the incidence of variceal bleeding was 4.5% (IQR 1.7%-16.9%). Conversely, venous thrombosis was reported with a median frequency of 1.8% (IQR 1.1%-4.0%) which was lower compared to the incidence observed in the IMbrave trial (5.7%). In contrast, the incidence of gastrointestinal perforation was slightly higher in published studies (median 2.0%, IQR 1.8%-2.5%) compared to the incidence reported in the IMbrave trial (0.3%).

Intra and inter-cohort variability in AE reporting

We evaluated the rate of 14 distinct AEs across multiple publications (n = 62) from six different cohorts, which included overlapping patient populations: the RELPEC group (14 publications, 29 to 506 patients according to the study), a collaborative group including European and non-European centers (10 publications, 65 to 864 patients according to the study), a Japanese collaborative group not included in RELPEC (3 publications, 51 to 152 patients according to the study), a German group not included in the previous European group (2 publications, 50 to 100 patients according to the study), a Taiwanese and a Korean collaborative group (2 and 5 publications respectively, 46 to 89 patients and 86 to 169 patients, respectively) (Figs 6, S2 and S3; Table S6). Among the 14 RELPEC publications, only arterial hypertension, anorexia and fatigue were reported in all the studies of the group and proteinuria in 13 out of the 14 studies (Fig. 6). While the percentages of all grades of arterial hypertension were quite similar among the same studies from the RELPEC group, ranging from 11.1% to 20%, the percentages of all-grade anorexia, fatigue and proteinuria were extremely different, ranging from 12.3% to 44.8%, 11.1% to 37.7%, and 10.5% to 35.8%, respectively (Figs 6 and S2). The second largest cohort was the one composed of European and non-European patients with 10 different articles (Figs 6 and S2). Reported AEs were heterogeneous, underlined by the fact that none of the AEs were reported across all 10 studies. The most frequently reported AEs in this group were arterial hypertension (8 out of 10 studies), fatigue (7 out of 10 studies), proteinuria (6 out of 10 studies), and rash (6 out of 10 studies). In this cohort, the percentage of arterial hypertension, fatigue, proteinuria, and rash, regardless of grade, ranged between 0-5% and 30% (Figs 6 and S2). Finally, The Korean cohort was composed of five studies and the percentage of AEs was very similar across all these studies (Figs 6 and S2).

Discussion

AEs associated with systemic treatments are a critical concern in clinical trials and in clinical practice. To guide AE classification and grading in clinical trials, the US National Cancer Institute introduced the CTCAE and the CONSORT International Committee issued different guidelines to standardize AE data reporting.[14,15] Despite these measures, evidence shows inadequate AE reporting in clinical trials and real-life studies, with poor adherence to these guidelines.[16,17] Moreover, prospective *vs.* retrospective study, the use of patient-reported outcomes *vs.* clinician-reported outcomes, and the duration of follow-up influence the reported incidence and severity of AEs.

Our systematic review and meta-analysis of data from 30 studies including 3,867 patients with HCC treated with atezolizumab-bevacizumab sought to provide a comprehensive assessment of trAEs available in the literature. First, few studies have reported the percentage of temporary or permanent atezolizumab-bevacizumab discontinuation with available data in only 11 and 9 studies, respectively. Moreover, the percentage of temporary or permanent treatment suspensions ranged from 0% to 70% and permanent suspensions from 0% to 41%, suggesting a very heterogenous assessment. Our analysis reveals a significant heterogeneity across studies in terms of types and percentages of AEs reported, and criteria used for their classification. Most studies reported the frequent AEs occurring in the IMbrave 150 trial, such as arterial hypertension (89.7%), proteinuria (86.2%), and fatigue (86.2%). However, data on AST elevation, which was found in 16% of patients in IMbrave 150, was available in only 23.3% of the studies in the literature. When we calculated the pooled rates for the 14 different AEs regardless of the grade of severity based on a meta-analysis, similar results to IMbrave 150 were found for arterial hypertension, anorexia, bleeding, fever, pruritus, rash and thyroid dysfunction of any grade.[3,4] The results of our meta-analysis indicate that, overall, no additional safety signals were identified with the use of atezolizumab-bevacizumab in clinical practice. Nevertheless, it was difficult to draw definitive conclusions for venous thrombosis, bleeding, perforation, and immune-related AEs because these complications were rarely reported in the literature. For all the 14 AEs analyzed in our systematic review, we observed that the incidence of grade 1/2 AEs was more variable across studies compared to more severe AEs. This data highlights the

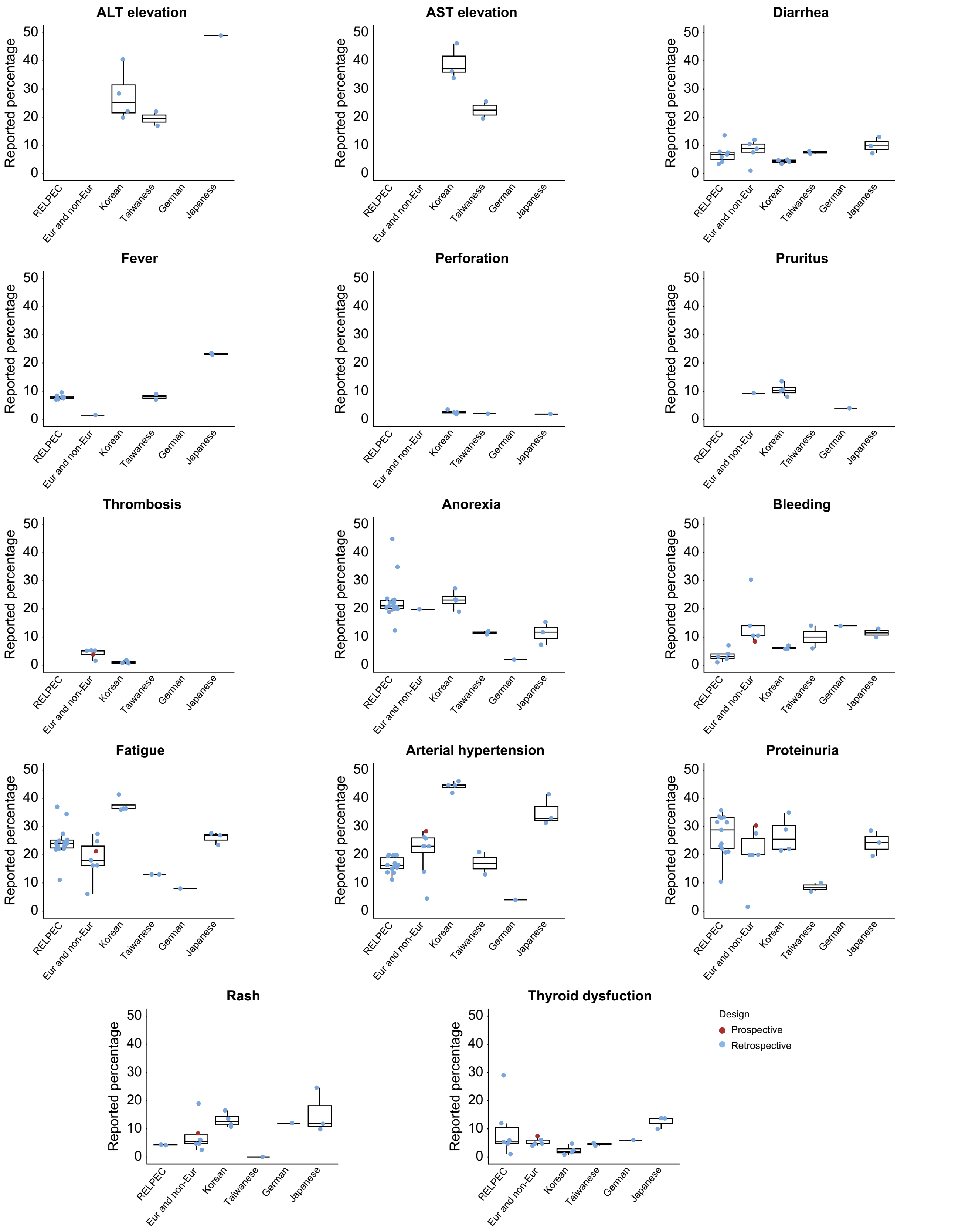

Fig. 6. Percentage of adverse events reported in studies published by the same research group. Boxplots displayed the distribution of percentages of each any grade adverse event (raw data) reported in different studies published by the same group: RELPEC group (14 studies), European and non-European cohorts (10 studies), Korean cohorts (5 studies), Taiwanese cohorts (2 studies), German cohorts (2 studies) and Japanese cohorts not included in RELPEC group (3 studies). Boxes represent the IQR of the data, the horizontal line the median value and whiskers the range of the data, extending to 1.5 times the IQR from the quartiles. Individual data points corresponding to each study. ALT, alanine aminotransferase; AST, aspartate aminotransferase.

necessity for a more rigorous assessment of reported AEs, particularly because the grading of these AEs may be influenced by the need to initiate or modify baseline therapy, as seen in cases of thyroid dysfunction or arterial hypertension. These results underscore that physicians tend to pay greater attention and provide more accurate reporting in case of severe AEs.

The rare AEs, like bleeding, thromboembolic events and gastrointestinal perforation, were also poorly described.[3,4] In the IMbrave150 trial, all patients were required to have an upper endoscopy screening for esophageal and gastric varices within the last 6 months and required adequate treatment of varices before inclusion.[3,4] Despite the stringent inclusion criteria, the bleeding rate reported in the IMbrave 150 trial was 7%, warranting further investigation in real-world clinical practice. However, in most of the studies we reviewed, the occurrence of bleeding, particularly gastrointestinal bleeding, was not adequately reported. Indeed, 30% of studies which reported the percentage of bleeding did not specify the causes of bleeding, and the occurrence of acute variceal bleeding was reported in only four studies. More granular data is required to assess the risk of bleeding due to portal hypertension in these patients in clinical practice, as well as to identify specific risk factors for bleeding in this population.[18]

Moreover, immune checkpoint inhibitors can cause immune-related adverse events.[19] Although toxicity can affect any organ, the most commonly reported irAEs involve the skin, the endocrine system, and the digestive tract including the liver.[13] In the IMbrave 150 trial, the most frequent immune-related AE reported was immune hepatitis, defined as all incidents of diagnostic and laboratory abnormalities.[3,4] The reporting of immune-related AEs was extremely heterogeneous across publications. Furthermore, the development of immune-related AEs is sometimes difficult to differentiate from complications of cirrhosis.[20] Additionally, it is noteworthy that in these studies, the number of patients with cirrhosis is often not specified and the assessment of liver function not well-described, potentially resulting in interpretative bias, particularly concerning liver-related AEs and bleeding. Very few studies reported information about the necessity of corticosteroid therapy with only five studies providing details on corticosteroid use. Providing this information is essential, as these AEs require precise management, and detailed reports could enhance clinicians' knowledge and capabilities in effectively treating these AEs.

The EAIR is a crucial metric in oncology as it provides an accurate evaluation of the incidence of AEs by considering the lengths of exposure to treatment.[13] However, among the 30 studies we evaluated, only seven reported data on median treatment duration. The EAIRs for proteinuria, arterial hypertension, and fatigue were notably high, suggesting that the onset of some AEs may manifest over time, depending on the period of exposure to the treatment, underlying the need for close monitoring and management of these AEs in clinical practice. Moreover, the correct evaluation of AE occurrence is crucial, as their presence may be variably associated with treatment response, as has already been demonstrated with sorafenib.[21,22] Finally, we also observed variability in the reporting of 14 distinct AEs across publications from six different cohorts. The RELPEC group showed wide ranges for all-grade anorexia, fatigue, and proteinuria, while the European and non-European collaborative group also exhibited substantial variability in arterial hypertension, fatigue, proteinuria, and rash percentages.

Some limitations of this study need to be acknowledged. First, the different studies included patients with heterogeneous characteristics in terms of demographics, etiology, liver function, and treatments prior to atezolizumab-bevacizumab. Most of the endpoints of these studies were not focused on the description of AEs, probably explaining the variation in terms of reporting AEs. Furthermore, most of the studies were conducted in Japan, which may hinder the applicability of the data to Western countries.[5,23] More studies from other regions are needed to accurately understand the real AE profile for atezolizumab-bevacizumab. In addition, we considered the AEs that emerged during the treatment with atezolizumab-bevacizumab and not throughout the entire follow-up period of the patient, which may impact the identification of late-onset AEs that appear after the treatment has been discontinued.

In conclusion, our systematic review and meta-analysis provides insights into the heterogeneity of safety profiles in patients with HCC treated with atezolizumab-bevacizumab in the real-world setting. The significant heterogeneity in AE reporting and the underreporting of severe AEs such as bleeding, thrombosis and perforation, as well as corticosteroid use for immune-related AEs, highlight areas for improvement in clinical research.

Affiliations

[1]*Cordeliers Research Center, Sorbonne University, Inserm, Paris Cité University, "Functional Genomics of Solid Tumors" Team, Ligue Nationale Contre le Cancer Accredited Team, Labex OncoImmunology, F-75006 Paris, France;* [2]*Internal Medicine and Hepatology Unit, Department of Experimental and Clinical Medicine, University of Firenze, Florence, Italy;* [3]*Liver Unit, Avicenne Hospital, APHP, Bobigny, France, University Sorbonne Paris Nord, Bobigny, France;* [4]*Department of Gastroenterology and Hepatology, 2nd Propedeutic Department of Internal Medicine, Medical School, Aristotle University, 54124 Thessaloniki, Greece*

Abbreviations

AE, adverse event; AST, aspartate aminotransferase; CTCAE, Common Terminology Criteria for Adverse Events; EAIR, exposure-adjusted incidence rate; HCC, hepatocellular carcinoma; OS, overall survival; PFS, progression-free survival; PRO, patient-reported outcomes; trAE, treatment-related adverse event.

Financial support

The authors did not receive any financial support to produce this manuscript.

Conflict of interest

Jean-Charles Nault received research funding from Bayer and Ipsen. Pierre Nahon has received honoraria from and/or consults for AstraZeneca, Bayer, Bristol-Myers Squibb, Eisai, Gilead, Guerbet, Ipsen, and Roche. He received research grants from AstraZeneca, AbbVie, Bristol-Myers Squibb and Eisai. Sabrina Sidali has received honoraria from AstraZeneca. NGC received travel and congress fees, Consulting fees or honoraria for lectures, presentations, speaker's bureaus from Abbvie, Gilead, Intercept and Roche.

Please refer to the accompanying ICMJE disclosure forms for further details.

Authors' contributions

Contributions to conception and design: CC, DP, JCN, Acquisition of data: CC, DP, JCN, Analysis and interpretation of data: CC, DP, JCN, Drafting, revising, and the manuscript content: CC, DP, SS, OG, LB, VG, GN, AD, PN, NG, JCN, Final approval of the version to be published: CC, DP, SS, OG, LB, VG, GN, AD, PN, NG, JCN.

Data availability statement

The datasets generated and analyzed during the current systematic review and meta-analysis are available from the corresponding author upon reasonable request.

Supplementary data

Supplementary data to this article can be found online at https://doi.org/10.1016/j.jhepr.2024.101190.

References

Author names in bold designate shared co-first authorship

[1] Reig M, Forner A, Rimola J, et al. BCLC strategy for prognosis prediction and treatment recommendation: the 2022 update. J Hepatol 2022 Mar;76(3):681–693.

[2] Llovet JM, Kelley RK, Villanueva A, et al. Hepatocellular carcinoma. Nat Rev Dis Primers 2021 Dec;7(1):6.

[3] Finn RS, Qin S, Ikeda M, et al. Atezolizumab plus bevacizumab in unresectable hepatocellular carcinoma. N Engl J Med 2020 May 14;382(20): 1894–1905.

[4] Cheng AL, Qin S, Ikeda M, et al. Updated efficacy and safety data from IMbrave150: atezolizumab plus bevacizumab vs. sorafenib for unresectable hepatocellular carcinoma. J Hepatol 2022 Apr;76(4):862–873.

[5] Pasta A, Calabrese F, Jaffe A, et al. Safety and efficacy of atezolizumab/bevacizumab in patients with hepatocellular carcinoma and impaired liver function: a systematic review and meta-analysis. Liver Cancer 2023 Oct 14:1–11.

[6] Xie E, Yeo YH, Scheiner B, et al. Immune checkpoint inhibitors for child-pugh class B advanced hepatocellular carcinoma: a systematic review and meta-analysis. JAMA Oncol 2023 Oct 1;9(10):1423.

[7] Joerg V, Scheiner B, D'Alessio A, et al. Efficacy and safety of atezolizumab/bevacizumab in patients with HCC after prior systemic therapy: a global, observational study. Hepatol Commun [Internet] 2023;7(11). Nov [cited 2024 May 20] Available from: https://journals.lww.com/10.1097/HC9.0000000000000302.

[8] Sultanik P, Campani C, Larrey E, et al. Portal hypertension is associated with poorer outcome and clinical liver decompensation in patients with HCC treated with Atezolizumab-Bevacizumab. Dig Liver Dis 2024 Mar:S1590865824003049.

[9] Yao Y, Liu Z, Zhang H, et al. Serious adverse events reporting in phase III randomized clinical trials of colorectal cancer treatments: a systematic analysis. Front Pharmacol 2021 Nov 18;12:754858.

[10] Or M, Liu B, Lam J, et al. A systematic review and meta-analysis of treatment-related toxicities of curative and palliative radiation therapy in non-small cell lung cancer. Sci Rep 2021 Mar 15;11(1):5939.

[11] Hsiehchen D, Watters MK, Lu R, et al. Variation in the assessment of immune-related adverse event occurrence, grade, and timing in patients receiving immune checkpoint inhibitors. JAMA Netw Open 2019 Sep 18;2(9):e1911519.

[12] Page MJ, McKenzie JE, Bossuyt PM, et al. The PRISMA 2020 statement: an updated guideline for reporting systematic reviews. BMJ 2021 Mar 29;n71.

[13] **Celsa C**, **Cabibbo G**, Fulgenzi CAM, et al. Characteristics and outcomes of immunotherapy-related liver injury in patients with hepatocellular carcinoma *vs.* other advanced solid tumours. J Hepatol 2023 Nov:S0168827823052728.

[14] https://ctep.cancer.gov/protocolDevelopment/electronic_applications/ctc.htm.

[15] Ioannidis JPA. Better reporting of harms in randomized trials: an extension of the CONSORT statement. Ann Intern Med 2004 Nov 16;141(10):781.

[16] Pitrou. Reporting of safety results in published reports of randomized controlled trials. Arch Intern Med 2009 Oct 26;169(19):1756.

[17] Sivendran S, Latif A, McBride RB, et al. Adverse event reporting in cancer clinical trial publications. JCO 2014 Jan 10;32(2):83–89.

[18] Larrey E, Campion B, Evain M, et al. A history of variceal bleeding is associated with further bleeding under atezolizumab–bevacizumab in patients with HCC. Liver Int 2022 Dec;42(12):2843–2854.

[19] Brahmer JR, Lacchetti C, Schneider BJ, et al. Management of immune-related adverse events in patients treated with immune checkpoint inhibitor therapy: American society of clinical oncology clinical practice guideline. JCO 2018 Jun 10;36(17):1714–1768.

[20] De Martin E, Michot JM, Rosmorduc O, et al. Liver toxicity as a limiting factor to the increasing use of immune checkpoint inhibitors. JHEP Rep 2020 Dec;2(6):100170.

[21] **Reig M**, **Torres F**, Rodriguez-Lope C, et al. Early dermatologic adverse events predict better outcome in HCC patients treated with sorafenib. J Hepatol 2014 Aug;61(2):318–324.

[22] **Rimola J**, **Díaz-González Á**, Darnell A, et al. Complete response under sorafenib in patients with hepatocellular carcinoma: relationship with dermatologic adverse events. Hepatology 2018 Feb;67(2):612–622.

[23] El Hajra I, Sanduzzi-Zamparelli M, Sapena V, et al. Outcome of patients with HCC and liver dysfunction under immunotherapy: a systematic review and meta-analysis. Hepatology 2023 Apr;77(4):1139–1149.

[24] Kuzuya T, Kawabe N, Hashimoto S, et al. Initial experience of atezolizumab plus bevacizumab for advanced hepatocellular carcinoma in clinical practice. CDP 2021 May 3;1(2):83–88.

[25] Sho T, Suda G, Ogawa K, et al. Early response and safety of atezolizumab plus bevacizumab for unresectable hepatocellular carcinoma in patients who do not meet IMbrave150 eligibility criteria. Hepatol Res 2021 Sep;51(9):979–989.

[26] Ando Y, Kawaoka T, Kosaka M, et al. Early tumor response and safety of atezolizumab plus bevacizumab for patients with unresectable hepatocellular carcinoma in real-world practice. Cancers 2021 Aug 5;13(16):3958.

[27] **Hayakawa Y**, **Tsuchiya K**, Kurosaki M, et al. Early experience of atezolizumab plus bevacizumab therapy in Japanese patients with unresectable hepatocellular carcinoma in real-world practice. Invest New Drugs 2022 Apr;40(2):392–402.

[28] Eso Y, Takeda H, Taura K, et al. Pretreatment neutrophil-to-lymphocyte ratio as a predictive marker of response to atezolizumab plus bevacizumab for hepatocellular carcinoma. Curr Oncol 2021 Oct 14;28(5):4157–4166.

[29] Chuma M, Uojima H, Hattori N, et al. Safety and efficacy of atezolizumab plus bevacizumab in patients with unresectable hepatocellular carcinoma in early clinical practice: a multicenter analysis. Hepatol Res 2022 Mar;52(3):269–280.

[30] **Lee YC**, **Huang WT**, Lee MY, et al. Bevacizumab and atezolizumab for unresectable hepatocellular carcinoma: real-world data in taiwan-tainan medical oncology group H01 trial. In Vivo 2023;37(1):454–460.

[31] Wang JH, Chen YY, Kee KM, et al. The prognostic value of neutrophil-to-lymphocyte ratio and platelet-to-lymphocyte ratio in patients with hepatocellular carcinoma receiving atezolizumab plus bevacizumab. Cancers 2022 Jan 11;14(2):343.

[32] Maesaka K, Sakamori R, Yamada R, et al. Comparison of atezolizumab plus bevacizumab and lenvatinib in terms of efficacy and safety as primary systemic chemotherapy for hepatocellular carcinoma. Hepatol Res 2022 Jul;52(7):630–640.

[33] **Teng W**, **Lin CC**, Su CW, et al. Combination of CRAFITY score with Alpha-fetoprotein response predicts a favorable outcome of atezolizumab plus bevacizumab for unresectable hepatocellular carcinoma. Am J Cancer Res 2022;12(4):1899–1911.

[34] Tomonari T, Tani J, Sato Y, et al. Initial therapeutic results of atezolizumab plus bevacizumab for unresectable advanced hepatocellular carcinoma and the importance of hepatic functional reserve. Cancer Med 2023 Feb;12(3):2646–2657.

[35] Ochi H, Kurosaki M, Joko K, et al. Usefulness of neutrophil-to-lymphocyte ratio in predicting progression and survival outcomes after atezolizumab–bevacizumab treatment for hepatocellular carcinoma. Hepatol Res 2023 Jan;53(1):61–71.

[36] Sugimoto R, Satoh T, Ueda A, et al. Atezolizumab plus bevacizumab treatment for unresectable hepatocellular carcinoma progressing after molecular targeted therapy: a multicenter prospective observational study. Medicine 2022 Oct 7;101(40):e30871.

[37] Niizeki T, Tokunaga T, Takami Y, et al. Comparison of efficacy and safety of atezolizumab plus bevacizumab and lenvatinib as first-line therapy for unresectable hepatocellular carcinoma: a propensity score matching analysis. Targ Oncol 2022 Nov;17(6):643–653.

[38] Nakagawa M, Inoue M, Ogasawara S, et al. Clinical effects and emerging issues of atezolizumab plus bevacizumab in patients with advanced hepatocellular carcinoma from Japanese real-world practice. Cancer 2023 Feb 15;129(4):590–599.

[39] **Casadei-Gardini A**, **Rimini M**, Tada T, et al. Atezolizumab plus bevacizumab *vs.* lenvatinib for unresectable hepatocellular carcinoma: a large real-life worldwide population. Eur J Cancer 2023 Feb;180:9–20.

[40] Charonpongsuntorn C, Tanasanvimon S, Korphaisarn K, et al. Efficacy, safety, and patient-reported outcomes of atezolizumab plus bevacizumab for unresectable hepatocellular carcinoma in Thailand: a multicenter prospective study. JCO Glob Oncol 2022 Dec;(8):e2200205.

[41] Unome S, Imai K, Takai K, et al. Changes in ALBI score and PIVKA-II within three months after commencing atezolizumab plus bevacizumab treatment affect overall survival in patients with unresectable hepatocellular carcinoma. Cancers 2022 Dec 10;14(24):6089.
[42] **Cheon J**, **Kim H**, Kim HS, et al. Atezolizumab plus bevacizumab in patients with child–Pugh B advanced hepatocellular carcinoma. Ther Adv Med Oncol 2023 Jan;15:175883592211485.
[43] Zeng H, Xu Q, Wang J, et al. The effect of anti-PD-1/PD-L1 antibodies combined with VEGF receptor tyrosine kinase inhibitors *vs.* bevacizumab in unresectable hepatocellular carcinoma. Front Immunol 2023 Jan 23;14: 1073133.
[44] Matoya S, Suzuki T, Matsuura K, et al. The neutrophil-to-lymphocyte ratio at the start of the second course during atezolizumab plus bevacizumab therapy predicts therapeutic efficacy in patients with advanced hepatocellular carcinoma: a multicenter analysis. Hepatol Res 2023 Jun;53(6):511–521.
[45] Kulkarni AV, Krishna V, Kumar K, et al. Safety and efficacy of atezolizumab-bevacizumab in real world: the first Indian experience. J Clin Exp Hepatol 2023 Jul;13(4):618–623.
[46] Tokunaga T, Tateyama M, Kondo Y, et al. Therapeutic modifications without discontinuation of atezolizumab plus bevacizumab therapy are associated with favorable overall survival and time to progression in patients with unresectable hepatocellular carcinoma. Cancers 2023 Mar 2; 15(5):1568.
[47] Jost-Brinkmann F, Demir M, Wree A, et al. Atezolizumab plus bevacizumab in unresectable hepatocellular carcinoma: results from a German real-world cohort. Aliment Pharmacol Ther 2023 Jun;57(11):1313–1325.
[48] Takaki S, Kurosaki M, Mori N, et al. Effects on survival of the adverse event of atezolizumab plus bevacizumab for hepatocellular carcinoma: a multicenter study by the Japan Red Cross Liver Study Group. Invest New Drugs 2023 Apr;41(2):340–349.
[49] Fukushima T, Morimoto M, Kobayashi S, et al. Association between immune-related adverse events and survival in patients with hepatocellular carcinoma treated with atezolizumab plus bevacizumab. The Oncologist 2023 Jul 5;28(7):e526–e533.
[50] Yano Y, Yamamoto A, Mimura T, et al. Factors associated with the response to atezolizumab/bevacizumab combination therapy for hepatocellular carcinoma. JGH Open 2023 Jul;7(7):476–481.
[51] Tada F, Hiraoka A, Tada T, et al. Efficacy and safety of atezolizumab plus bevacizumab treatment for unresectable hepatocellular carcinoma patients with esophageal–gastric varices. J Gastroenterol 2023 Nov;58(11) :1134–1143.
[52] Takada H, Yamashita K, Osawa L, et al. Significance of the autoantibody assay in predicting the development of immune-related adverse events in patients receiving atezolizumab plus bevacizumab combination therapy for unresectable hepatocellular carcinoma. Hepatol Res 2024 Feb;54(2):162–173.

Keywords: liver cancer; immunotherapy; adverse effects.
Received 6 July 2024; received in revised form 6 August 2024; accepted 8 August 2024; Available online 22 August 2024

Journal of Hepatology, Volume *6*

Supplemental information

Heterogeneity in adverse events related to atezolizumab-bevacizumab for hepatocellular carcinoma reported in real-world studies

Claudia Campani, Dimitrios Pallas, Sabrina Sidali, Olga Giouleme, Lorraine Blaise, Véronique Grando, Gisele Nkontchou, Alix Demory, Pierre Nahon, Nathalie Ganne-Carrié, and Jean-Charles Nault

Heterogeneity in adverse events related to atezolizumab-bevacizumab for hepatocellular carcinoma reported in real-world studies

Claudia Campani, Dimitrios Pallas, Sabrina Sidali, Olga Giouleme, Lorraine Blaise, Véronique Grando, Gisele Nkontchou, Alix Demory, Pierre Nahon, Nathalie Ganne-Carrié, Jean-Charles Nault

Table of contents

Supplementary figures 2

Supplementary tables 5

Supplementary figure legends 14

Supplementary references 15

Supplementary figures

Fig. S1

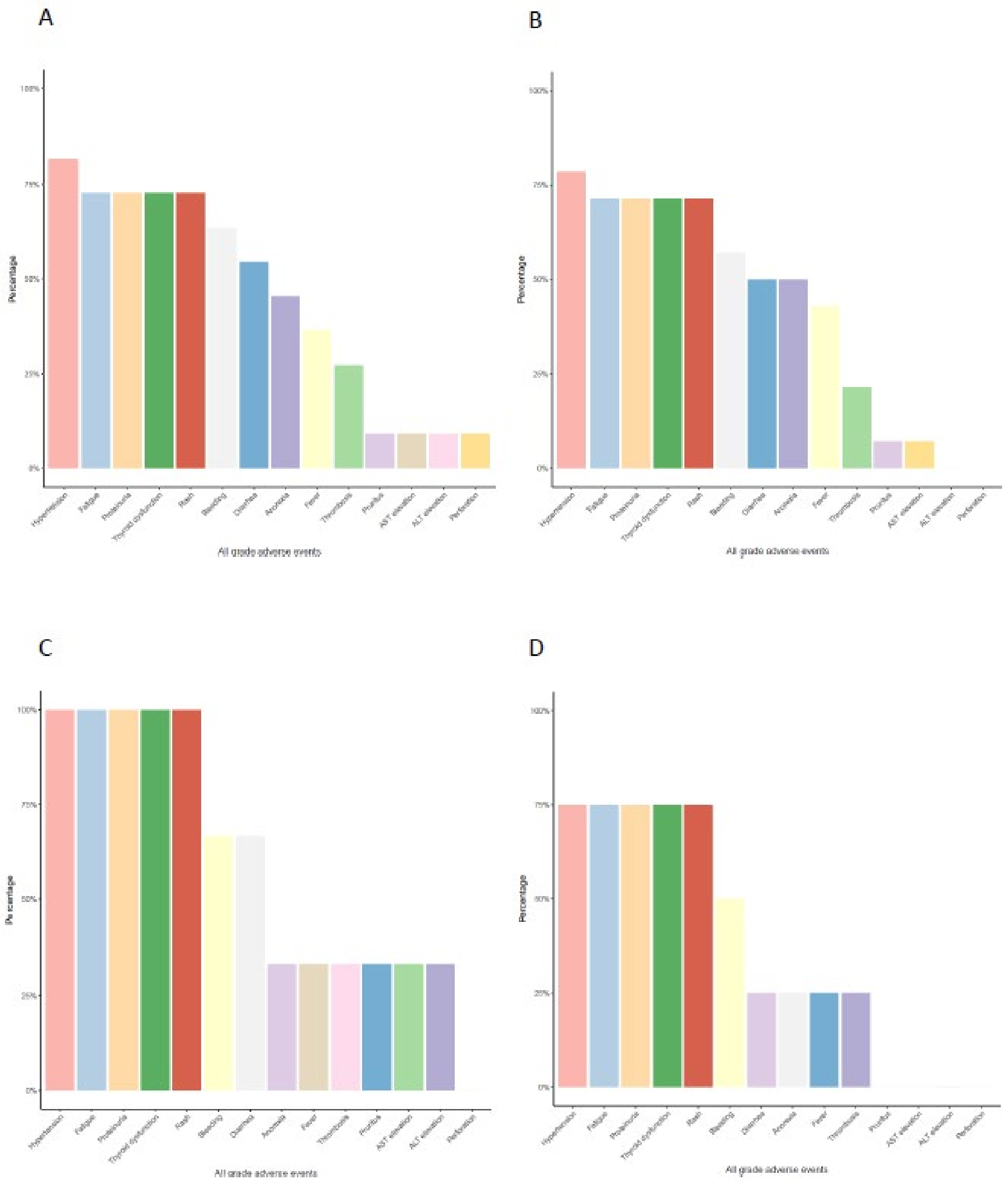

Fig. S2

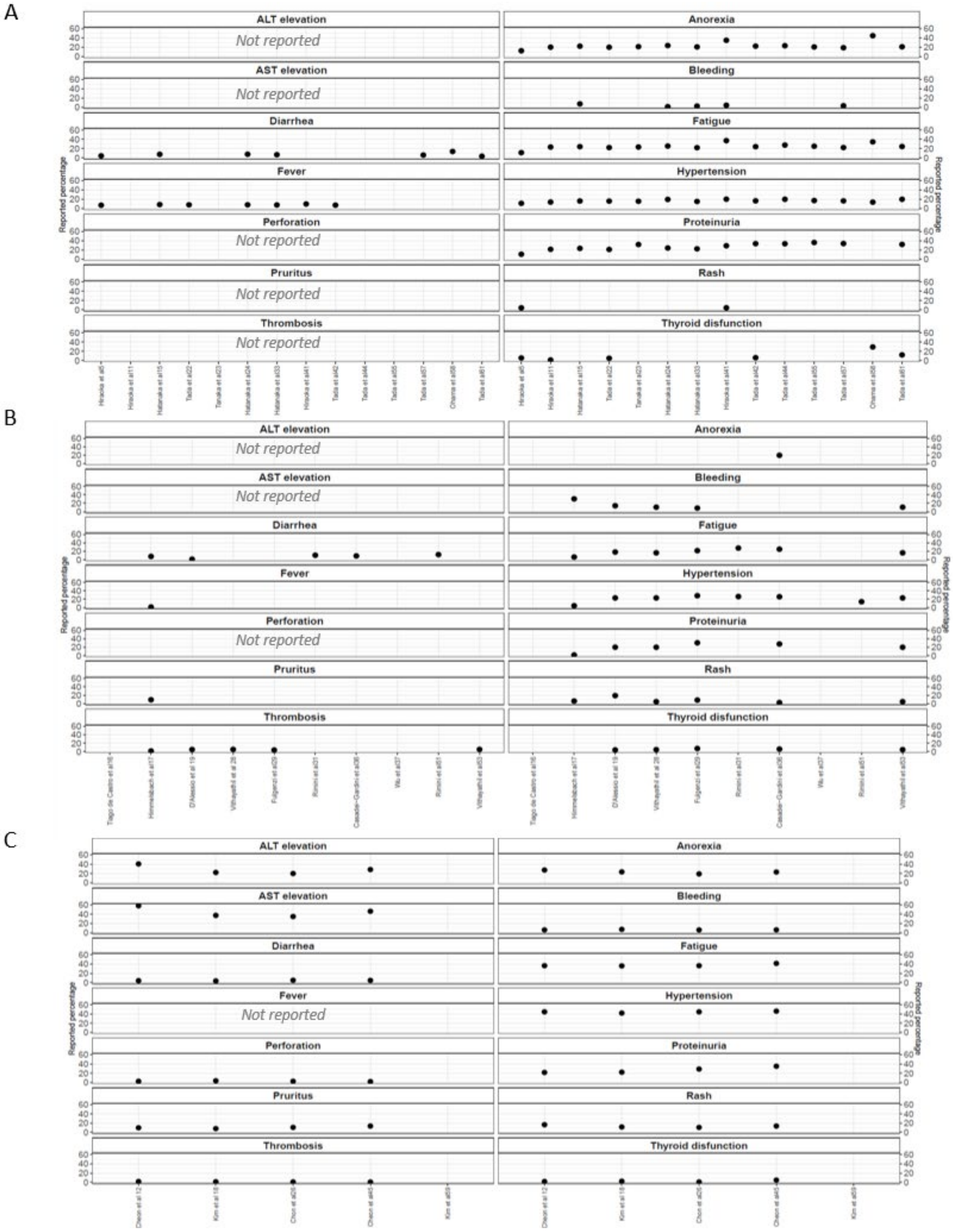
A
B
C
ALT elevation
AST elevation
Diarrhea
Fever
Perforation
Pruritus
Thrombosis
Anorexia
Bleeding
Fatigue
Hypertension
Proteinuria
Rash
Thyroid disfunction
Not reported
Reported percentage

Fig. S3

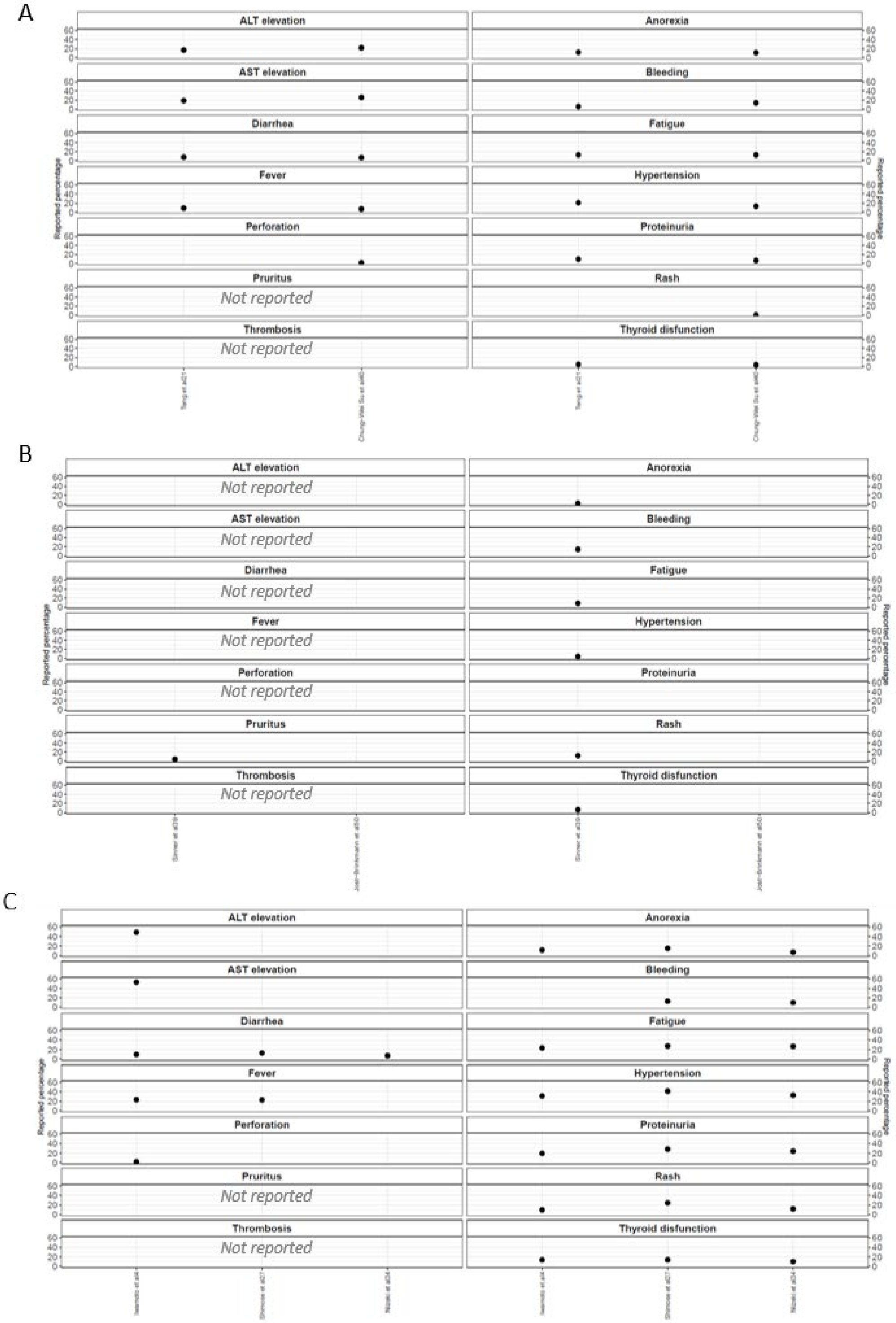
A
ALT elevation
Anorexia
AST elevation
Bleeding
Diarrhea
Fatigue
Fever
Hypertension
Perforation
Proteinuria
Pruritus
Not reported
Rash
Thrombosis
Not reported
Thyroid disfunction
Reported percentage
B
ALT elevation
Not reported
Anorexia
AST elevation
Not reported
Bleeding
Diarrhea
Not reported
Fatigue
Fever
Not reported
Hypertension
Perforation
Not reported
Proteinuria
Pruritus
Rash
Thrombosis
Not reported
Thyroid disfunction
Reported percentage
C
ALT elevation
Anorexia
AST elevation
Bleeding
Diarrhea
Fatigue
Fever
Hypertension
Perforation
Proteinuria
Pruritus
Not reported
Rash
Thrombosis
Not reported
Thyroid disfunction
Reported percentage

Supplementary tables

Table S1. Variables collected for each study

Authors
Journal
Impact factor
Title
Publication Date
Retrospective/Prospective
Monocentric/Multicentric
Asia/Europe
Cohorts
Number of patients included in safety analysis
Primary endpoint of the study
Median time of treatment with Atezo-Beva (months)
Median overall survival (months)
Median progression-free survival (months)
Number of patients included in survival analysis
Child-Pugh class A only (Yes/no)
Percentage of Child-Pugh class non-A
Atezo-Beva used as first line only
Percentage of HBV
Percentage of HCV
Percentage of metabolic syndrome
Percentage of alcohol
Percentage of male
Median Age patients Atezo-Beva
Reported adverse events (Yes/No)
Evaluation method of adverse events
Any grade all AE reported (Yes/No)
Any grade all AE (Percentage)
Grade1/2 all AE reported
Adverse events grade 1/2 all AE(Percentage)
Grade 3 all AE reported
Adverse events grade 3 all AE(Percentage)
Grade 4 all AE reported
Adverse events grade 4 all AE(Percentage)
Grade 5 all AE reported
Adverse events grade 5 all AE(death, Percentage)

Adverse events grade 5 (death causes)
Grade ≥3 all AE reported
Adverse events grade ≥3 all AE(Percentage)
Hypertension reported (Yes/No)
Hypertension (Percentage of all grade)
Hypertension (Percentage of grade 1/2)
Hypertension (Percentage of grade 3/4)
Hypertension (Percentage of grade 5)
Bleeding reported (Yes/No)
Bleeding (Percentage of all grade)
Type of Bleeding considered
Bleeding (Percentage of grade 1/2)
Bleeding (Percentage of grade 3/4)
Bleeding (Percentage of grade 5)
Thrombosis reported (Yes/No)
Thrombosis / PE (Percentage of all grade)
Thrombosis / PE (Percentage of grade 1/2)
Thrombosis /PE (Percentage of grade 3/4)
Thrombosis / PE (Percentage of grade 5)
Proteinuria reported (Yes/No)
Proteinuria Percentage of all grade
Proteinuria Percentage of (grade 1/2)
Proteinuria Percentage of (grade 3)
Fever/Pyrexia reported (Yes/No)
Fever/pyrexia (Percentage of all grade)
Fever/pyrexia (Percentage of grade 1/2)
Fever/pyrexia (Percentage of grade 3/4)
Fever/pyrexia (Percentage of grade 5)
Perforation reported (Yes/No)
Perforation (Percentage of all grade)
Perforation (Percentage of grade 1/2)
Perforation (Percentage of grade 3/4)
Perforation (Percentage of grade 5)
AST elevation reported (Yes/No)
AST elevation (Percentage of all grade)
AST elevation (Percentage of grade 1/2)
AST elevation (Percentage of grade 3/4)
AST elevation (Percentage of grade 5)
ALT elevation reported (Yes/No)
ALT elevation (Percentage of all grade)
ALT elevation (Percentage of grade 1/2)
ALT elevation (Percentage of grade 3/4)

ALT elevation (Percentage of grade 5)
Immune related other AE reported (Yes/No)
Immune related other AE (Percentage of all grade)
Type of immune related AE (free text)
Thyroid dysfunction reported (Yes/No)
Thyroid dysfunction (Percentage of all grade)
Thyroid dysfunction (Percentage of grade 1/2)
Thyroid dysfunction (Percentage of grade 3/4)
Thyroid dysfunction (Percentage of grade 5)
Diarrhea reported (Yes/No)
Diarrhea (Percentage of all grade)
Diarrhea (Percentage of grade 1/2)
Diarrhea (Percentage of grade 3/4)
Diarrhea (Percentage of grade 5)
Fatigue reported (Yes/No)
Fatigue (Percentage of all grade)
Fatigue (Percentage of grade 1/2)
Fatigue (Percentage of grade 3)
Rash/Skin toxicity reported (Yes/No)
Rash/Skin toxicity (Percentage of all grade)
Rash/ Skin toxicity (Percentage of grade 1/2)
Rash/Skin toxicity (Percentage of grade 3/4)
Rash/Skin toxicity (Percentage of grade 5)
Pruritus reported (Yes/No)
Pruritus (Percentage of all grade)
Pruritus (Percentage of grade 1/2)
Pruritus (Percentage of grade 3)
Anorexia reported (Yes/No)
Anorexia (Percentage of all grade)
Anorexia (Percentage of grade 1/2)
Anorexia (Percentage of grade 3/4)
Anorexia (Percentage of grade 5)
Other adverse events (free text)
Corticosteroids use reported (Yes/no)
Corticosteroids use reasons
Percentage of corticosteroids
AE leading to dose interruption (percentage of definitive or temporary interruption)
Percentage of AE leading to permanent withdrawal of the drug

AE: adverse event; ALT: Alanine aminotransferase; AST: aspartate aminotransferase; Atezo-Beva: atezolizumab-bevacizumab; HBV: Hepatitis B Virus: HCV: Hepatitis C Virus:

Table S2. Characteristics of the studies (n=30).

Variable	Available data	Median (IQR) or n (%)
**Design**<br>Prospective<br>Retrospective	30	5 (17.0)<br>25 (83.0)
**Center**<br>Monocentric<br>Multicentric	30	10 (33.0)<br>20 (67.0)
**Region**<br>Asia<br>Europe<br>Both	30	27 (90.0)<br>1 (3.3)<br>2 (6.7)
**Atezolizumab-Bevacizumab only as first line (yes)**	28	7 (23.3%)
**Atezolizumab-Bevacizumab only in CP class A (yes)**	28	3 (10.0%)
**Adverse events classification used**<br>CTCAE v.4<br>CTCAE v.5	27	6 (20.0)<br>21 (70.0)
**Patients with cirrhosis**	4	89.00 (71.88-100.00)
**Patients with chronic hepatitis B**	25	19.20 (15.00-25.60)
**Patients with chronic hepatitis C**	25	31.30 (21.70-33.00)
**Patients with metabolic syndrome**	7	25.20 (16.60-26.83)
**Patients with chronic alcohol consumption**	12	22.45 (16.20-26.83)
**Male patients**	30	80.85 (78.80-84.22)
**Age of patients**	27	72 (63.50-73.55)

CP: Child-Pugh. CTCAE: Common Terminology Criteria for Adverse Events. HBV: Hepatitis B Virus. HCV: Hepatitis C Virus.
The two phase Ib and phase III studies were excluded from the calculations of medians (IQR) and numbers (percentages).
Median and IQR are used for descriptive analysis.

Table S3. Association among percentage of each specific adverse event of any grade and prognosis

	Raw data				Exposed Adjusted Incidence Rate			
	Overall Survival (n=11)		Progression-Free Survival (n=15)		Overall Survival (n=3)		Progression-Free Survival (n=4)	
	Coefficient correlation*	p-value	Coefficient correlation*	p-value	Coefficient correlation*	p-value	Coefficient correlation*	p-value
**Hypertension**	0.323	0.43	0.165	0.65	0.406	0.73	0.726	0.48
**Fatigue**	0.594	0.16	0.361	0.34	0.922	0.25	0.985	0.11
**Proteinuria**	-0.137	0.77	0.426	0.25	-0.500	0.67	-0.602	0.59
**Thyroid dysfunction**	-0.105	0.84	0.090	0.85	-0.655	0.55	0.143	0.91
**Rash**	0.422	0.34	0.292	0.44	0.661	0.54	-0.220	0.86
**Bleeding**	0.275	0.55	0.141	0.72	-	-	-	-
**Diarrhea**	0.407	0.50	0.190	0.72	-	-	-	-
**Anorexia**	0.434	0.56	-0.411	0.42	-	-	-	-

*Partial correlation between mOS and adverse events (for which we have at least 50% of data available) controlling for the effect of number of patients of survival analysis.

Table S4. List of the 30 Studies included in the analysis

Author	Journal	Retrospective/ Prospective	Monocentric/ Multicentric	Country	Number of patients	Corticosteroid prescription	Corticosteroid prescription in all population included	Liver-related adverse event reported	Liver-related adverse event terminology
Kuzuya et al (1)	Cancer Diagnosis & Prognosis	Retrospective	Monocentric	Asia	23	NA	NA	Yes	Cholangitis
Sho et al (2)	Hepatology Research	Retrospective	Multicentric	Asia	58	No	NA	Yes	AST elevation, ALT elevation, GGT increase, blood bilirubine increase
Ando et al (3)	Cancers (MDPI)	Retrospective	Monocentric	Asia	40	Yes	7.50%	Yes	Liver dysfunction
Hayakawa et al (4)	Investigational New Drugs	Retrospective	Monocentric	Asia	52	Yes	7.70%	Yes	Transaminases increase
Eso et al (5)	Current Oncology	Prospective	Monocentric	Asia	40	NA	NA	No	NA
Chuma et al (6)	Hepatology Research	Retrospective	Multicentric	Asia	94	NA	NA	Yes	AST elevation, ALT elevation
Yang-Cheng et al (7)	In Vivo	Retrospective	Multicentric	Asia	35	NA	NA	No	NA
Wang et al (8)	Cancers (MDPI)	Retrospective	Monocentric	Asia	48	NA	NA	Yes	Aspartate/Alanine aminotransferase increase
Maesaka et al (9)	Hepatology Research	Prospective	Multicentric	Asia	66	NA	NA	Yes	Increased AST or ALT
Teng et al (10)	American Journal of Cancer Research	Retrospective	Monocentric	Asia	89	NA	NA	Yes	AST elevation, ALT elevation
Tomonari et al (11)	Cancer Medicine	Retrospective	Multicentric	Asia	71	NA	NA	Yes	Increased transaminase
Ochi et al (12)	Hepatology Research	Retrospective	Multicentric	Asia	242	NA	NA	Yes	Liver injury
Sugimoto et al (13)	Medicine	Prospective	Multicentric	Asia	31	Yes	NA	Yes	AST elevation, ALT elevation, Blood bilirubin increase, HCC rupture
Niizeki et al (14)	Targeted Oncology	Retrospective	Multicentric	Asia	152	NA	NA	Yes	Liver disorder
Nakagawa et al (15)	Cancer	Retrospective	Multicentric	Asia	123	NA	NA	Yes	AST elevation, ALT elevation, Blood bilirubin increase
Casadei-Gardini et al (16)	European Journal of Cancer	Retrospective	Multicentric	Both	864	NA	NA	No	NA
Charonpongsuntorn et al (17)	JCO Global Oncology	Prospective	Multicentric	Asia	30	NA	NA	Yes	Aspartate aminotransferase/alanine aminotransferase elevation
Unome et al (18)	Cancers (MDPI)	Retrospective	Multicentric	Asia	69	NA	NA	Yes	Liver dysfunction
Cheon et al (19)	Therapeutic Advances in Medical Oncology	Retrospective	Multicentric	Asia	169	NA	NA	Yes	AST elevation, ALT elevation, Hyperbilirubinemia
Zeng et al (20)	Frontiers in Immunology	Retrospective	Monocentric	Asia	30	NA	NA	Yes	Increased ALT or AST, Increased blood bilirubin
Matoya et al (21)	Hepatology Research	Retrospective	Multicentric	Asia	110	NA	NA	Yes	ALT elevation
Kulkarni et al (22)	Journal of Cilinical and Experimental Hepatology	Retrospective	Multicentric	Asia	67	NA	NA	Yes	Rise in AST/ALT, rise in bilirubin >3 mg/dl
Tokunaga et al (23)	Cancers (MDPI)	Retrospective	Multicentric	Asia	100	NA	NA	Yes	AST elevation, ALT elevation, hepatitis
Jost-Brinkmann et al (24)	AP&T Alimentary Pharmacology & Therapeutics	Retrospective	Monocentric	Europe	100	NA	NA	No	NA
Takaki et al (25)	Investigational New Drugs	Retrospective	Multicentric	Asia	268	NA	NA	Yes	Increased AST or AL, increased bilirubin level
Fukushima et al (26)	The Oncolongist	Retrospective	Multicentric	Asia	150	Yes	14.60%	Yes	Liver injury, Hepatic irAE
Yano et al (27)	Journal of Gastroenterology and Hepatology	Retrospective	Multicentric	Asia	136	NA	NA	Yes	Liver dysfunction
Tada et al (28)	Journal of Gastroenterology	Retrospective	Multicentric	Asia	506	NA	NA	Yes	Hepatic examination abnormality
Takada et al (29)	Hepatology Research	Retrospective	Monocentric	Asia	61	Yes	13.10%	Yes	Liver injury
Larrey et al (30)	Liver International	Prospective	Monocentric	Europe	43	NA	NA	NA	NA

CTCAE: Common Terminology Criteria for Adverse Events. HBV: Hepatitis B Virus. HCV: Hepatitis C Virus. DCR: Disease Control Rate. ORR: Objective Response Rate. OS: Overall Survival. PFS: Progression-Free Survival. QOL: Quality Of Life. TTP: Time To Progression.

Table S5. Description of the bleeding events across the 30 studies

Variable	N (%)
Bleeding event reported	20 (66.7)
Type of bleeding*	
Not specified	6 (30.0)
Extra-GI^	3 (15.0)
GI	2 (10.0)
Extra-GI and GI	2 (10.0)
Extra-GI and variceal	1 (5.0)
Variceal and GI	2 (10.0)
Variceal	1 (5.0)

* The percentages are calculated based on the total number of studies that reported bleeding (n=20).
Extra-GI: the bleeding described in the study occurred outside the gastrointestinal system (epistaxis, bronchopulmonary). GI: gastrointestinal tract bleeding without any details regarding potential variceal bleeding. Variceal: studies that reported considering variceal bleeding among the types of bleeding.

Table S6. Characteristics of the studies included in the analysis of intra- and inter-cohort variability (n=36)

References	Journal	Retrospective/ Prospective	Monocentric/ Multicentric	Country	Number of patients	Primary endpoint	Median OS	Median PFS	Child Pugh B/C	HBV (%)	HCV (%)	Metabolic (%)	Alcohol (%)	Male (%)	Median age (years)	Adverse events evaluation method
Iwamoto et al[%] (31)	Cancers (MDPI)	Retrospective	Multicentric	Asia	51	ORR, PFS, DCR	Not reached	5,4	7,8	13,7	37,2	NA	NA	88,2	71	CTCAE v5.0
Hiraoka et al[$] (32)	Cancer Reports	Retrospective	Multicentric	Asia	171	ORR	NA	NA	4,1	15,7	35	NA	18,1	84,2	73	CTCAE v4.0
Hiraoka et al[$] (33)	Hepatology Research	Retrospective	Multicentric	Asia	95	ORR	NA	8,0	0	12,6	26,3	NA	21	76,8	76	CTCAE v5.0
Cheon et al[^] (34)	Liver International	Retrospective	Multicentric	Asia	121	OS, PFS, ORR	Not reached	6,5	0	76,9	5	NA	NA	83,5	61	CTCAE v5.0
Hatanaka et al[$](35)	Hepatology Research	Retrospective	Multicentric	Asia	239	OS, PFS, ORR	NA	NA	0	18	30,1	NA	19,2	81,6	73	CTCAE v5.0
Tiago de Castro et al*(36)	Therapeutic Advances in Medical Oncology	Retrospective	Multicentric	Europe	147	ORR, DCR	NA	NA	27,9	8,2	25,9	23,1	26,5	85	68,7	CTCAE v5.0
Himmelsbach et al* (37)	Cancers (MDPI)	Retrospective	Multicentric	Europe	66	OS, PFS, ORR, Safety	NA	NA	42,4	13,6	21,2	27,3	37,9	81,8	65	CTCAE v4.0
Kim et al[^](38)	Cancers (MDPI)	Retrospective	Multicentric	Asia	86	OS, PFS, ORR	Not reached	5,7	4,7	72,1	2,5	NA	12,8	81,4	62	CTCAE v5.0
D'Alessio et al* (39)	Hepatology	Retrospective	Multicentric	Asia	202	OS, PFS, ORR, DCR, Safety	14,9	6,8	24	17	36	11,3	19,3	85	69	CTCAE v5.0
Teng et al[#](10)	American Journal of Cancer Research	Retrospective	Monocentric	Asia	89	Biomarker OS	NA	NA	14,6	77,5	11,2	NA	NA	84,3	61,3	CTCAE v5.0
Tada et al[$](40)	Cancer Medicine	Retrospective	Multicentric	Asia	317	OS, PFS, Safety	NA	NA	5,6	17,3	33,1	NA	NA	81,4	74	CTCAE v5.0
Tanaka et al[$] (41)	Hepatology Research	Retrospective	Multicentric	Asia	427	ORR	NA	NA	6,5	17,3	34,1	NA	17,9	80,5	74	CTCAE v5.0
Hatanaka et al[$](35)	Hepatology International	Retrospective	Multicentric	Asia	297	Biomarker, OS, PFS	NA	NA	6,1	16,8	33,3	20,2	19,2	81,8	73	CTCAE v5.0
Chon et al[^](42)	Cancer Medicine	Retrospective	Multicentric	Asia	121	Biomarker, OS, PFS	Not reached	5,7	10	60,6	5,8	NA	15,7	82,6	63	CTCAE v5.0
Shimose et al[%](43)	Cancers (MDPI)	Retrospective	Multicentric	Asia	130	OS, Safety	18,2	6,4	NA	14,6	46,1	16,1	23	78,4	72,5	CTCAE v5.0
Vithayathil et al*(44)	Liver International	Retrospective	Multicentric	Asia	191	OS, PFS, ORR, DCR	NA	NA	23	19,4	37,7	13,1	38,2	84,3	68,4	CTCAE v5.0
Fulgenzi et al*(45)	European Journal of Cancer	Prospective	Multicentric	Both	296	OS, PFS, ORR	15,7	6,9	0	40,6	25,3	NA	NA	82,7	66	CTCAE v5.0
Jost-Brinkmann et al[~](24)	AP&T Alimentary Pharmacology & Therapeutics	Retrospective	Monocentric	Europe	100	OS, PFS, ORR, DCR, Safety, TTP	NA	6,3	39	NA	NA	NA	NA	87	67	CTCAE v5.0
Rimini et al*(46)	ESMO Journal	Retrospective	Multicentric	Both	190	OS	12,1	5,5	5,8	NA	NA	43,1	NA	78,4	NA	CTCAE v5.0
Hatanaka et al[$](47)	Cancer Medicine	Retrospective	Multicentric	Asia	252	PFS, ORR, DCR, Safety	Not reached	NA	0	19,2	31,9	17,3	18,3	80,2	73	CTCAE v5.0
Niizeki et al[%](14)	Targeted Oncology	Retrospective	Multicentric	Asia	152	OS, PFS, ORR	Not reached	8,3	NA	13,6	39,1	NA	NA	76,4	73	CTCAE v5.0
Casadei-Gardini et al*(16)	European Journal of Cancer	Retrospective	Multicentric	Both	864	OS, TTP, Safety	16,4	NA	7,2	23,5	31,3	6,8	NA	79,9	72	CTCAE v5.0
Wu et al*(48)	Cancers (MDPI)	Retrospective	Multicentric	Both	296	Biomarker, OS, PFS	NA	NA	0	NA	NA	NA	NA	82,7	66	CTCAE v5.0
Sinner et al (49)[~]	Cancers (MDPI)	Retrospective	Multicentric	Europe	50	OS, PFS, ORR, DCR	16,0	7,1	27	12	21	23	25	82	65	CTCAE v5.0
Chung-Wei Su et al[#](50)	Cancer Medicine	Retrospective	Monocentric	Asia	46	OS, PFS, ORR	Not reached	5,3	13	NA	NA	NA	NA	82,6	61,2	CTCAE v5.0
Hiraoka et al[$](51)	Liver Cancer	Retrospective	Multicentric	Asia	229	Safety	NA	NA	7,4	14,4	35,4	NA	17,4	81,2	74	CTCAE v5.0
Tada et al[$](52)	Cancer Medicine	Retrospective	Multicentric	Asia	421	Biomarker, OS, PFS	17,8	6.7	7,4	16,1	34,2	NA	NA	80,8	74	CTCAE v5.0
Tada et al[$](53)	Cancer Medicine	Retrospective	Multicentric	Asia	263	OS, PFS, Safety	Not reached	7,1	7,6	15,6	35,7	NA	NA	79,1	74	CTCAE v5.0
Cheon et al[^](19)	Therapeutic Advances in Medical Oncology	Retrospective	Multicentric	Asia	169	OS, PFS, ORR	NA	NA	17,7	66,8	6,5	NA	14,7	82,2	61	CTCAE v5.0
Rimini et al*(54)	Journal of Cancer Research and Clinical Oncology	Retrospective	Multicentric	Both	65	OS, PFS	8,2	6,9	100	NA	NA	32	NA	89	NA	CTCAE v5.0
Vithayathil et al*(55)	Hepatology International	Retrospective	Multicentric	Both	191	OS, PFS, ORR, DCR	14,9	6,7	23	19,4	37,7	13,1	38,2	84,3	68,4	CTCAE v5.0
Tada et al[$](28)	Journal of Gastroenterology and Hepatology	Retrospective	Multicentric	Asia	430	OS, PFS	20	7.0	0	19	33,2	NA	NA	81,1	74	CTCAE v5.0
Tada et al[$](56)	Journal of Gastroenterology	Retrospective	Multicentric	Asia	506 (EGV group = 151, non-EGV group = 355)	ORR	NA	NA	10,6	16,8	33	NA	21,9	77,8	74	CTCAE v5.0
Ohama et al[$](57)	Oncology	Retrospective	Multicentric	Asia	29	OS, PFS	5,7	5,0	100	NA	NA	NA	NA	79,3	72	CTCAE v5.0
Kim et al[^](38)	Cancers (MDPI)	Retrospective	Multicentric	Asia	114	OS, PFS	5,8	4,3	7	64,04	3,51	NA	17,54	86,8	63,3	NA

Tada et al$(58)	Liver International	Retrospective	Multicentric	Asia	177	PFS	24,0	10,8	7,9	10,2	33,3	NA	NA	75,7	74	CTCAE v5.0

CTCAE: Common Terminology Criteria for Adverse Events. HBV: Hepatitis B Virus. HCV: Hepatitis C Virus. DCR: Disease Control Rate. ORR: Objective Response Rate. OS: Overall Survival. PFS: Progression-Free Survival. QOL: Quality Of Life. TTP: Time To Progression.

*[$]RELPEC. [%] Japan (not RELPEC). [^] Korea *Collaborative European and non-European centers. [#]Taiwain. ~ German (not included in the collaborative European and non-European group).*

Supplementary figure legends

Fig. S1. Percentage of studies reporting data on frequency of each adverse event of any grade and prognosis

Each bar illustrates the percentage of studies reporting both data of each specific adverse event of any grade and (A) median overall survival (n=11) or (B) median progression free survival (n=15). Each bar in (C) and (D) depicts the percentage of studies for which we were able to calculate the Exposed Adjusted Incidence Rate and that also reported median overall survival (n=3) and median progression-free survival (n=4), respectively.

Fig. S2. Percentage of adverse events reported in studies published by the same research group (5 studies or more per research group)

Each scatterplot illustrates the percentage of a specific adverse event (raw data) reported in studies published by the same research group, regardless of the severity grade. (A) RELPEC group (14 studies) (B) European and non-European cohorts (10 studies) (C) Korean cohorts (5 studies).

Fig. S3. Percentage of adverse events reported in studies published by the same research group (less than 5 studies per research group)

Each scatterplot illustrates the percentage of a specific adverse event (raw data) reported in studies published by the same research group, regardless of the severity grade (A) Taiwanese cohorts (2 studies) (B) German cohorts (2 studies) (C) Japanese cohorts not included in RELPEC group (3 studies).

Supplementary references

1. Kuzuya T, Kawabe N, Hashimoto S, et al. Initial Experience of Atezolizumab Plus Bevacizumab for Advanced Hepatocellular Carcinoma in Clinical Practice. CDP. 2021 May 3;1(2):83–8.
2. Sho T, Suda G, Ogawa K, et al. Early response and safety of atezolizumab plus bevacizumab for unresectable hepatocellular carcinoma in patients who do not meet IMbrave150 eligibility criteria. Hepatology Research. 2021 Sep;51(9):979–89.
3. Ando Y, Kawaoka T, Kosaka M, et al. Early Tumor Response and Safety of Atezolizumab Plus Bevacizumab for Patients with Unresectable Hepatocellular Carcinoma in Real-World Practice. Cancers. 2021 Aug 5;13(16):3958.
4. Hayakawa Y, Tsuchiya K, Kurosaki M, et al. Early experience of atezolizumab plus bevacizumab therapy in Japanese patients with unresectable hepatocellular carcinoma in real-world practice. Invest New Drugs. 2022 Apr;40(2):392–402.
5. Eso Y, Takeda H, Taura K, et al. Pretreatment Neutrophil-to-Lymphocyte Ratio as a Predictive Marker of Response to Atezolizumab Plus Bevacizumab for Hepatocellular Carcinoma. Current Oncology. 2021 Oct 14;28(5):4157–66.
6. Chuma M, Uojima H, Hattori N, et al. Safety and efficacy of atezolizumab plus bevacizumab in patients with unresectable hepatocellular carcinoma in early clinical practice: A multicenter analysis. Hepatology Research. 2022 Mar;52(3):269–80.

7. Lee YC, Huang WT, Lee MY, et al. Bevacizumab and Atezolizumab for Unresectable Hepatocellular Carcinoma: Real-world Data in Taiwan-Tainan Medical Oncology Group H01 Trial. In Vivo. 2023;37(1):454–60.

8. Wang JH, Chen YY, Kee KM, et al. The Prognostic Value of Neutrophil-to-Lymphocyte Ratio and Platelet-to-Lymphocyte Ratio in Patients with Hepatocellular Carcinoma Receiving Atezolizumab Plus Bevacizumab. Cancers. 2022 Jan 11;14(2):343.

9. Maesaka K, Sakamori R, Yamada R, et al. Comparison of atezolizumab plus bevacizumab and lenvatinib in terms of efficacy and safety as primary systemic chemotherapy for hepatocellular carcinoma. Hepatology Research. 2022 Jul;52(7):630–40.

10. Teng W, Lin CC, Su CW, et al. Combination of CRAFITY score with Alpha-fetoprotein response predicts a favorable outcome of atezolizumab plus bevacizumab for unresectable hepatocellular carcinoma. Am J Cancer Res. 2022;12(4):1899–911.

11. Tomonari T, Tani J, Sato Y, et al. Initial therapeutic results of atezolizumab plus bevacizumab for unresectable advanced hepatocellular carcinoma and the importance of hepatic functional reserve. Cancer Medicine. 2023 Feb;12(3):2646–57.

12. Ochi H, Kurosaki M, Joko K, et al. Usefulness of neutrophil-to-lymphocyte ratio in predicting progression and survival outcomes after atezolizumab–bevacizumab treatment for hepatocellular carcinoma. Hepatology Research. 2023 Jan;53(1):61–71.

13. Sugimoto R, Satoh T, Ueda A, et al. Atezolizumab plus bevacizumab treatment for unresectable hepatocellular carcinoma progressing after molecular targeted therapy: A multicenter prospective observational study. Medicine. 2022 Oct 7;101(40):e30871.

14. Niizeki T, Tokunaga T, Takami Y, et al. Comparison of Efficacy and Safety of Atezolizumab Plus Bevacizumab and Lenvatinib as First-Line Therapy for Unresectable Hepatocellular Carcinoma: A Propensity Score Matching Analysis. Targ Oncol. 2022 Nov;17(6):643–53.

15. Nakagawa M, Inoue M, Ogasawara S, et al. Clinical effects and emerging issues of atezolizumab plus bevacizumab in patients with advanced hepatocellular carcinoma from Japanese real-world practice. Cancer. 2023 Feb 15;129(4):590–9.

16. Casadei-Gardini A, Rimini M, Tada T, et al. Atezolizumab plus bevacizumab versus lenvatinib for unresectable hepatocellular carcinoma: a large real-life worldwide population. European Journal of Cancer. 2023 Feb;180:9–20.

17. Charonpongsuntorn C, Tanasanvimon S, Korphaisarn K, et al. Efficacy, Safety, and Patient-Reported Outcomes of Atezolizumab Plus Bevacizumab for Unresectable Hepatocellular Carcinoma in Thailand: A Multicenter Prospective Study. JCO Global Oncology. 2022 Dec;(8):e2200205.

18. Unome S, Imai K, Takai K, et al. Changes in ALBI Score and PIVKA-II within Three Months after Commencing Atezolizumab Plus Bevacizumab Treatment Affect Overall Survival in Patients with Unresectable Hepatocellular Carcinoma. Cancers. 2022 Dec 10;14(24):6089.

19. Cheon J, Kim H, Kim HS, et al. Atezolizumab plus bevacizumab in patients with child–Pugh B advanced hepatocellular carcinoma. Ther Adv Med Oncol. 2023 Jan;15:175883592211485.

20. Zeng H, Xu Q, Wang J, et al. The effect of anti-PD-1/PD-L1 antibodies combined with VEGF receptor tyrosine kinase inhibitors versus bevacizumab in unresectable hepatocellular carcinoma. Front Immunol. 2023 Jan 23;14:1073133.

21. Matoya S, Suzuki T, Matsuura K, et al. The neutrophil-to-lymphocyte ratio at the start of the second course during atezolizumab plus bevacizumab therapy predicts therapeutic efficacy in patients with advanced hepatocellular carcinoma: A multicenter analysis. Hepatology Research. 2023 Jun;53(6):511–21.

22. Kulkarni AV, Krishna V, Kumar K, et al. Safety and Efficacy of Atezolizumab-Bevacizumab in Real World: The First Indian Experience. Journal of Clinical and Experimental Hepatology. 2023 Jul;13(4):618–23.

23. Tokunaga T, Tateyama M, Kondo Y, et al. Therapeutic Modifications without Discontinuation of Atezolizumab Plus Bevacizumab Therapy Are Associated with Favorable Overall Survival and Time to Progression in Patients with Unresectable Hepatocellular Carcinoma. Cancers. 2023 Mar 2;15(5):1568.

24. Jost-Brinkmann F, Demir M, Wree A, et al. Atezolizumab plus bevacizumab in unresectable hepatocellular carcinoma: Results from a German real-world cohort. Aliment Pharmacol Ther. 2023 Jun;57(11):1313–25.

25. Takaki S, Kurosaki M, Mori N, et al. Effects on survival of the adverse event of atezolizumab plus bevacizumab for hepatocellular carcinoma: a multicenter study

by the Japan Red Cross Liver Study Group. Invest New Drugs. 2023 Apr;41(2):340–9.

26. Fukushima T, Morimoto M, Kobayashi S, et al. Association Between Immune-Related Adverse Events and Survival in Patients with Hepatocellular Carcinoma Treated With Atezolizumab Plus Bevacizumab. The Oncologist. 2023 Jul 5;28(7):e526–33.

27. Yano Y, Yamamoto A, Mimura T, et al. Factors associated with the response to atezolizumab/bevacizumab combination therapy for hepatocellular carcinoma. JGH Open. 2023 Jul;7(7):476–81.

28. Tada T, Kumada T, Hiraoka A, et al. Impact of first-line systemic therapy with atezolizumab plus bevacizumab in patients with hepatocellular carcinoma. J of Gastro and Hepatol. 2023 Aug;38(8):1389–97.

29. Takada H, Yamashita K, Osawa L, et al. Significance of the autoantibody assay in predicting the development of immune-related adverse events in patients receiving atezolizumab plus bevacizumab combination therapy for unresectable hepatocellular carcinoma. Hepatology Research. 2024 Feb;54(2):162–73.

30. Larrey E, Campion B, Evain M, et al. A history of variceal bleeding is associated with further bleeding under atezolizumab–bevacizumab in patients with HCC. Liver International. 2022 Dec;42(12):2843–54.

31. Iwamoto H, Shimose S, Noda Y, et al. Initial Experience of Atezolizumab Plus Bevacizumab for Unresectable Hepatocellular Carcinoma in Real-World Clinical Practice. Cancers. 2021 Jun 3;13(11):2786.

32. Hiraoka A, Kumada T, Tada T, et al. Atezolizumab plus bevacizumab treatment for unresectable hepatocellular carcinoma: Early clinical experience. Cancer Reports. 2022 Feb;5(2):e1464.

33. Hiraoka A, Kumada T, Tada T, et al. Early experience of atezolizumab plus bevacizumab treatment for unresectable hepatocellular carcinoma BCLC-B stage patients classified as beyond up to seven criteria – Multicenter analysis. Hepatology Research. 2022 Mar;52(3):308–16.

34. Cheon J, Yoo C, Hong JY, et al. Efficacy and safety of atezolizumab plus bevacizumab in Korean patients with advanced hepatocellular carcinoma. Liver International. 2022 Mar;42(3):674–81.

35. Hatanaka T, Kakizaki S, Hiraoka A, et al. Prognostic impact of C-reactive protein and alpha-fetoprotein in immunotherapy score in hepatocellular carcinoma patients treated with atezolizumab plus bevacizumab: a multicenter retrospective study. Hepatol Int. 2022 Oct;16(5):1150–60.

36. De Castro T, Jochheim LS, Bathon M, et al. Atezolizumab and bevacizumab in patients with advanced hepatocellular carcinoma with impaired liver function and prior systemic therapy: a real-world experience. Ther Adv Med Oncol. 2022 Jan;14:175883592210802.

37. Himmelsbach V, Pinter M, Scheiner B, et al. Efficacy and Safety of Atezolizumab and Bevacizumab in the Real-World Treatment of Advanced Hepatocellular Carcinoma: Experience from Four Tertiary Centers. Cancers. 2022 Mar 28;14(7):1722.

38. Kim J, Nam HC, Kim CW, et al. Comparative Analysis of Atezolizumab Plus Bevacizumab and Hepatic Artery Infusion Chemotherapy in Unresectable Hepatocellular Carcinoma: A Multicenter, Propensity Score Study. Cancers. 2023 Aug 24;15(17):4233.

39. D'Alessio A, Fulgenzi CAM, Nishida N, et al. Preliminary evidence of safety and tolerability of atezolizumab plus bevacizumab in patients with hepatocellular carcinoma and Child-Pugh A and B cirrhosis: A real-world study. Hepatology. 2022 Oct;76(4):1000–12.

40. Tada T, Kumada T, Hiraoka A, et al. Safety and efficacy of atezolizumab plus bevacizumab in elderly patients with hepatocellular carcinoma: A multicenter analysis. Cancer Medicine. 2022 Oct;11(20):3796–808.

41. Tanaka T, Hiraoka A, Tada T, et al. Therapeutic efficacy of atezolizumab plus bevacizumab treatment for unresectable hepatocellular carcinoma in patients with Child-Pugh class A or B liver function in real-world clinical practice. Hepatology Research. 2022 Sep;52(9):773–83.

42. Chon YE, Cheon J, Kim H, et al. Predictive biomarkers of survival in patients with advanced hepatocellular carcinoma receiving atezolizumab plus bevacizumab treatment. Cancer Medicine. 2023 Feb;12(3):2731–8.

43. Shimose S, Iwamoto H, Tanaka M, et al. Association between Adverse Events and Prognosis in Patients with Hepatocellular Carcinoma Treated with Atezolizumab Plus Bevacizumab: A Multicenter Retrospective Study. Cancers. 2022 Sep 1;14(17):4284.

44. Vithayathil M, D'Alessio A, Fulgenzi CAM, et al. Impact of older age in patients receiving atezolizumab and bevacizumab for hepatocellular carcinoma. Liver International. 2022 Nov;42(11):2538–47.

45. Fulgenzi CAM, Cheon J, D'Alessio A, et al. Reproducible safety and efficacy of atezolizumab plus bevacizumab for HCC in clinical practice: Results of the AB-real study. European Journal of Cancer. 2022 Nov;175:204–13.

46. Rimini M, Rimassa L, Ueshima K, et al. Atezolizumab plus bevacizumab versus lenvatinib or sorafenib in non-viral unresectable hepatocellular carcinoma: an international propensity score matching analysis. ESMO Open. 2022 Dec;7(6):100591.

47. Hatanaka T, Kakizaki S, Hiraoka A, et al. Comparative efficacy and safety of atezolizumab and bevacizumab between hepatocellular carcinoma patients with viral and non-viral infection: A Japanese multicenter observational study. Cancer Medicine. 2023 Mar;12(5):5293–303.

48. Wu YL, Fulgenzi CAM, D'Alessio A, et al. Neutrophil-to-Lymphocyte and Platelet-to-Lymphocyte Ratios as Prognostic Biomarkers in Unresectable Hepatocellular Carcinoma Treated with Atezolizumab plus Bevacizumab. Cancers. 2022 Nov 26;14(23):5834.

49. Sinner F, Pinter M, Scheiner B, et al. Atezolizumab Plus Bevacizumab in Patients with Advanced and Progressing Hepatocellular Carcinoma: Retrospective Multicenter Experience. Cancers. 2022 Dec 2;14(23):5966.

50. Su CW, Teng W, Lin PT, et al. Similar efficacy and safety between lenvatinib versus atezolizumab plus bevacizumab as the first-line treatment for unresectable hepatocellular carcinoma. Cancer Med. 2023 Mar;12(6):7077–89.

51. Hiraoka A, Kumada T, Tada T, et al. Relationship of Atezolizumab plus Bevacizumab Treatment with Muscle Volume Loss in Unresectable Hepatocellular Carcinoma Patients: Multicenter Analysis. Liver Cancer. 2023;12(3):209–17.

52. Tada T, Kumada T, Hiraoka A, et al. New prognostic system based on inflammation and liver function predicts prognosis in patients with advanced unresectable hepatocellular carcinoma treated with atezolizumab plus bevacizumab: A validation study. Cancer Medicine. 2023 Mar;12(6):6980–93.

53. Tada T, Kumada T, Hiraoka A, et al. Adverse events as potential predictive factors of therapeutic activity in patients with unresectable hepatocellular carcinoma treated with atezolizumab plus bevacizumab. Cancer Medicine. 2023 Apr;12(7):7772–83.

54. Rimini M, Persano M, Tada T, et al. Survival outcomes from atezolizumab plus bevacizumab versus Lenvatinib in Child Pugh B unresectable hepatocellular carcinoma patients. J Cancer Res Clin Oncol. 2023 Aug;149(10):7565–77.

55. Vithayathil M, D’Alessio A, Fulgenzi CAM, et al. Impact of body mass index in patients receiving atezolizumab plus bevacizumab for hepatocellular carcinoma. Hepatol Int. 2023 Aug;17(4):904–14.

56. Tada F, Hiraoka A, Tada T, et al. Efficacy and safety of atezolizumab plus bevacizumab treatment for unresectable hepatocellular carcinoma patients with esophageal–gastric varices. J Gastroenterol. 2023 Nov;58(11):1134–43.

57. Ohama H, Hiraoka A, Tada T, et al. Comparison between Atezolizumab Plus Bevacizumab and Lenvatinib for Hepatocellular Carcinoma in Patients with Child-Pugh Class B in Real-World Clinical Settings. Oncology. 2023;101(9):542–52.

58. Tada T, Kumada T, Hiraoka A, et al. Comparison of prognostic impact of atezolizumab plus bevacizumab versus lenvatinib in patients with intermediate-stage hepatocellular carcinoma. Liver International. 2024 Jan;44(1):113–24.
